# mirPRo–a novel standalone program for differential expression and variation analysis of miRNAs

**DOI:** 10.1038/srep14617

**Published:** 2015-10-05

**Authors:** Jieming Shi, Min Dong, Lei Li, Lin Liu, Agustin Luz-Madrigal, Panagiotis A. Tsonis, Katia Del Rio-Tsonis, Chun Liang

**Affiliations:** 1 Department of Biology, Miami University and Center for Visual Sciences at Miami University, Oxford, Ohio 45056, USA; 2 Department of Biology, University of Dayton and Center for Tissue Regeneration and Engineering at the University of Dayton (TREND), Dayton, Ohio 45469, USA; 3 Department of Computer Science and Software Engineering, Miami University, Oxford, Ohio 45056, USA

## Abstract

Being involved in many important biological processes, miRNAs can regulate gene expression by targeting mRNAs to facilitate their degradation or translational inhibition. Many miRNA sequencing studies reveal that miRNA variations such as isomiRs and “arm switching” are biologically relevant. However, existing standalone tools usually do not provide comprehensive, detailed information on miRNA variations. To deepen our understanding of miRNA variability, we developed a new standalone tool called “mirPRo” to quantify known miRNAs and predict novel miRNAs. Compared with the most widely used standalone program, miRDeep2, mirPRo offers several new functions including read cataloging based on genome annotation, optional seed region check, miRNA family expression quantification, isomiR identification and categorization, and “arm switching” detection. Our comparative data analyses using three datasets from mouse, human and chicken demonstrate that mirPRo is more accurate than miRDeep2 by avoiding over-counting of sequence reads and by implementing different approaches in adapter trimming, mapping and quantification. mirPRo is an open-source standalone program (https://sourceforge.net/projects/mirpro/).

MicroRNAs (miRNAs) are short non-coding RNAs (~22 *nt* in length) that regulate gene expression by binding mRNAs to facilitate their degradation or translational inhibition^1^. In animals, miRNAs target mRNAs through a complementary binding between their seed regions (ranging from 2 to 8 *nt*) and the 3′-UTRs of targeted mRNAs; in plants they target mRNAs through near-perfect base pairing^1^,^2^,^3^. During miRNA biogenesis, long primary miRNAs (pri-miRNAs) transcribed from the genome fold into hairpins that have two arms (5′ and 3′) and undergo cleavage to form shorter, hairpin-containing precursor miRNAs (pre-miRNAs, ~70–100 *nt* in length)^1^,^4^. Pre-miRNAs are then cleaved into 22-*nt* duplexes^5^. One strand of the duplex is selected as the mature miRNA that will be combined with the RNA-induced silencing complex (RISC)^6^ to participate in mRNA degradation and translational inhibition^7^, whereas the other strand called star strand (miRNA*) is degraded^8^,^9^. The strand of the duplex with the weaker binding at its 5′ end is usually selected as the mature miRNA^3^,^10^, but alternative strand selection, known as “arm switching”, has been found in different tissues and developmental stages^11^,^12^,^13^,^14^. Due to “arm switching”, different mature miRNAs can be generated from either the 5′ or 3′ arm of the same precursor hairpin (pre-miRNA). Known as miRNA variants, or isomiRs, one mature miRNA species can have some distinctive isoforms that vary in length and/or have different 5′ or 3′ ends^15^. This has been commonly reported in deep sequencing studies^16^,^17^. IsomiRs are mainly generated due to imprecise cleavage of pre-miRNAs, RNA editing and non-templated nucleotide addition at 3′ end of miRNAs^1^,^15^,^18^. Such non-templated nucleotide addition was shown to be the common form of miRNA enzymatic modification^19^, and could influence miRNA stability^20^ and target repression^21^.

miRNAs can regulate different biological processes such as cell proliferation, apoptosis, organismal development, tissue differentiation and regeneration^1^,^18^,^22^,^23^,^24^. miRNAs have been found to be the crucial regulators in the oncogenic pathways^25^ and are involved in many diseases^26^,^27^,^28^,^29^. Clearly, miRNA expression profiling analysis in experimental data is important for studying cellular functions and disease mechanisms. Many miRNA analysis tools use miRNA sequencing data to identify known and novel miRNAs and detect their differential expression profiles, *e.g.*, miRDeep2^30^,^31^, omiRas^32^, miRanalyzer^33^ and miRExpress^34^. Among them, miRDeep2 (*i.e.*, the second version of miRDeep^35^) appears to be the most popular program and widely used for quantifying known miRNAs and predicting novel miRNAs^31^,^36^,^37^,^38^,^39^. However, we have discovered that miRDeep2 has the disadvantage of over-counting sequence reads and the inability to deal with mapped reads containing indels, affecting the detection of genetic variants. Moreover, miRDeep2 does not allow close examination of miRNA variations like isomiRs and “arm switching”, which appear to be indispensable to fully understand the biogenesis and biological functions of miRNAs.

In order to accurately quantify miRNAs and their variations, we developed a new standalone program named “mirPRo”, which is implemented in C++ for fast performance and adopts different approaches in adapter trimming, read mapping and miRNA quantification than miRDeep2. Like some existing tools such as IsomiRage^40^, sRNAbench^41^, isomiRex^42^, isomiRID^43^ and SeqBuster^44^, which have implemented isomiR analysis functions, mirPRo possesses isomiR detection capacity that miRDeep2 does not have. Furthermore, mirPRo offers unique functions (*e.g.*, miRNA family expression quantification, read cataloging based on genome annotation, seed region check, and “arm switching” identification) that are not provided by most other existing tools including omiRas^32^, miRanalyzer^33^ and miRExpress^34^. In this article, we first describe the design and implementation of mirPRo, then report data analysis results of three miRNA datasets^45^,^46^ using mirPRo, and finally compare in depth miRNA analysis results between miRDeep2 and mirPRo.

## Results

### Design and implementation of mirPRo

The mirPRo package is composed of a main program (*i.e.*, *mirpro*) and several component programs (see Supplementary Figure S1). Some of these component programs can be utilized independently. For instance, *mirpro_findAdapter* is a generic program for detecting adapter sequences for RNA-Seq data; *mirpro_feature_pro* is designed for cataloging mapped reads in terms of gene annotation; and *mirpro_armSwitch* is a specific program for “arm switching” detection. mirPRo makes use of a few third-party tools (*e.g.*, Novoalign^47^, HTSeq^48^, randfold^49^ and RNAfold^50^), which must be installed as pre-requisite tools. Both the main and component programs allow advanced tuning in their adjustable parameters. For general users, the whole package except *mirpro_armSwitch* can be executed automatically by initiating the main program, which takes one or more FASTQ data as inputs. *mirpro_armSwitch* needs to be invoked separately after the main program has generated results for different treatments or samples.

Starting with raw sequence data, mirPRo first conducts quality filtering on the reads. For efficient read-to-reference mapping, final clean reads in each library/sample are then collapsed in terms of sequence content, with expression numbers counted (*i.e.*, the count of reads that support one collapsed read). For example, if two sequence reads have one nucleotide difference, they are collapsed as two different collapsed reads. Novoalign^47^ is then utilized to map collapsed reads against the pre-miRNAs (hairpins) downloaded from miRBase^51^. Using collapsed-read-to-hairpin mapping results and expression numbers of collapsed reads, we can derive mapping results for individual final clean reads. Novoalign^47^ has the option to report the mappings by the best mapping score for each read. If a read has only one mapping with the best score, it will be treated as a unique mapping (*i.e.*, it is mapped to one hairpin uniquely). Otherwise, a read has non-unique mappings (*i.e.*, it is mapped to different hairpins), each of which must have the same mapping/alignment score.

**Figure 1:**
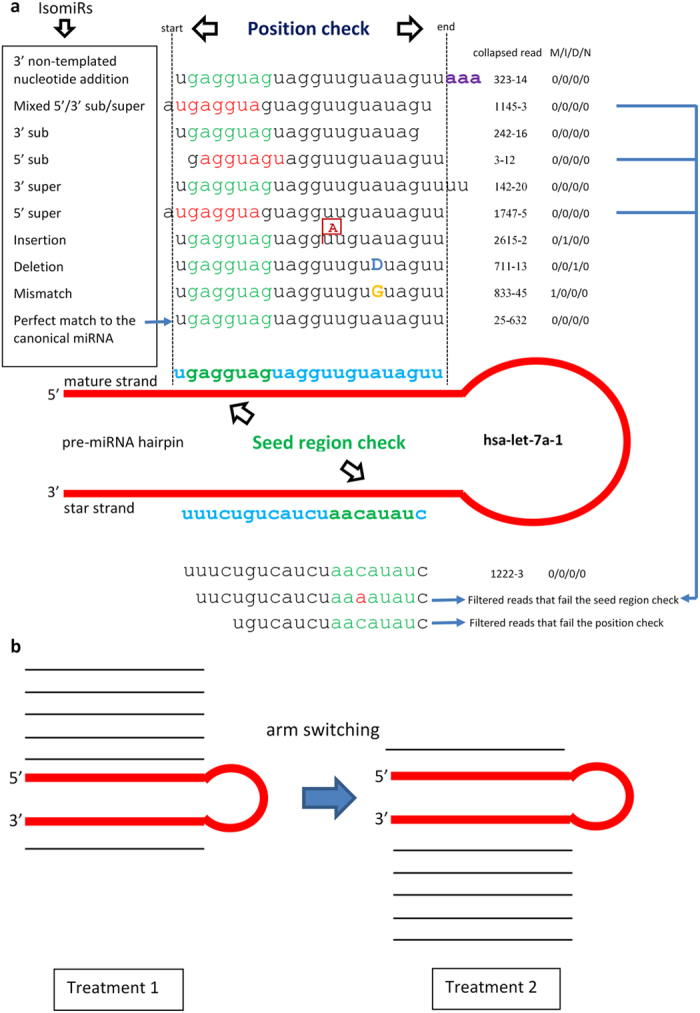
The core algorithms of mirPRo for exploration and quantification of miRNA variants. (**A**) IsomiR identification. Human precursor miRNA “hsa-let-7a-1” is used for illustration, and the collapsed reads are not real data. mirPRo allows base errors (mismatch and indel) and soft clips in read mapping, and permits position check and optional seed region check in mature miRNA quantification. The isomiRs annotated by mirPRo include mature miRNA reads with mismatches, insertions, deletions, or a mixture, with 3′-end non-templated nucleotide addition, and with nucleotide shift (super or sub) at their 5′, 3′ or both ends. Mature miRNA variants: “5 (3) super (sub)” means the reads have 5 (3) end upstream (downstream) nucleotide shift in collapsed-read-to-hairpin mappings. The upper case “D” in the aligned sequence means deletion. The column “collapsed read” has the identifier (“XXX-YYY”) for collapsed reads, where “XXX” is a unique number and “YYY” is the read count. The column “M/I/D/N” represents the number of "mismatches/insertions/deletions/nucleotide N" in the alignment. For the two hairpin arms, most of the collapsed reads are mapped to the 5′ arm while few reads are mapped to the 3′ arm. (**B**) Arm switching detection. More reads are mapped to the 5′ arm of the precursor in treatment 1, while more reads are mapped to the 3′ arm in treatment 2. This indicates that two different mature miRNAs are generated from two different arms of the same precursor in two different treatments (*e.g.*, different tissues).

Relying on both the canonical mature miRNAs and their pre-miRNA (hairpin) sequences downloaded from miRBase^51^, mirPRo performs known miRNA quantification using the algorithm illustrated in Fig. 1. If a final clean read passes both the position check and seed region check, it will be treated as a mature miRNA read. Otherwise, it will be discarded. The position check ensures that the end shift between a mature miRNA read and its canonical mature miRNA is no more than 3 nucleotides, while the seed region check ensures that both a mature miRNA read and its canonical mature miRNA share the same seed region. This algorithm also assures that there is no over-counting problem for mature miRNA reads by evenly dividing read counts among their mapped hairpins, if the reads have non-unique mappings. Based on the pre-miRNA family classification data downloaded from miRBase^51^, mirPRo provides expression quantification for each family by adding all mature RNA reads quantified to relevant mature miRNAs that belong to a given family. mirPRo predicts novel miRNAs using the algorithm from miRDeep2^30^,^31^ with our modifications (see **Methods**).

For miRNA variation analysis, mirPRo is able to detect isomiRs - miRNA variants that are different from their canonical mature miRNAs annotated in miRBase^51^. As shown in Fig. 1, we allowed mismatches, indels and 5′/3′-soft-clips in mapping and 5′/3′ nucleotide position shift in quantification, enabling mirPRo to detect isomiRs. mirPRo can detect potential “arm switching” cases by examining canonical mature miRNAs that have two forms (5p and 3p) and comparing their mature miRNA read counts between different treatments (see **Methods**).

mirPRo can provide cataloging of all clean reads if genome annotation information is provided in GTF format, so that users can understand the overall assessment of miRNA sequencing and alignment quality. We used HTSeq^48^ (see **Methods**) to tally mapped reads in terms of annotated features (*e.g.*, protein coding, miRNA, snRNA, rRNA, snoRNA, and ncRNA genes). mirPRo can generate a sequence file that contains unmapped clean reads in FASTA format, so that the data can be further examined by users to determine potential sequencing contamination sources.

Compared with mirPRo, most of the popular miRNA analysis tools including miRDeep2 do not offer useful functions like “arm switching” detection, miRNA family expression quantification, read cataloging, and seed region check (see Supplementary Table S1). Also, most of these tools do not allow indels in read mapping, affecting accurate quantification of miRNA variations (see below).

### miRNA data analysis by mirPRo

We have used mirPRo to conduct miRNA analysis for three datasets (*i.e.*, mouse, human, and chicken miRNA datasets, see **Methods**). On average, 99.99%, 98.45% and 99.09% of the raw reads in mouse, human and chicken datasets respectively were kept after quality filtering (see Supplementary Table S2). Since the human dataset we downloaded was already clean of adapters, we used an option in mirPRo to skip the adapter trimming process. After adapter trimming, averagely 93.09% (mouse) and 76.81% (chicken) of the raw reads were kept as final clean reads (see Supplementary Table S2). The total numbers of distinctive collapsed reads in mouse, human and chicken datasets are shown in Supplementary Table S3. On average, 85.72% (mouse), 43.86% (human) and 19.35% (chicken) of the raw reads were mapped to their hairpin sequences (see Supplementary Table S2). In particular, 70.09% (mouse), 62.78% (human) and 60.53% (chicken) of the mapped clean reads had unique mappings, whereas 29.91% (mouse), 37.22% (human) and 39.47% (chicken) had non-unique mappings (see Supplementary Table S4). For both unique and non-unique mappings, on average, 77.55% (mouse), 72.72% (human) and 79.61% (chicken) were perfect mappings; 3.54% (mouse), 1.91% (human) and 13.77% (chicken) had mismatches; 0.002% (mouse), 6.24% (human) and 0.003% (chicken) had insertions; 0.07% (mouse), 1.99% (human) and 0.01% (chicken) had deletions; 1.20% (mouse), 2.36% (human) and 0.81% (chicken) had 5′-soft-clips; 16.85% (mouse), 14.49% (human) and 4.83% (chicken) had 3′-soft-clips; and 0.21% (mouse), 0.14% (human) and 0.04% (chicken) had both 5′- and 3′-soft-clips (see Supplementary Table S4).

After known miRNA quantification, on average, 78.14% (mouse), 39.59% (human), and 16.71% (chicken) of the raw reads were counted as mature miRNA reads (see Supplementary Table S2). The detailed statistics of quantification using the three datasets are shown in Supplementary Table S5. On average, 97.98% (mouse), 97.22% (human) and 91.10% (chicken) of the total mapped reads passed the position check, and 91.24% (mouse), 90.23% (human) and 86.23% (chicken) of the total mapped reads passed both the position and seed region checks. On average, 27.53% (mouse), 32.92% (human) and 37.54% (chicken) of the total mapped reads can be mapped to more than one hairpin successfully with the same mapping scores after passing both the position and seed region checks, and their counts were distributed evenly among these different hairpins (see Supplementary Table S5). In miRBase release 21, there are 1915, 2588 and 994 mature miRNAs annotated for mouse, human and chicken respectively; we detected 1230, 954 and 577 different mature miRNAs in mouse, human and chicken datasets accordingly (see Supplementary Table S2). We also detected 760 (mouse), 589 (human) and 426 (chicken) miRNA families whereas there are 1305 (mouse), 1811 (human) and 780 (chicken) miRNA families annotated in miRBase release 21 (see Supplementary Table S2).

As shown in Supplementary Tables S6 and S7, on average, 60.21% (mouse), 52.07% (human) and 58.83% (chicken) of the mature miRNA reads are somewhat different than their canonical mature miRNAs. Interestingly, the most frequent isomiRs appear to be the 3′ super mature miRNA variants: averagely 22.38% (mouse), 19.99% (human) and 33.36% (chicken) of the mature miRNA reads show 3′ super mature miRNA variants (see Fig. 1 and Supplementary Tables S6 and S7). On average, 0.77% (mouse), 0.48% (human) and 6.81% (chicken) of the mature miRNA reads had mismatches in mappings; 0.0006 % (mouse), 4.41% (human) and 0.002% (chicken) had insertions; 0.018% (mouse), 1.20% (human) and 0.004% (chicken) had deletions. In particular, 5.67% (mouse), 13.18% (human) and 27.08% (chicken) of the mature miRNA reads with mismatches only had A-to-G mismatches, which have proved to be the major form of RNA editing events in miRNAs^52^. Due to the seed region check and filtration, we did not find any 5′ super/sub miRNA variants and 5′ non-templated nucleotide additions among three datasets. In contrast, averagely 16.21% (mouse), 6.77% (human) and 1.55% (chicken) of the mature miRNA reads had 3′-end non-templated nucleotide addition. Among the reads with 3′-end non-templated nucleotide addition, 34.29% (mouse), 28.47% (human) and 42.12% (chicken) had one or more uracil nucleotides (or poly(U) tails) at their 3′ ends; 42.62% (mouse), 51.92% (human) and 34.69% (chicken) had one or more adenine nucleotides (or poly(A) tails) at their 3′ ends; and 23.09% (mouse), 19.61% (human) and 23.19% (chicken) had other nucleotides at their 3′ ends (see Supplementary Table S8). Based on the sequence content comparison, we are positive that these extra nucleotides at 3′ ends are not remnants of untrimmed adapter sequences. In addition, averagely 4.42% (mouse), 10.96% (human) and 11.88% (chicken) of the mature miRNA reads had a mixture of variations: mismatches and/or indels in mapping, 3′ super/sub variants, and non-templated nucleotide addition at 3′ ends. Also interestingly, we found 16, 26 and 0 putative “arm switching” cases in mouse, human and chicken datasets respectively (see Supplementary Table S9). For read cataloging in terms of gene annotation, the counts and percentages of the mapped reads in different genomic features among the total clean reads are shown in Supplementary Table S10. On average, 15.47%, 44.52% and 14.30% of the total clean reads were not aligned to the reference genome in mouse, human and chicken datasets respectively.

We found 144, 174 and 91 novel precursors (see Supplementary Data 1–3) and 144, 175 and 93 novel mature miRNAs (see Supplementary Data 4–6) in the mouse, human and chicken datasets respectively. The predicted RNA secondary structures of novel precursors in dot-bracket notation (DBN)^53^ are shown in Supplementary Data 7–9. On average, 0.09% (mouse), 0.41% (human) and 1.17% (chicken) of the raw reads were mapped to novel precursors perfectly, and 0.042% (mouse), 0.15% (human) and 1.09% (chicken) of the raw reads were finally counted as novel mature miRNA reads after both the position and seed region checks (see Supplementary Table S2).

We used DESeq2^54^ to perform differential expression profile analysis for known and novel mature miRNAs. As shown in Supplementary Table S11, there were 110 known and 12 novel mature miRNAs that showed significant differential expressions (adjusted p-value < 0.05) between two treatments in the mouse dataset; 304 known and 34 novel mature miRNAs displayed significant differential expressions between two treatments in the human dataset; 10, 5 and 1 known and 1, 1, 0 novel mature miRNAs had significant differential expressions respectively in three pairwise comparisons among three treatments in the chicken dataset. We also conducted differential expression profile analysis for miRNA families using DESeq2^54^. For mouse and human datasets, 62 and 163 miRNA families were significantly different respectively between the two treatments (see Supplementary Table S12). For the chicken dataset, 6, 2 and 2 miRNA families were significantly different in three pairwise comparisons among three treatments (see Supplementary Table S12).

### Comparing miRNA data analysis between miRDeep2 and mirPRo

Since miRDeep2 is the most widely used standalone program for miRNA analysis^31^,^36^,^37^,^38^,^39^, we conducted in-depth comparative analysis between miRDeep2 and mirPRo using the aforementioned three datasets. We first performed procedure-by-procedure comparison in which each of the main procedures (*i.e.*, adapter trimming, read mapping and miRNA quantification) was compared separately using the same inputs, and then whole package comparison using comparable parameters for both programs. We also conducted speed performance comparison and found that mirPRo is faster than miRDeep2 (see Supplementary Results).

#### Procedure-by-procedure comparison

##### **Adapter trimming**

The raw sequence reads of mouse and chicken datasets were used as the inputs, because the human dataset was already clean of adapters. In mirPRo, one error base (indel or mismatch) is permitted and the minimum adapter length for trimming is 10 *nt*. In miRDeep2, adapter trimming is based on exact string match, where indel and mismatch are not allowed, and the minimum adapter length for trimming is 1 *nt*. Consequently, on average, 97.62% (mouse) and 51.75% (chicken) of the raw reads were detected with adapters by mirPRo, while 98.26% (mouse) and 64.69% (chicken) by miRDeep2; 91.91% (mouse) and 78.52% (chicken) of the raw reads were kept after trimming adapter (with a length >= 17) in mirPRo, while 92.05% (mouse) and 78.65% (chicken) were kept by miRDeep2 (see Supplementary Table S13). To evaluate which tool is more accurate in adapter trimming, we compared the clean reads generated by these two programs and retrieved the consistent reads (*i.e.*, reads with the same sequence identifiers and sequence contents) and inconsistent reads. The inconsistent reads include: (1) reads with the same identifiers but different sequence contents, and (2) reads kept only by one program after trimming due to the minimum length requirement for the final clean reads (*i.e.*, 17 *nt* for both miRDeep2 and mirPRo). We then mapped inconsistent reads to the pre-miRNA hairpin sequences using Bowtie^55^ with at most two mismatches allowed and compared the sensitivity and true negative rates in mapping. As shown in Supplementary Results and Supplementary Tables S13 and S14, mirPRo exhibits a better performance in trimming adapter sequences in raw reads than miRDeep2.

##### **Mapping** (Novoalign^47^ in mirPRo versus Bowtie^55^ in miRDeep2)

We used the clean collapsed reads generated by miRDeep2 as the same input in both programs for mapping against hairpin sequences, and compared the counts of mapped reads. In mirPRo, we use Novoalign that allows soft clipping, mismatches and indels in mapping. In miRDeep2, read mapping by Bowtie allows at most 2 mismatches, whereas indels are not allowed and soft clips are treated as mismatches. As shown in Supplementary Table S15, on average, 85.10%, 43.40% and 19.76% of the raw reads were mapped successfully for mouse, human and chicken datasets respectively with mirPRo, whereas 81.89%, 39.31% and 18.64% were mapped with miRDeep2. In mirPRo, averagely, 3.54% (mouse), 1.57% (human) and 14.47% (chicken) clean-read-to-hairpin mappings showed mismatches; 0.002% (mouse), 6.30% (human) and 0.003% (chicken) showed insertions; 0.07% (mouse), 1.95% (human) and 0.01% (chicken) showed deletions; 1.19% (mouse), 2.30% (human) and 0.83% (chicken) showed 5′ soft clips; and 16.87% (mouse), 14.55% (human) and 4.87% (chicken) showed 3′ soft clips. In miRDeep2, averagely 20.45% (mouse), 19.45% (human) and 17.25% (chicken) clean-read-to-hairpin mappings had mismatches.

##### **Quantification** (Bowtie as aligner)

We used the collapsed-read-to-hairpin mappings generated by miRDeep2 (Bowtie) and the expression numbers for collapsed reads as the inputs for both programs, and compared the counts of mature miRNA reads generated by the quantification processes of the two programs. As shown in Supplementary Table S16, averagely 76.02% (mouse), 36.34% (human) and 16.19% (chicken) raw reads were counted as mature miRNA reads by mirPRo, whereas 108.52% (mouse), 55.08% (human), and 31.01% (chicken) raw reads were counted as mature miRNA reads by miRDeep2. This result (*e.g.*, 108.52%) clearly demonstrates that a large percentage of the clean reads were over-counted by miRDeep2 in its quantification process.

#### Whole package comparison

**Figure 2:**
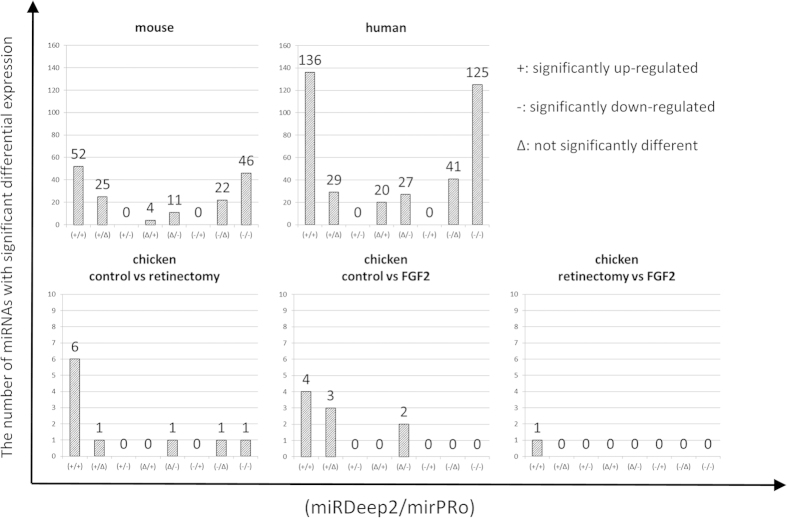
The comparative analyses of mature miRNAs differential expression profiles between miRDeep2 and mirPRo using three datasets. The y-axis represents the number of mature miRNAs. In the x-axis, we have 8 different cases for comparative differential expression analyses of mature miRNAs detected by miRDeep2 and mirPRo. Each case is labeled as (A/B), where A stands for miRDeep2 result and B for mirPRo result, and both A and B can be one of these values: + (up-regulated significantly), - (down-regulated significantly) and Δ (no significant differential expression). For example, the case (+/+) means that a mature miRNAs is significantly up-regulated, reported by both miRDeep2 and mirPRo.

We conducted a whole-package comparative analysis of mirPRo and miRDeep2 independently. In miRDeep2, some of entries in final results had duplicate names of mature miRNAs and their pre-miRNAs, with different sets of mature miRNA counts across different libraries (see mouse and human data, Supplementary Table S17). These discrepancies made users unable to tell which one was correct, causing difficulty in further downstream statistical analysis. In order to perform differential analysis using DESeq2, we added unique IDs (*i.e.*, the combination of a numeric prefix, mature miRNA name and pre-miRNA name) for each entry. DESeq2 analysis showed that in miRDeep2, 145 and 332 mature miRNAs had significant differential expressions in mouse and human datasets respectively, and 9, 8 and 1 mature miRNAs were significantly different in 3 pairwise comparisons among three treatments in chicken dataset (see Supplementary Table S18). In contrast, DESeq2 analysis for mirPRo results showed that 113 and 308 mature miRNAs had significant differential expressions in mouse and human datasets respectively, and 8, 6 and 1 mature miRNAs had significant differential expressions in 3 pairwise comparisons among three treatments in the chicken dataset (see Supplementary Table S19). As shown in Fig. 2, both miRDeep2 and mirPRo have reported most of the same miRNAs that have significant differential expressions. However, these two programs have different calls for some differentially expressed miRNAs (also see Supplementary Table S20). It is worthy of note that, as shown in Fig. 2, no mature miRNA shows opposite differential expression results between miRDeep2 and mirPRo (*i.e.*, the case (+/−) means up-regulated in miRDeep2 and down-regulated in mirPRo, and vice versa for the case (−/+)). However, there are more miRNAs reported as differentially expressed by miRDeep2 (*i.e.*, cases (+/Δ) and case (−/Δ)) than by mirPRo (*i.e.*, cases (Δ/+) and case (Δ/−)). For those miRNAs with inconsistent calls, we further scrutinized their alignment results. For instance, mirPRo reported that mmu-miR-152-3p is down-regulated significantly (see mouse data alignments in Supplementary Data 10) whereas miRDeep2 called it a miRNA without significant differential expression. Furthermore, mirPRo reported that hsa-miR-324-5p is up-regulated significantly (see human data alignments in Supplementary Data 11) whereas miRDeep2 determined that it does not have a significant differential expression. mmu-miR-152-3p exemplifies the case (Δ/−) while has-miR-324-5p exemplifies the case (Δ/+) shown in Fig. 2. The alignment results from miRDeep2 are presented in Supplementary Data 12–21. Clearly, we found that the mappings in mirPRo were more accurate (allowing indels and soft clips) than miRDeep2, and mirPRo was more stringent in seed region check (no error base allowed) than miRDeep2. Consequently, mirPRo shows less false positives in calling significantly differentially expressed miRNAs than miRDeep2.

In miRDeep2, there were no consistent, unified names for novel precursors and their mature miRNAs in all libraries (*i.e.*, the same novel precursor might have different names in different libraries). This makes it difficult for users to perform downstream differential analysis. In contrast, in mirPRo, we unified the names of both novel precursor and their mature miRNAs across all libraries by the following criteria: (1) novel precursors with the same genomic coordinates in different libraries are considered the same novel precursors, and their names include a unique identifier “XXX-novel-mir-YYY” (“XXX” represents the species name and “YYY” is a unique number). In the FASTA output of novel precursors, there is a description field that defines their genome locations (chromosome, strand, start and end position) (see Supplementary Data 1–3); (2) novel mature miRNAs with the same precursors and identical sequences across different libraries are considered to be the same mature miRNAs, and their names include a unique identifier “XXX-novel-miR-YYY” (“XXX” represents the species name and “YYY” is the same number as its precursor). In the FASTA output of novel mature miRNAs, there is a description field that contains the name of a known mature miRNAs from the reference species, whose seed region is the exact same as the reported novel miRNA (see Supplementary Data 4–6). mirPRo provides novel pre-miRNA sequences in DBN^53^ format in order to display their secondary structures (see Supplementary Data 7–9), which are not available in miRDeep2.

## Discussion

IsomiRs are mainly generated due to imprecise cleavage of pre-miRNAs, RNA editing and non-templated nucleotide additions at 3′ end of miRNAs^1^,^15^,^18^. IsomiRs account for a large percentage of total mature miRNAs in cells and are reported to be functionally relevant along with canonical miRNAs^56^. As another form of miRNA variation, “arm switching” in miRNA biogenesis is related with differential miRNA target interaction in gene expression regulation^3^,^57^,^58^,^59^.

Different from miRDeep2, mirPRo allows close examination of miRNA variations, including isomiRs and “arm switching” (see Fig. 1). The improvements in adapter trimming, read mapping and mature miRNA quantification approaches in mirPRo allow for more reads mapped to pre-miRNAs (hairpins) than miRDeep2, because mirPRo tolerates error bases (mismatch and indel) in adapter trimming and permits error bases and soft clips in read mapping. Our comparative data analysis suggests that a significant portion of final clean reads showing miRNA variations were ignored by miRDeep2, because it does not allow indels or soft clips in mapping.

We have found a large percent (60.21% mouse, 52.07% human and 58.83% chicken) of the mature miRNA reads different from their canonical mature miRNAs in alignments. Most of the differences are presented as nucleotide shifts in the 3′ end, which might be caused by imprecise precursor terminal cutting by enzymes in miRNA biogenesis^18^. Previous studies have shown that some mature miRNAs have A (adenine) to I (inosine) modification in the internal sequence due to RNA editing, which presents mainly as A to G mismatch in mapping^52^. We found that in all mature miRNA reads with mismatches in mappings, a large percent (5.67% mouse, 13.17% human and 27.08% chicken) had purely A to G mismatches, consistent with previous studies. We detected the non-templated nucleotide addition in the 3′ end of mature miRNA reads such as adenine/uracil nucleotides or their homopolymer tails, consistent with the previous findings^60^. Interestingly, we also detected lots of sequence patterns of 3′-end non-templated nucleotide additions other than poly(A)/(U) tails, and they are clearly not adapter remnants. On the other hand, we found some reads that have 5′-end soft clips in mapping. However, these reads were filtered out in our data analyses after seed region check. These reads might be the results of potential 5′-end non-templated nucleotide addition or sequencing errors, and are worthy of further evaluation in the future. Moreover, mirPRo can detect the potential “arm switching” cases and allow users to further study the mature miRNAs with “arm switching” under different treatments. These functions can clearly deepen our understanding of miRNA variations and their relevant regulatory mechanisms.

Our comparative data analysis shows that miRDeep2 has a problem of over-counting in quantifying known miRNAs, making the counts of some mature miRNA reads unreliable. In miRDeep2 output, the counts of some mature miRNAs are confusing because of duplicate name entries for both precursor and mature miRNAs. The over-counting problem in miRDeep2 is due to the fact that when a collapsed read has non-unique mappings to hairpins, miRDeep2 will add the read count to the corresponding mature miRNAs independently without dividing it evenly. Evidently, this will result in an increment in read counts for the mature miRNAs with non-unique mapping reads. In contrast, mirPRo avoids this problem by a commonly used read count dividing method^61^,^62^ based on the numbers of mapped locations in reference sequences (see **Methods**).

Different from other popular tools, mirPRo provides the optional function of seed region check in miRNA quantification. For animals, the seed regions determine the functions of mature miRNAs^2^, and this function makes sure that all mature miRNA reads have the same seed region as the corresponding canonical mature miRNAs. When performing analysis without the seed region check using mirPRo, more reads will be counted and more variations in the 5′ end of the mature miRNAs could be detected for further exploration.

Moreover, mirPRo offers some new functions not available in other popular tools (see Supplementary Table S1). In miRBase, pre-miRNA family clustering was done by using the single-linkage method to cluster the pre-miRNA sequences based on BLAST hits and then manually adjusting the clustered families by multiple sequence alignments, keeping in mind that the clustered miRNAs in a family possibly have the same ancestor^51^,^63^. pre-miRNAs from the same family always have the same consensus secondary structures and often conduct similar functions^64^. Using miRNA family classification data obtained from miRBase^51^, mirPRo can perform miRNA family expression quantification and allow users to study differential expressions of miRNA families in their data. In mirPRo, cataloging all clean reads in terms of genome annotation can provide users overall assessment of sequencing and alignment quality. Also, mirPRo unifies the names of novel miRNAs and provides a count table of novel mature miRNAs in all samples.

In conclusion, mirPRo can quantify both known and novel miRNAs more accurately and provide detailed data for users to explore miRNA variations. Without a doubt, mirPRo is a valuable addition to the research community in processing large-scale miRNA sequencing data.

## Methods

### Data collection

The first miRNA dataset is from mouse synovial fibroblast with two treatments^45^: human tumor necrosis factor transgenic (Tg) group and control wild type (WT) group, where each treatment has two biological replicates. The second miRNA dataset is from human induced pluripotent stem cell-derived cardiomyocyte (hiPSC) with two treatments^46^: endothelin 1 (ET1) stimulated group and control group, where each treatment has three biological replicates. The third dataset is our own chicken miRNA data from retinal pigmented epithelium (RPE) and includes three treatments: E4 development RPE (control), RPE collected at 6 hrs post-retinectomy (retinetomy) and RPE collected at 6 hrs post-retinectomy in the presence of FGF2 (FGF2), where each treatment consists of three biological replicates. Similar treatments have been reported during RPE reprogramming^65^.

The raw mouse miRNA sequencing data was downloaded from http://www.ncbi.nlm.nih.gov/geo/query/acc.cgi?acc=GSE31667 with the sequencing adapter “ATCTCGTATGCCGTCTTCTGCTTG”. *Mus musculus* genome sequences in FASTA format and genome annotation in GTF format (Ensembl release 79: GRCm38.p3; top level assembly) were downloaded from http://www.ensembl.org/. The raw human miRNA sequencing data was downloaded from http://www.ncbi.nlm.nih.gov/geo/query/acc.cgi?acc=GSE60292, which was clean of adapter sequences. *Homo sapiens* genome sequences and genome annotation (Ensembl release 79: GRCh38.p2; primary assembly) were downloaded from Ensembl website. For human genome, the top level assembly is too large (~40 GB) to build a Novoalign index file, so we used its primary assembly instead of its top level assembly. The adapter for our chicken data is “TGGAATTCTCGGGTGCCAAGG”, and *Gallus gallus* genome sequences and genome annotation (Ensembl release 79: Galgal4; top level assembly) were downloaded from Ensembl website. The raw data in SRA format was transformed to FASTQ format by SRA Toolkit version 2.5.2 (http://www.ncbi.nlm.nih.gov/Traces/sra/sra.cgi?cmd=show&f=software&m=software&s=software). The sequences for both mature canonical miRNAs and their hairpins, as well as the precursor miRNA family classification data (*i.e.*, “miFam.dat”), were downloaded from miRBase^51^ (release 21).

### miRNA data analysis by mirPRo

The prerequisite tools for mirPRo include: (1) FASTX-Toolkit (http://hannonlab.cshl.edu/fastx_toolkit/index.html, version 0.0.14); (2) Novoalign^47^ (http://www.novocraft.com/support/download/, release V3.02.11); (3) HTSeq^48^ (http://www-huber.embl.de/users/anders/HTSeq/doc/install.html, version 0.6.1); (4) RNAfold^50^ (http://www.tbi.univie.ac.at/RNA/index.html, version 2.1.9); (5) randfold^49^ (http://bioinformatics.psb.ugent.be/supplementary_data/erbon/nov2003/, version 2). They need to be installed before running mirPRo.

mirPRo first extracted mature and precursor miRNA sequences annotated by miRBase^51^ for a given species (*i.e.*, mouse, human or chicken) and mapped these mature miRNAs to their hairpins using Novoalign, allowing only perfect mappings to obtain the accurate positions of mature miRNAs in corresponding hairpins for downstream analysis.

The raw read quality filtering was performed by FASTX-Toolkit (*fastq_quality_filter*, http://hannonlab.cshl.edu/fastx_toolkit/index.html) to filter out reads with poor qualities using the following settings: (1) the minimum quality score for each base = 20; (2) the percent of bases that must have the minimum quality score ≤95%. The adapter sequence was trimmed off by subprogram *mirpro_findAdapter* of mirPRo, with the following settings: (1) the maximum number of error bases including mismatches and indels in detected adapter sequences = 1; (2) the minimum length of detectable adapters = 10; (3) the minimum length of final clean reads without adapters = 15 (the minimum length of mature miRNAs in miRBase is 15). The clean reads were then collapsed by the program *fastx_collapser* in FASTX-Toolkit with expression numbers counted (*i.e.*, the count of reads that support one collapsed read).

The collapsed reads were then mapped to the known pre-miRNAs (hairpin sequences) using Novoalign with the following settings: (1) mismatch penalty = 30; (2) gap opening penalty = 40; (3) gap extension penalty = 6; (4) the maximum penalty score for alignment = 60; (5) soft clips are allowed; (6) for the reads that can be mapped to more than one hairpin, and the best mappings with the lowest penalty score are kept. We used the option “-r All –R o” to report the mapping(s) with the best alignment score for each read. The maximum penalty score for alignment (60) allowed at most 2 mismatches or 3 indels in one opening gap in mappings.

Using the results of mature-miRNA-to-hairpin and collapsed-read-to-hairpin mappings, we performed known miRNA quantification with the subprogram *mirpro_quantifier* with the following settings: (1) the maximum number of nucleotide shift in the upstream or downstream of the 5′/3′ end of a mature miRNA read is <= 3 in reference to its canonical mature miRNA annotated by miRBase (*i.e.*, the position check); (2) remove the reads that don’t have the exact same seed regions (2^nd^–8^th^ *nt*) as their canonical mature miRNAs (*i.e.*, the seed region check). The detailed quantification algorithm was as follows: (1) use mature-miRNA-to-hairpin mappings to record the positions of canonical mature miRNAs in corresponding hairpins, and the recorded mature miRNA and hairpin pairs should share the same IDs (*e.g.*, mature: mmu-miR-YYY-5p or mmu-miR-YYY-3p; hairpin: mmu-mir-YYY, where YYY represents a number); (2) filter out the reads that did not pass either position or seed region check; (3) consider the remaining read mappings as qualified mappings, and for each collapsed read, if it has unique mapping, add the read count directly to the corresponding mature miRNA; (4) if the collapsed read has non-unique mappings, record the numbers of soft clips in these mappings respectively (*N*_*1*_, *N*_*2*_, … *N*_*n*_), and then find the minimum number of soft clips (*N*_*min*_) and its corresponding mapping (s), which is (are) considered as the best priority mappings; (6) if the number of the best priority mapping is equal to 1, add the read count directly to the corresponding mature miRNA; if the number of the best priority mappings is larger than 1, divide the read count evenly by the number of all best priority mappings and then add it to the counts of corresponding mature miRNAs. Finally the counts of known miRNAs in all libraries were rounded to integers and output to one csv file.

For miRNA family expression quantification, we relied on the miRNA family information provided by miRBase, and clustered the mature miRNAs into different miRNA families with the summation of relevant mature miRNA read counts. Mature miRNAs that do not belong to any known family in miRBase were considered to have their own individual families.

For detecting miRNA isoforms - isomiRs, we calculated the counts of the mature miRNA reads that had mismatches, indels, 5′/3′ nucleotide shift or soft clips in mappings, and further classified the 3′-soft clips as adenine fragment (one or more A, or poly (A) tail), uracil fragment (one or more U, or poly (U) tail) or other patterns. We further listed all the sequences of other patterns and compared them with the adapter sequences to make sure that they are not adapter remnants or fragments.

To detect “arm switching”, we used the subprogram *mirpro_armSwitch* of mirPRo, focusing on the mature miRNAs that have two different forms (5p and 3p) from the same hairpins, and compared the counts of two forms in each library to determine which form was consistently dominant. We adopted the following criteria: (1) the count fold-change between two forms >2; (2) the count difference between two forms >10. If the counts of the two forms do not satisfy these criteria, no form is dominant. We further detected the “arm switching” cases by the following algorithm: (1) in each treatment, we selected the mature miRNAs that have the same dominant forms in all replicates of one treatment; (2) we compared the dominant forms of each selected miRNA across different treatments, and if there was an inconsistency, then the selected miRNAs would be considered to have potential “arm switching” cases among different treatments.

For cataloging clean reads in terms of genome annotation, mirPRo mapped final clean sequence reads to the reference genome sequences by Novoalign with the maximum penalty score of alignment = 60. Then, it categorized and counted the mapped clean reads in different features annotated in gene annotation GTF files by the subprogram *htseq-count* of HTSeq. As the parameters for HTSeq, the “gene_id” is set as the feature ID by default while the minimum alignment quality value was set to 0.

For novel miRNA prediction, we used a similar prediction algorithm as in miRDeep2 with our improvements. (1) We mapped collapsed reads to the genome and only kept the perfect mappings with mapped read lengths between 18 and 25 *nt* inclusively and mapped locus/loci <= 5. (2) We used the remaining mappings to excise potential precursors (hairpins) from the genome by miRDeep2 algorithm. (3) We mapped collapsed reads and known mature miRNAs to the excised hairpins, and kept the perfect mappings with mapped read lengths between 18 and 25 *nt* as signatures. Different from miRDeep2 that allows 1 mismatch here, we only permitted perfect mapping to reduce false positives. (4) We used RNAfold^50^ to calculate the structure and minimum free energy of the excised hairpins. (5) For each excised hairpin, we selected the sequence of the mapped reads with the highest read stack as the mature miRNA sequence of the hairpin. Here, we required that the fold-change was larger than 2 in comparison with the second highest read stack; if no mapped read satisfied this fold-change criterion, the excised hairpin would be discarded. The fold-change requirement is our improvement over miRDeep2 to reduce false positives. (6) We selected all excised hairpins by miRDeep2 algorithm and computed their randfold p-values by randfold^49^. (7) We calculated miRDeep2 scores of all excised hairpins by miRDeep2 algorithm, using the results of (3), (4), (5) and (6). (8) We performed controls and surveys by miRDeep2 algorithm. (9) We selected the excised hairpins with miRDeep2 score >0 as novel pre-miRNAs and the corresponding mature miRNA part in the hairpins as novel mature miRNAs.

After the novel miRNA prediction, mirPRo generated the lists of novel mature and precursor miRNAs for each library and removed the redundancy of the sequences. For novel mature miRNA, a sequence file in FASTA format was generated. For novel precursors, sequence files in FASTA and DBN format were created. The clean collapsed reads were then mapped to novel precursors only allowing perfect mappings, and novel miRNA quantification was performed with the same settings as known miRNAs. Finally, the output counts of novel mature miRNAs in all libraries were rounded to integers in a single csv file.

### miRNA data analysis by miRDeep2

In miRDeep2 (version 2.0.0.5), the *mapper* and *quantifier* modules were used in analyzing known miRNAs in the data whereas the *miRDeep2* module was used for novel miRNA prediction. The program settings were as follows: (1) the minimum clean read length = 17 after adapter trimming (for novel miRNA prediction, miRDeep2 needs input clean read length >= 17); (2) maximum mismatch bases allowed in read mapping is 2 (indels are not allowed in miRDeep2); (3) because miRDeep2 needs a reference species for its novel miRNA prediction, rat, mouse and zebra finch were selected as the reference species for mouse, human and chicken respectively; (4) other settings were default parameters used by miRDeep2. miRDeep2 require Bowtie for read-to-hairpin mapping (quantifying known miRNAs) and read-to-genome mapping (novel miRNA prediction), and we have used Bowtie (version 0.12.7) in our comparative data analysis.

### Statistical analysis

The R (version 3.2.1) package DESeq2^54^ (version 1.8.1) was used to detect the differential expression of the mature miRNAs and miRNA families between different treatments for all three datasets. The counts of mature and novel miRNAs in all samples are used as a single input of DESeq2. The size factor was estimated by the median-of-ratios method used in DESeq^66^. After normalization, the different expression was tested based on a Negative Binomial distribution^54^. Adjusted p-valve < 0.05 was considered to be statistically significant.

## Additional Information

**How to cite this article**: Shi, J. *et al.* mirPRo – a novel standalone program for differential expression and variation analysis of miRNAs. *Sci. Rep.* **5**, 14617; doi: 10.1038/srep14617 (2015).

## Supplementary Material

Supplementary Information

Supplementary Table S1

Supplementary Table S2

Supplementary Table S3

Supplementary Table S4

Supplementary Table S5

Supplementary Table S6

Supplementary Table S7

Supplementary Table S8

Supplementary Table S9

Supplementary Table S10

Supplementary Table S11

Supplementary Table S12

Supplementary Table S13

Supplementary Table S14

Supplementary Table S15

Supplementary Table S16

Supplementary Table S17

Supplementary Table S18

Supplementary Table S19

Supplementary Table S20

Supplementary Data 1-9

Supplementary Data 10

Supplementary Data 11

Supplementary Data 12-21
