# Supplementary material for: mirPRo–a novel standalone program for differential expression and variation analysis of miRNAs
Source: Sci Rep. 2015 Oct 5;5:14617. doi: 10.1038/srep14617 (PMC4592965; doi:10.1038/srep14617)
Supplement: Supplementary Information [file srep14617-s1.pdf]

Supplementary Information

for

mirPRo – a novel standalone program for differential expression and variation analysis of miRNAs

Jieming Shi[1], Dong Min[1], Lei Li[1], Lin Liu[1], Agustin Luz Madrigal[1, 2], Katia Del Rio-Tsonis[1] Panagiotis A. Tsonis[2] and Chun Liang[1, 3, +]

Contents

1. Supplementary Results …………………......…………………………………………............. 2

2. Supplementary Figures …………………………………………………............................. 4

3. Supplementary Table titles and legends …………………………………...…..………… 5

4. Supplementary Data titles and legends …………………………………...........……….. 7

1. Supplementary Results

Adapter trimming comparison between mirPRo and miRDeep2

To evaluate which tool is more accurate in adapter trimming, we compared the clean reads generated by these two tools and extracted the consistent reads (i.e., reads with the same sequence identifiers and sequence contents) and inconsistent reads. The inconsistent reads are composed of two components: (1) reads with the same identifiers but different sequence contents due to differential trimming results from the two programs, and (2) reads kept only by one program after trimming due to the minimum length requirement for the final clean reads. For both mirPRo and miRDeep2, the minimum length requirement is 17 nt. Then, we mapped all inconsistent reads to the pre-miRNA hairpin sequences using Bowtie with at most two mismatches allowed, and calculated the sensitivity and true negative rates in mapping (see Supplementary Table S14 for detailed information about equations we used).

For the inconsistent reads, our overall assumption is that a raw read will be a positive read when it can be mapped to pre-miRNA after adapter trimming by either or both of the two programs, thus no false positive, and it will be a negative read if it cannot be mapped to pre-miRNA after adapter trimming by both programs. For a raw sequence in the inconsistent set, there are 4 possible cases that allow us to calculate specificity and sensitivity measures for both programs (see Supplementary Table S14 for details): (1) if the clean sequence in both program **A** (e.g., mirPRo) and **B** (e.g., miRDeep2) can be mapped to pre-miRNA, this read is a true positive case for both program **A** and **B**; (2) if the clean sequence in program **A** can be mapped, but not mapped or not kept in program **B**, this read is a true positive case for program **A** whereas it is a false negative for program **B**; (3) if the clean sequence in program **B** can be mapped, but not mapped or not kept in program **A**, this read is a true positive case for program **B** whereas it is a false negative case for program **A**; (4) if the clean sequence in both program **A** and **B** cannot be mapped or kept, this read is a true negative for both program **A** and **B**.

Because of our assumption that there is no false positive in both programs, the specificity is the same (i.e., 100%) for both program. On average, for the inconsistent reads, the sensitivity is 86.46% (mouse) and 87.37% (chicken) and the true negative rate is 96.41% (mouse) and 99.45% (chicken) in mirPRo. In contrast, the sensitivity is 19.65% (mouse) and 66.19% (chicken) and the true negative rate is 82.33% (mouse) and 98.54% (chicken) in miRDeep2 (see Supplementary Table S13). Clearly, this demonstrates that mirPRo exhibits better performance than miRDeep2 in cleaning and trimming adapter sequences from raw sequence reads.

Speed performance comparison between mirPRo and miRDeep2

We used the same FASTQ file (mouse data: sample "SRR333597", ~3GB) as input, and set similar parameters for both miRDeep2 and mirPRo to compare the running speeds of two programs in a Ubuntu Linux desktop computer (8 processors, Intel(R) Core(TM) i7-2600 CPU @ 3.40GHz; 16 GB RAM) and a Ubuntu Linux server computer (64 processors, Intel(R), Xeon(R), X7560 CPU @ 2.27GHz; 256 GB RAM). We used previously built mouse genome indexes for Bowtie and Novoalign, and ran through all main analysis procedures, including adapter trimming, mapping, quantification and novel miRNA prediction.

In mirPRo, we used the following parameters in the main program “mirpro”: “--novel 1 -q 0 --clean-len 17 -v 0 --arm 0”, which means that (1) novel miRNA prediction is enabled; (2) read quality filtering is disabled (miRDeep2 doesn’t have this function); (3) the minimum length requirement for a final clean read after adapter trimming is 17 *nt*; (4) IsomiR detection and “arm switching” detection are disabled (miRDeep2 doesn’t have these functions); other parameters in mirPRo are in default settings (see the readme file in https://sourceforge.net/projects/mirpro/files/). In miRDeep2, we (1) set “-l 17” in the “mapper” module, which means that the minimum length requirement for a final clear read after adapter trimming is 17 *nt*; (2) set “-g 2” in the “quantifier” module, which means that at most 2 mismatches are allowed in clean read mapping against precursor miRNA sequences; (3) used “miRDeep2” module to predict novel miRNAs with default settings. Our comparison shows that the run time has little difference between two different machines (i.e., a desktop computer and a computer server, and a single processor has been utilized in both computers), and mirPRo (~1.5 hrs) is faster than miRDeep2 (~2.75 hrs), even though we allow indels in adapter trimming and mapping reads to precursor miRNAs.

2. Supplementary Figures

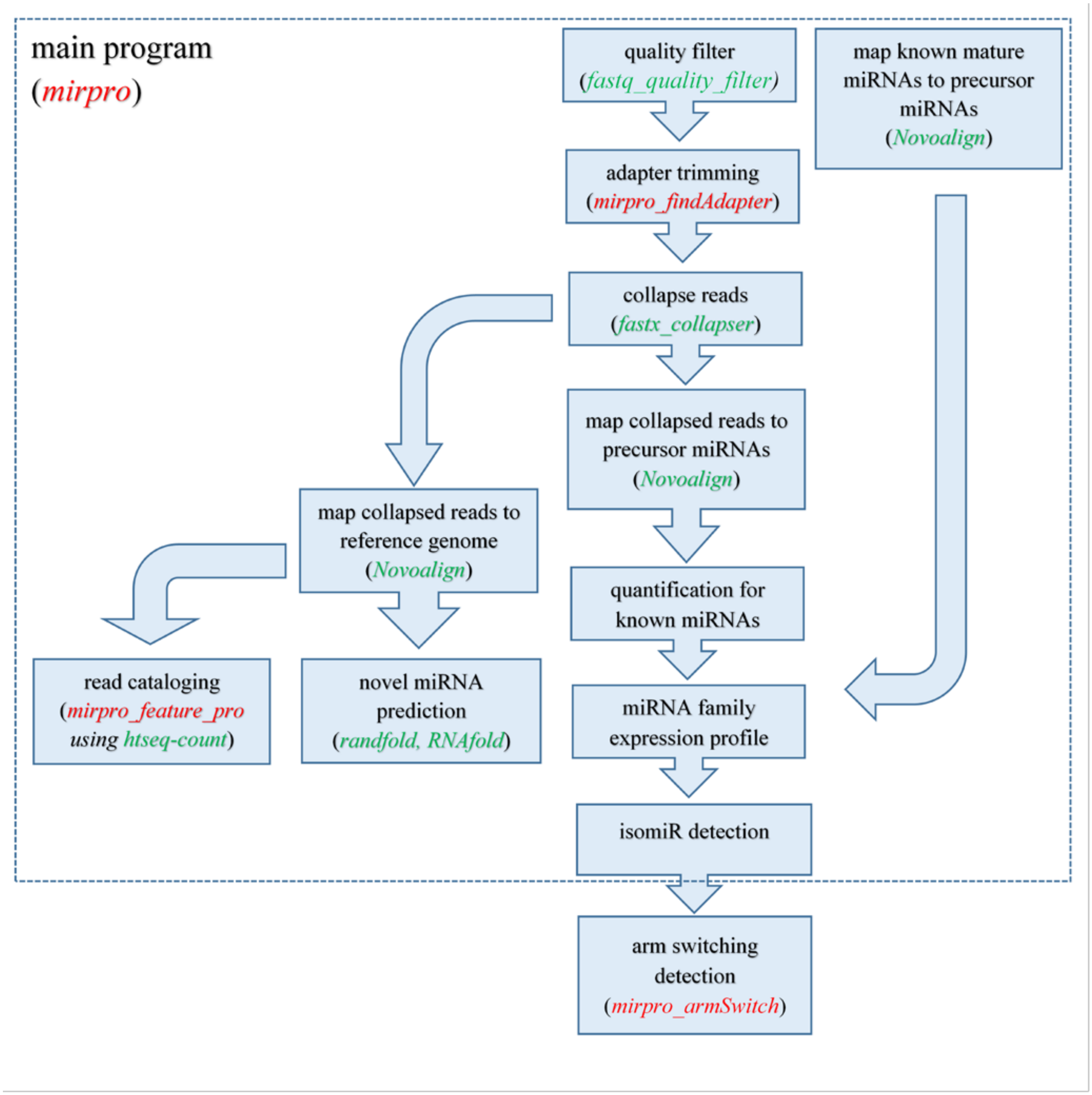

Supplementary Figure 1: The workflow of mirPRo.

Implemented in C++, the whole mirPRo package is composed of a main program (*mirpro*) and several component programs (e.g., *mirpro_findAdapter*, *mirpro_feature_pro* and *mirpro_armSwitch*). A few third-party tools highlighted in green color will be invoked by either the main or component programs. Users can initiate the main program to finish most functions, except "arm switching detection", which can be called independently after the main program is done with execution. Some of the component programs (e.g. *mirpro_findAdapter*) can be used independently. As shown in (https://sourceforge.net/projects/mirpro/files/?source=navbar), users can fine tune many parameters of both the main and component programs, if they do not want the default parameters.

3. Supplementary table titles and legends

Supplementary Table S1: Comparisons in major features and functions between mirPRo and other popular tools.

Supplementary Table S2: The individual read-based statistics of miRNA analysis in mouse, human and chicken datasets using mirPRo.

In mouse dataset, WT group includes sample SRR333597 and SRR333598, and Tg group includes sample SRR333599 and SRR333600; In human dataset, control group includes sample SRR1542714, SRR1542716 and SRR1542718 and ET1 group includes sample SRR1542715, SRR1542717 and SRR1542719; In chicken dataset, control group includes sample 1, 2 and 3, retinectomy group includes sample 4, 5 and 6, and FGF2 group includes sample 7, 8 and 9.

Supplementary Table S3: The collapsed read-based statistics of miRNA analysis in mouse, human and chicken datasets using mirPRo.

Supplementary Table S4: The unique and non-unique mappings of final clean reads to pre-miRNA hairpins in mouse, human and chicken datasets using mirPRo.

Supplementary Table S5: The quantification results of known mature miRNAs in mouse, human and chicken datasets using mirPRo.

Supplementary Table S6: The overall statistics of detected isomiRs of known mature miRNAs in mouse, human and chicken datasets using mirPRo.

“5' super (+*n*) mature miRNA variants” means that the read has *n* (*n*=1, 2, 3) nucleotides upstream shift in 5’ end in mapping to hairpins compared with the canonical mature miRNA sequences. “5' sub (-*n*) mature miRNA variants” means that the read has *n* (*n*=1, 2, 3) nucleotides downstream shift in 5’ end in mapping to hairpins compared with the canonical mature miRNA sequences. “3' super (+*n*) mature miRNA variants” means that the read has *n* (*n*=1, 2, 3) nucleotides downstream shift in 3’ end in mapping to hairpins compared with the canonical mature miRNA sequences. “3' sub (+*n*) mature miRNA variants” means that the read has *n* (*n*=1, 2, 3) nucleotides upstream shift in3’ end in mapping to hairpins compared with the canonical mature miRNA sequences.

Supplementary Table S7: The detailed statistics of detected isomiRs for each known mature miRNA in mouse, human and chicken datasets using mirPRo.

Supplementary Table S8: The detected 3’-end non-templated nucleotide addition of mature miRNA reads in mouse, human and chicken datasets using mirPRo.

Supplementary Table S9: The putative cases of “arm switching” of miRNAs detected among different treatments of mouse, human and chicken datasets using mirPRo.

“NA” means that there is no dominant form detected in each biological replicate of the treatment. “5p” means that the 5p mature miRNA is the dominant form in each biological replicate of the treatment. “3p” means that the 3p mature miRNA is the dominant form in each biological replicate of the treatment.

Supplementary Table S10: The read cataloging results based on genome annotation in mouse, human and chicken datasets using mirPRo.

The excel worksheet labeled "mouse" represents the counts of the final clean reads mapped to different features of genome annotation for mouse, while the worksheet labeled "mouse %" represents the percentages of final clean reads mapped to different features of genome annotation for mouse among total clean reads. This same rule applies for human and chicken datasets. In each sheet, "*__alignment_not_unique*" represents the reads with more than one reported alignment; "*__ambiguous*" represents the reads which can have been assigned to more than one features and are not counted for any of these; "*__no_ feature*" represents the reads which could not be assigned to any feature; "*__not_aligned*" represents the reads without valid alignment.

Supplementary Table S11: The differential expression analysis of both known and novel mature miRNAs between different treatments for mouse, human and chicken datasets using mirPRo and DESeq2.

"baseMean": mean of normalized counts for all samples in two treatments. "lfcSE": standard error. "stat": Wald statistic. "pvalue": Wald test p-value. "padj": BH adjusted p-values.

Supplementary Table S12: The differential expression analysis for known miRNA families between different treatments for mouse, human and chicken datasets using mirPRo and DESeq2.

Supplementary Table S13: The statistics of adapter trimming comparison between mirPRo and miRDeep2 in mouse and chicken datasets.

Supplementary Table S14: Different cases related to the calculation of specificity and sensitivity measures between two different programs used for adapter trimming.

Supplementary Table S15: The comparison of read mapping between mirPRo and miRDeep2 in mouse, human and chicken datasets.

Supplementary Table S16: The comparison of known miRNA quantification between mirPRo and miRDeep2 in mouse, human and chicken datasets.

Supplementary Table S17: The entries in miRDeep2 output that share the same mature and hairpin miRNA names in mouse and human datasets.

In the mouse data, mature miRNA "mmu-miR-466i-5p" has two entries with the same mature and hairpin miRNA names, but with different counts of mature miRNAs.

Supplementary Table S18: The differential expression analysis of known mature miRNAs between different treatments in mouse, human and chicken datasets using miRDeep2 and DESeq2.

Supplementary Table S19: The differential expression analysis of known mature miRNAs between different treatments in mouse, human and chicken datasets using mirPRo and DESeq2.

Supplementary Table S20: The mature miRNAs with different results in their differential expression analysis between miRDeep2 and mirPRo in mouse, human and chicken datasets.

4. Supplementary Data titles and legends

Supplementary Data 1: Novel precursor miRNA sequences detected in the mouse dataset using mirPRo.

The name of the novel precursor miRNA is “XXX-novel-miR-YYY”, where “XXX” represents the species name and “YYY” is a unique number. The description includes the genome locations in the format "chromosome:strand:start:end".

Supplementary Data 2: Novel precursor miRNA sequences detected in the human dataset using mirPRo.

Supplementary Data 3: Novel precursor miRNA sequences detected in the chicken dataset using mirPRo.

Supplementary Data 4: Novel mature miRNA sequences detected in the mouse datasets using mirPRo.

The name of the mature miRNA is “XXX-novel-miR-YYY” (“XXX” represents the species name and “YYY” is the same number in its precursor). The description includes the name of the mature miRNA in the reference species that has the same seed region in the format "seed:XXX-miR-YYY". "seed:no" means that there is no such mature miRNA in the reference species.

Supplementary Data 5: Novel mature miRNA sequences detected in the human dataset using mirPRo.

Supplementary Data 6: Novel mature miRNA sequences detected in the chicken datasets using mirPRo.

Supplementary Data 7: Novel precursor miRNA structures in DBT format for the mouse dataset.

Supplementary Data 8: Novel precursor miRNA structures in DBT format for the human dataset.

Supplementary Data 9: Novel precursor miRNA structures in DBT format for the chicken dataset.

Supplementary Data 10: The collapsed-read-to-hairpin alignments of mmu-mir-152 in mirPRo.

mirPRo reported mmu-miR-152-3p down-regulated significantly whereas miRDeep2 reported no significant differential expression. This exemplifies the case (Δ/-) in Figure 2.

Here we present the alignments of the final clean collapsed reads that can be mapped to the selected precursors in all samples for mouse and human data. In each sheet, the column "M/I/D/N" represents the number of "mismatches/insertions/deletions/nucleotide N" in the alignment. In the column “sequence”, the lower case in read sequences means matched nucleotide; the upper case in the middle of read sequences means error base (mismatch, insertion, or deletion (D)); the upper case in the 5’ or 3’ end means soft clip.

Supplementary Data 11: The collapsed-read-to-hairpin alignments has-mir-324 in mirPRo.

mirPRo reported miR-324-5p up-regulated significantly whereas miRDeep2 reported no significant differential expression. This exemplifies the case (Δ/+) in Figure 2.

Supplementary Data 12: The collapsed-read-to-hairpin alignments of mmu-mir-152 in sample "SRR333597" in miRDeep2.

mirPRo reported miR-152-3p down-regulated significantly whereas miRDeep2 reported no significant differential expression. This exemplifies the case (Δ/-) in Figure 2.

The PDF file is generated by miRDeep2; the column "reads" represents the read counts; the column "mm" represents the number of mismatches in alignment; the lower case in read sequences means matched nucleotide; the upper case in read sequences means mismatch.

Supplementary Data 13: The collapsed-read-to-hairpin alignments of mmu-mir-152 in sample "SRR333598" in miRDeep2.

Supplementary Data 14: The collapsed-read-to-hairpin alignments of mmu-mir-152 in sample "SRR333599" in miRDeep2.

Supplementary Data 15: The collapsed-read-to-hairpin alignments of mmu-mir-152 in sample "SRR333600" in miRDeep2.

Supplementary Data 16: The collapsed-read-to-hairpin alignments of hsa-mir-324 in sample "SRR1542714" in miRDeep2.

mirPRo reported miR-324-5p up-regulated significantly whereas miRDeep2 reported no significant differential expression. This exemplifies the case (Δ/+) in Figure 2.

Supplementary Data 17: The collapsed-read-to-hairpin alignments of hsa-mir-324 in sample "SRR1542715" in miRDeep2.

Supplementary Data 18: The collapsed-read-to-hairpin alignments of hsa-mir-324 in sample "SRR1542716" in miRDeep2.

Supplementary Data 19: The collapsed-read-to-hairpin alignments of hsa-mir-324 in sample "SRR1542717" in miRDeep2.

Supplementary Data 20: The collapsed-read-to-hairpin alignments of hsa-mir-324 in sample "SRR1542718" in miRDeep2.

Supplementary Data 21: The collapsed-read-to-hairpin alignments of hsa-mir-324 in sample "SRR1542719" in miRDeep2.
