# Supplementary material for: mirPRo–a novel standalone program for differential expression and variation analysis of miRNAs
Source: Sci Rep. 2015 Oct 5;5:14617. doi: 10.1038/srep14617 (PMC4592965; doi:10.1038/srep14617)
Supplement: Supplementary Data 12-21 [file srep14617-s25.zip › Supplementary Data 12.pdf]

miRBase precursor : mmu-mir-152
Total read count : 227830
mmu-miR-152-5p read count : 535
mmu-miR-152-3p read count : 227288
remaining reads : 7

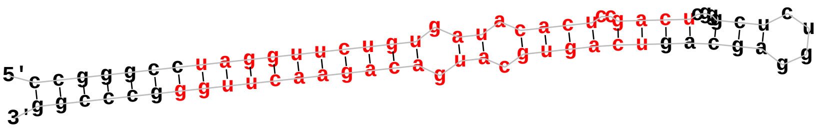

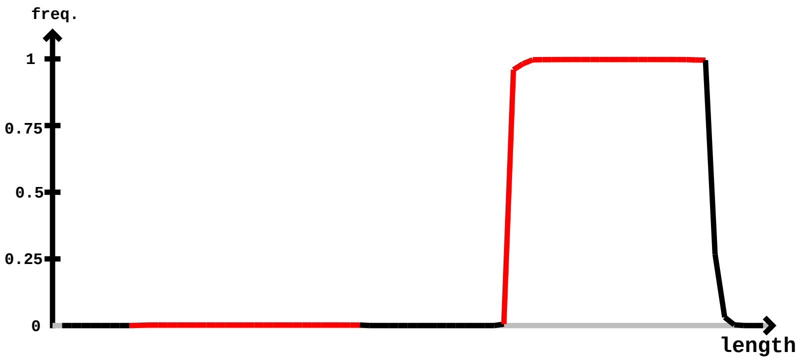


```
                                                         mmu-miR-152-3p
           mmu-miR-152-5p
5'- ccgggccuagguucugugauacacuccgacucgggcucuggagcagucagugcaugacagaacuugggcccgg   -3'    exp
    (((((((((((((((((.((.((((..((((...(((....))))))))))).)).)))))))))))))))))   reads   mm   sample
    .......uagguucugugauacac.................................................   1       0    seq
    .......uagguucugugauacacu................................................   2       0    seq
    .......uagguucugugauacacuc...............................................   1       0    seq
    .......uagguucugugauacacucc..............................................   3       0    seq
    .......uagguucugugauacacuccg.............................................   6       0    seq
    .......uagguucugugauCcacuccg.............................................   1       1    seq
    .......uagguucugugauacacuccga............................................   13      0    seq
    .......uagguucugugauacacuccgU............................................   3       1    seq
    .......uagguucugugauacacucUgC............................................   1       2    seq
    .......uagguucugugauacacuccgGU...........................................   1       2    seq
    .......uagguucugugauacacuccgac...........................................   3       0    seq
    .......uaUguucugugauacacuccgac...........................................   1       1    seq
    .......uagguucugCgauacacuccgacu..........................................   1       1    seq
    .......uagguucugugauacacuccgacu..........................................   171     0    seq
    .......uagCuucugugauacacuccgacu..........................................   1       1    seq
    .......uagguucugugauacacuccgacA..........................................   1       1    seq
    .......uagguucugugauacacuccgacG..........................................   2       1    seq
    .......uaCguucugugauacacuccgacu..........................................   1       1    seq
    .......uagguucugugauacacuccgaGu..........................................   1       1    seq
    .......uagguucugugauacacuccgaAG..........................................   2       2    seq
    .......uagUuucugugauacacuccgacu..........................................   1       1    seq
    .......uagguucugugauacacuccgacuA.........................................   1       1    seq
    ........agguucugugauacacu................................................   1       0    seq
    ........agguucugugauacacuc...............................................   1       0    seq
    ........agguucugugauacacucc..............................................   1       0    seq
    ........agguucugugauacacuccg.............................................   4       0    seq
    ........agguucugugauacacuccga............................................   3       0    seq
    ........agguucugugauacacuccgac...........................................   14      0    seq
    ........agguucugugauGcacuccgacu..........................................   1       1    seq
    ........agguucugugauacacuccgaAu..........................................   2       1    seq
    ........agguucugugauacacuccgaAC..........................................   1       2    seq
    ........agguucugugaGacacuccgacu..........................................   1       1    seq
    ........agUuucugugauacacuccgacu..........................................   1       1    seq
    ........agguucugugauacacuccgacu..........................................   238     0    seq
```

```
                                              mmu-miR-152-3p
       mmu-miR-152-5p
ccgggccuagguucugugauacacuccgacucgggcucuggagcagucagugcaugacagaacuugggcccgg

........agguucugugaCacacuccgacu..........................................   1          1          seq
........aCguucugugauacacuccgacu..........................................   1          1          seq
........agguucugugauacacuccgacuU.........................................   1          1          seq
........agguucugugauacacuccgacuc.........................................   16         0          seq
........agguucugugauacacuccgacAA.........................................   1          2          seq
........agguucugugauacacucAgacucU........................................   1          2          seq
........agguucugugauacacuccgacucU........................................   12         1          seq
........agguucugugauacacuccgacucA........................................   1          1          seq
.........gguucugugauacacuccgacu..........................................   5          0          seq
..........guucugugauacacuccga............................................   1          0          seq
...........uucugugauacacuccga............................................   1          0          seq
...........uucugugauacacuccgacA..........................................   1          1          seq
...........uucugugauacacuccgacu..........................................   6          0          seq
...........uucugugauacacuccgacucggU......................................   1          1          seq
..........................................UcUgucagugcaugacagaacuugg......   1          2          seq
...........................................cUgucagugcaugacagaacuugg......   1          1          seq
...........................................cGUucagugcaugacagaacuugg......   2          2          seq
...........................................cGAucagugcaugacagaacuugg......   1          2          seq
...........................................UCgucagugcaugacagaacuugg......   1          2          seq
...........................................cagucagugcaugacagaacuuggU.....   1          1          seq
............................................GAucagugcaugacagaacuugg......   1          2          seq
............................................Cgucagugcaugacagaacuugg......   1          1          seq
............................................UCucagugcaugacagaacuugg......   3          2          seq
............................................CUucagugcaugacagaacuugg......   1          2          seq
............................................Cgucagugcaugacagaacuuggg.....   1          1          seq
.............................................gucagugcaugacagaacuug.......   5          0          seq
.............................................gucagugcaugacagaacuugg......   987        0          seq
.............................................CucagugcaugacagaacuugU......   1          2          seq
.............................................gucagugcaugacagaacuugU......   4          1          seq
.............................................gucagugcaugCcagaacuugg......   1          1          seq
.............................................Cucagugcaugacagaacuugg......   21         1          seq
.............................................gucaguUcaugacagaacuugg......   1          1          seq
.............................................Aucagugcaugacagaacuugg......   1          1          seq
.............................................gCcagugcaugacagaacuugg......   1          1          seq
.............................................gucagugcauAacagaacuugg......   2          1          seq
.............................................CucagugcaugacagaacuuggU.....   1          2          seq
.............................................gucagugcaugacagaacuuggA.....   99         1          seq
.............................................gucagugcaugacagaacuuggU.....   73         1          seq
.............................................gucagugcaugacagaacuugUA.....   1          2          seq
.............................................gucagugcaugacagaacuuCgA.....   1          2          seq
.............................................gCcagugcaugacagaacuuggA.....   1          2          seq
.............................................gucagugcaugacagaacuuggC.....   2          1          seq
.............................................gucagugcaugacagaacuuggg.....   1          0          seq
.............................................CucagugcaugacagaacuuggA.....   8          2          seq
.............................................gucagugcauAacagaacuuggA.....   1          2          seq
.............................................gucagugcaugacagaacuuggUG....   2          2          seq
.............................................gucagugcaugacagaacuuggAU....   2          2          seq
.............................................gucagugcaugacagaacuuggUA....   1          2          seq
.............................................AucagugcaugacagaacuugggA....   1          2          seq
.............................................gucagugcaugacagaacuugAgU....   1          2          seq
..............................................ucagugcaugacagaac..........   24         0          seq
..............................................ucagugcaugacagaacu.........   48         0          seq
..............................................ucagCgcaugacagaacuu........   1          1          seq
..............................................ucagugcaugacagaCcuu........   1          1          seq
..............................................ucagugcaugacagGacuu........   1          1          seq
..............................................ucagugcaugGcagaacuu........   1          1          seq
..............................................ucagugcaugacagaacuu........   244        0          seq
..............................................ucagGgcaugacagaacuu........   1          1          seq
..............................................ucaguAcaugacagaacuu........   1          1          seq
..............................................ucagAgcaugacagaacuu........   1          1          seq
..............................................ucagugcaugacagaacuuU.......   6          1          seq
..............................................ucagugcaugacagaacuuA.......   15         1          seq
..............................................ucagugcaugacaCaacuug.......   1          1          seq
..............................................ucagugcaugacagaacuuC.......   1          1          seq
..............................................ucagugcaAgacagaacuug.......   1          1          seq
..............................................ucagugcaugacagaacuug.......   165        0          seq
..............................................ucagugcauCacagaacuug.......   1          1          seq
..............................................ucagugcaugacagaacuCC.......   1          2          seq
..............................................ucagugcaugaUagaacuug.......   2          1          seq
```

mmu-miR-152-3p

mmu-miR-152-5p

ccgggccuagguucugugauacacuccgacucgggcucuggagcagucagugcaugacagaacuugggcccgg

....ucagugcaugacagaacuAU.......	1	2	seq
....ucagugcaugacagaacuGg.......	4	1	seq
....ucagugcaugacagaCcuug.......	1	1	seq
....ucagugcauAacagaacuugC......	1	2	seq
....ucagugcaugacCgaCcuugg......	1	2	seq
....ucagugcaugacGgaacuugg......	48	1	seq
....Ccagugcaugacagaacuugg......	38	1	seq
....ucagCgcaugacagaacuugg......	140	1	seq
....ucagCgcaugacagUacuugg......	1	2	seq
....ucGgugcaugacagaacuugA......	1	2	seq
....ucagugcaugacagaacuugC......	56	1	seq
....ucagugcaugacagaacuuUU......	12	2	seq
....ucagugcaugacagaacuuAA......	12	2	seq
....ucaguAcaugacagaacuugg......	48	1	seq
....ucagugcaugaGagaacuugg......	8	1	seq
....ucagugcaugacagaaUuugU......	1	2	seq
....uUagugcaugacagaacuugU......	1	2	seq
....ucagugcaugacagaacuugg......	154553	0	seq
....ucagugcaugacagaacuuUg......	16	1	seq
....ucagugcaugacagaacuCgA......	3	2	seq
....ucUgugcaugacagaacuugg......	5	1	seq
....uGagugcaugacagaacuugg......	6	1	seq
....uGagugcaugacagaacuugU......	1	2	seq
....ucagugcCugacagCacuugg......	1	2	seq
....ucaUugcaugacagaacuugA......	1	2	seq
....ucagugcaugGcagaacuugg......	29	1	seq
....ucaguUcaugacagaacuugg......	28	1	seq
....ucagugcaugacagaaUuugg......	32	1	seq
....ucagugcaugGcagaUcuugg......	1	2	seq
....ucagAgcaugacagaacuugA......	1	2	seq
....ucagugcaugacagaacuugU......	1553	1	seq
....ucagugcaugUcagaacuugU......	1	2	seq
....ucagugGaugacagaacuugg......	9	1	seq
....ucagugcaugacagaacuGgg......	2	1	seq
....ucagugcaugacagUacuugg......	9	1	seq
....ucagugcaAgacagaacuugg......	12	1	seq
....ucagugcaugacagaacuCgg......	59	1	seq
....ucagugcaugacagaacuuCg......	43	1	seq
....ucagugcaugaAagaacuugg......	9	1	seq
....ucCgGgcaugacagaacuugg......	1	2	seq
....CcagugcaCgacagaacuugg......	1	2	seq
....ucagugcaugacagaaGuugU......	1	2	seq
....uUagugcaugacUgaacuugg......	1	2	seq
....ucagugcaugacagaacuuAg......	33	1	seq
....ucCgugcaugacagaacuugg......	50	1	seq
....uAagugcaugacagaacuugg......	14	1	seq
....ucaguAcaugacagaGcuugg......	1	2	seq
....ucagugcaugacagaacuuAC......	1	2	seq
....ucagugcCugacagaacuugg......	42	1	seq
....ucagugcUugacagaacuugg......	9	1	seq
....ucaUugcaugacagaacuugg......	67	1	seq
....ucagugUaugCcagaacuugg......	1	2	seq
....ucagugcaugacaUaacuugg......	10	1	seq
....ucagugcaugacagaacuCgU......	4	2	seq
....ucagugcaugacagaCcuugg......	53	1	seq
....Acagugcaugacagaacuugg......	18	1	seq
....ucagugcaugacagaacAugg......	12	1	seq
....ucagCUcaugacagaacuugg......	5	2	seq
....ucagugcaugCcagaacuugg......	41	1	seq
....ucagugcauCacagaacuugC......	1	2	seq
....ucagugcaugacagaacGugg......	8	1	seq
....ucagugcauCacagaacuugg......	48	1	seq
....ucagugcaugacagaacuugA......	642	1	seq
....ucagugcauAacagaacuugg......	40	1	seq
....ucaAugcaugacagaacuugg......	45	1	seq
....ucagugAaugacagaacuugg......	16	1	seq
....ucagugcaugacagaaACugg......	1	2	seq
....ucagugcaugacaAaacuugg......	15	1	seq
....ucagAgcaGgacagaacuugg......	1	2	seq

```
                                              mmu-miR-152-3p
       mmu-miR-152-5p
ccgggccuagguucugugauacacuccgacucgggcucuggagcagucagugcaugacagaacuugggcccgg

..............................................ucagugcaugacCgaacuugA......    1    2    seq
..............................................ucagugcaugacaCaacuugg......    30   1    seq
..............................................ucagugcaugacagCacuugg......    59   1    seq
..............................................ucagGgcaugacagCacuugg......    1    2    seq
..............................................ucagugcaugacagaCcuugU......    1    2    seq
..............................................ucagugcaugacagaGcuugg......    22   1    seq
..............................................ucagugcaugacagaacuuCU......    4    2    seq
..............................................ucagugcaGgacagaacuugg......    8    1    seq
..............................................Gcagugcaugacagaacuugg......    4    1    seq
..............................................ucagugcaugCcUgaacuugg......    1    2    seq
..............................................ucagAgcaugacagaacuugg......    34   1    seq
..............................................ucagugcaugCcagaCcuugg......    1    2    seq
..............................................ucagGgcaugacagaacuGgg......    1    2    seq
..............................................ucagugcaugacagaacuGgU......    1    2    seq
..............................................ucagugcaugacagaUcuugg......    5    1    seq
..............................................ucagugcaugacUgaacuugg......    19   1    seq
..............................................ucagugcauCacagaacuugU......    4    2    seq
..............................................ucagugcaugacaCaacuugU......    1    2    seq
..............................................ucagugcauUacagaacuugg......    56   1    seq
..............................................ucagugcaugacagaCAuugg......    1    2    seq
..............................................ucagCgcCugacagaacuugg......    1    2    seq
..............................................ucagGgcaugacagaacuuCg......    1    2    seq
..............................................ucagugUaugacagaacuugg......    57   1    seq
..............................................ucagugcaugacCgaacuugg......    36   1    seq
..............................................ucagugcaugacagaaGuugg......    1    1    seq
..............................................ucagugcGugacagaacuCgg......    1    2    seq
..............................................ucUUugcaugacagaacuugg......    1    2    seq
..............................................ucaCugcaugacagaacuugg......    58   1    seq
..............................................ucagugcaugaUagaacuugg......    50   1    seq
..............................................ucagGgcauUacagaacuugg......    1    2    seq
..............................................ucagCgcaugacagaacuCgg......    1    2    seq
..............................................ucagugcaCgacagaacuugg......    46   1    seq
..............................................ucagCgcaugacagaacuugU......    1    2    seq
..............................................ucagugcaugacagaaAuugg......    8    1    seq
..............................................ucaUugcaugacagaacuCgg......    1    2    seq
..............................................ucagGgcaugacagaacuugg......    74   1    seq
..............................................ucagugcaugacagGacuugg......    21   1    seq
..............................................ucagugcaCgacagCacuugg......    1    2    seq
..............................................uUagugcaugacagaacuugg......    49   1    seq
..............................................ucagugcGugacagaacuugg......    43   1    seq
..............................................ucaCugcaugacGgaacuugg......    1    2    seq
..............................................ucaAugcaugacagaacuuAg......    1    2    seq
..............................................CcagugcaugacagCacuugg......    1    2    seq
..............................................ucagugcaugacagCacuugU......    2    2    seq
..............................................ucaUugcauUacagaacuugg......    2    2    seq
..............................................ucagugcaugacagaacuAgg......    4    1    seq
..............................................ucaguCcaugacagaacuugg......    47   1    seq
..............................................AcagugcaugacagaaAuugg......    1    2    seq
..............................................ucagugcCugacagaaUuugg......    1    2    seq
..............................................ucagugcaugUcagaacuugg......    34   1    seq
..............................................ucagugcaugacagaacCugU......    1    2    seq
..............................................ucagugcaugacagaacCugg......    67   1    seq
..............................................ucGgugcaugacagaacuugg......    47   1    seq
..............................................ucaCugcaugacagaacuugU......    1    2    seq
..............................................ucagugcCugacagaacuuggg.....    1    1    seq
..............................................ucaAugcaugacagaacuuggA.....    2    2    seq
..............................................ucagugGaugacagaacuuggU.....    2    2    seq
..............................................ucUgugcaugacagaacuuggU.....    3    2    seq
..............................................ucagugcaugacaAaacuuggA.....    1    2    seq
..............................................ucagugcaugaUagaacuuggA.....    9    2    seq
..............................................ucagugcUugacagaacuuggA.....    2    2    seq
..............................................ucagugcaugGcagaacuuggg.....    4    1    seq
..............................................ucagugcauUacagaacuuggA.....    2    2    seq
..............................................ucagugcaugacagaUcuuggU.....    1    2    seq
..............................................ucagugcaugacUgaacuuggU.....    2    2    seq
..............................................ucagugcaugacagaacuuUgA.....    7    2    seq
..............................................ucagugcaugacagaaUuuggA.....    4    2    seq
..............................................ucagugcaCgacagaacuuggA.....    8    2    seq
..............................................ucaUugcaugacagaacuuggA.....    6    2    seq
```

```
                                              mmu-miR-152-3p
       mmu-miR-152-5p
ccgggccuagguucugugauacacuccgacucgggcucuggagcagucagugcaugacagaacuugggcccgg

..............................................ucagugcaCgacagaacuuggU.....    8      2      seq
..............................................ucaguAcaugacagaacuuggg.....    1      1      seq
..............................................ucagugcaugacagaaAuuggU.....    1      2      seq
..............................................ucGgugcaugacagaacuuggA.....    7      2      seq
..............................................ucagGgcaugacagaacuuggA.....    6      2      seq
..............................................ucCgugcaugacagaacuuggA.....    8      2      seq
..............................................ucagugcaugCcagaacuuggA.....    4      2      seq
..............................................ucagugcaugacagaGcuuggU.....    3      2      seq
..............................................ucagugcaugacagaacuugAA.....    23     2      seq
..............................................ucaguUcaugacagaacuuggU.....    2      2      seq
..............................................ucagugcaCgacagaacuuggg.....    5      1      seq
..............................................ucagugcaugacagaacAuggU.....    2      2      seq
..............................................ucagugcaugaUagaacuuggU.....    4      2      seq
..............................................ucagugcaugacagaUcuuggC.....    1      2      seq
..............................................ucagGgcaugacagaacuuggg.....    1      1      seq
..............................................ucagugcaugacaAaacuuggg.....    1      1      seq
..............................................ucagugcaugacagaacuugCU.....    2      2      seq
..............................................ucaguAcaugacagaacuuggA.....    6      2      seq
..............................................uAagugcaugacagaacuuggA.....    3      2      seq
..............................................ucagugcaugacaCaacuuggU.....    2      2      seq
..............................................ucagugcaugacagaaUuuggU.....    2      2      seq
..............................................ucaguAcaugacagaacuuggU.....    2      2      seq
..............................................ucagugcaugacagCacuuggg.....    2      1      seq
..............................................ucaguCcaugacagaacuuggA.....    5      2      seq
..............................................ucagugcaugacagaacuuCgU.....    8      2      seq
..............................................CcagugcaugacagaacuuggU.....    3      2      seq
..............................................ucagugcaugacagaacuugCg.....    1      1      seq
..............................................ucaCugcaugacagaacuuggA.....    8      2      seq
..............................................ucagAgcaugacagaacuuggU.....    4      2      seq
..............................................uUagugcaugacagaacuuggA.....    4      2      seq
..............................................ucagugcaugacagaacuGggU.....    1      2      seq
..............................................ucCgugcaugacagaacuuggU.....    4      2      seq
..............................................ucagugcaugacaUaacuuggU.....    2      2      seq
..............................................uGagugcaugacagaacuuggU.....    3      2      seq
..............................................ucagugcauCacagaacuuggA.....    8      2      seq
..............................................ucagugcaugacagaGcuuggA.....    1      2      seq
..............................................ucagugcCugacagaacuuggU.....    2      2      seq
..............................................ucaAugcaugacagaacuuggU.....    6      2      seq
..............................................ucaguUcaugacagaacuuggA.....    2      2      seq
..............................................ucagugcaugCcagaacuuggg.....    3      1      seq
..............................................ucagCgcaugacagaacuuggA.....    15     2      seq
..............................................ucagugcaugacagaacuuCgg.....    1      1      seq
..............................................ucagugcaugCcagaacuuggU.....    6      2      seq
..............................................ucUgugcaugacagaacuuggg.....    2      1      seq
..............................................ucagugcaugacCgaacuuggg.....    1      1      seq
..............................................ucagugcCugacagaacuuggA.....    5      2      seq
..............................................ucagugcaugaGagaacuuggU.....    1      2      seq
..............................................ucagugcGugacagaacuuggU.....    6      2      seq
..............................................ucagugcaugaAagaacuuggA.....    1      2      seq
..............................................ucagugcaugaAagaacuuggU.....    1      2      seq
..............................................ucagugcaugacagaacuAggU.....    3      2      seq
..............................................uAagugcaugacagaacuuggU.....    2      2      seq
..............................................GcagugcaugacagaacuuggA.....    1      2      seq
..............................................ucagugcaugacagaacuuggC.....    168    1      seq
..............................................ucagugcauUacagaacuuggg.....    4      1      seq
..............................................ucagugcaugaGagaacuuggA.....    3      2      seq
..............................................ucagugcaugaGagaacuuggg.....    1      1      seq
..............................................GcagugcaugacagaacuuggU.....    1      2      seq
..............................................ucagugcGugacagaacuuggA.....    3      2      seq
..............................................ucagugcauUacagaacuuggU.....    4      2      seq
..............................................ucagugcaugacagaacuugUA.....    33     2      seq
..............................................ucagugcaugacagaacuGggg.....    1      1      seq
..............................................ucaguUcaugacagaacuuggg.....    2      1      seq
..............................................ucagugcaugacagaacuuCgA.....    9      2      seq
..............................................ucaCugcaugacagaacuuggU.....    1      2      seq
..............................................ucagugcaugacagaacCuggg.....    3      1      seq
..............................................ucagugcaugacagaacCuggA.....    6      2      seq
..............................................ucGgugcaugacagaacuuggU.....    3      2      seq
..............................................ucagugcaugacagaacuugCA.....    3      2      seq
```

```
                                                    mmu-miR-152-3p
       mmu-miR-152-5p
ccgggccuagguucugugauacacuccgacucgggcucuggagcagucagugcaugacagaacuugggcccgg

..............................................ucagugcaugacGgaacuuggU.....   7       2         seq
..............................................ucagugcaugacagaacCuggU.....   12      2         seq
..............................................ucagugUaugacagaacuuggU.....   8       2         seq
..............................................ucagugcaugacagaaUuuggg.....   1       1         seq
..............................................ucagugcaugacagaacuugUg.....   1       1         seq
..............................................ucagugcaugacagaacuCggg.....   7       1         seq
..............................................AcagugcaugacagaacuuggA.....   3       2         seq
..............................................ucagugcaugacagCacuuggA.....   5       2         seq
..............................................ucagugcauCacagaacuuggg.....   1       1         seq
..............................................ucagugcaugGcagaacuuggU.....   7       2         seq
..............................................Gcagugcaugacagaacuuggg.....   2       1         seq
..............................................ucagugUaugacagaacuuggA.....   6       2         seq
..............................................ucagugcaugacCgaacuuggA.....   4       2         seq
..............................................ucaguCcaugacagaacuuggU.....   3       2         seq
..............................................ucagugcaugacagaacuuggA.....   21391   1         seq
..............................................ucagugcaGgacagaacuuggA.....   1       2         seq
..............................................ucagugcaugacagaacuuAgg.....   2       1         seq
..............................................ucagugcaugacagaCcuuggU.....   4       2         seq
..............................................ucagGgcaugacagaacuuggU.....   5       2         seq
..............................................ucagugcaAgacagaacuuggA.....   1       2         seq
..............................................ucagugcaugacagaacuuggU.....   22007   1         seq
..............................................ucagugcaugaUagaacuuggg.....   5       1         seq
..............................................AcagugcaugacagaacuuggU.....   3       2         seq
..............................................ucagugcaugacagaacuuggg.....   6975    0         seq
..............................................ucaUugcaugacagaacuuggU.....   6       2         seq
..............................................ucagugcaugacGgaacuuggA.....   8       2         seq
..............................................ucagugcaugacagaacuCggU.....   6       2         seq
..............................................ucagugcaugacaUaacuuggg.....   1       1         seq
..............................................ucagugcaugacagaacuCggA.....   9       2         seq
..............................................ucagAgcaugacagaacuuggA.....   3       2         seq
..............................................ucagugcaugGcagaacuuggA.....   2       2         seq
..............................................ucagugcUugacagaacuuggU.....   2       2         seq
..............................................uUagugcaugacagaacuuggg.....   1       1         seq
..............................................ucagugcaugUcagaacuuggU.....   7       2         seq
..............................................ucagugcauAacagaacuuggA.....   5       2         seq
..............................................ucaAugcaugacagaacuuggg.....   2       1         seq
..............................................ucaUugcaugacagaacuuggg.....   1       1         seq
..............................................Ccagugcaugacagaacuuggg.....   2       1         seq
..............................................ucagugcaugacagaacuuAgA.....   7       2         seq
..............................................ucagugcaugacCgaacuuggU.....   6       2         seq
..............................................ucaguCcaugacagaacuuggg.....   2       1         seq
..............................................ucagugcaugacagaacuugAU.....   8       2         seq
..............................................ucagugcaugacagGacuuggA.....   2       2         seq
..............................................uUagugcaugacagaacuuggU.....   9       2         seq
..............................................ucagugcaugacagaCcuuggA.....   4       2         seq
..............................................ucagugcaugacagGacuuggU.....   5       2         seq
..............................................ucagugcaugacagCacuuggU.....   15      2         seq
..............................................ucagugcaugacagaacuuUgg.....   2       1         seq
..............................................ucagugcaugacGgaacuuggg.....   4       1         seq
..............................................ucagugcaugacaCaacuuggA.....   4       2         seq
..............................................ucagugcaugacagaacuugUU.....   56      2         seq
..............................................ucagugAaugacagaacuuggA.....   1       2         seq
..............................................ucagCgcaugacagaacuuggg.....   6       1         seq
..............................................ucagugcaugacagaUcuuggA.....   3       2         seq
..............................................ucagCgcaugacagaacuuggU.....   15      2         seq
..............................................ucagugcaugacagaaAuuggA.....   1       2         seq
..............................................ucagugcaugUcagaacuuggA.....   1       2         seq
..............................................ucagugcaugacagaacuuAgU.....   6       2         seq
..............................................ucagugGaugacagaacuuggA.....   1       2         seq
..............................................CcagugcaugacagaacuuggA.....   5       2         seq
..............................................ucagugcaugacagaacuugAg.....   5       1         seq
..............................................ucagugcauAacagaacuuggU.....   9       2         seq
..............................................ucagugcaugacagaacuGgggA....   1       2         seq
..............................................ucagugcaugacagaacuugggU....   923     1         seq
..............................................ucCgugcaugacagaacuugggU....   1       2         seq
..............................................ucagugcaugacagaacuuggAA....   808     2         seq
..............................................ucagugcaugacagaacuuUggU....   1       2         seq
..............................................ucagugcaugacagaUcuugggU....   1       2         seq
..............................................ucagugcaugacagaacuugCgU....   1       2         seq
```

```
                                              mmu-miR-152-3p
       mmu-miR-152-5p
ccgggccuagguucugugauacacuccgacucgggcucuggagcagucagugcaugacagaacuugggcccgg

..............................................ucagGgcaugacagaacuugggU....    1       2       seq
..............................................ucagugcaugacagaacuugAgA....    1       2       seq
..............................................ucagugcaugacagaacuuggAc....    68      1       seq
..............................................ucagugcaugacagaCcuugggA....    1       2       seq
..............................................ucagugcaugacagaacuugggG....    8       1       seq
..............................................ucagugcaugacagaacuuggUA....    1176    2       seq
..............................................ucagAgcaugacagaacuugggU....    1       2       seq
..............................................ucagugcaugUcagaacuugggA....    1       2       seq
..............................................ucagugcCugacagaacuugggc....    1       1       seq
..............................................ucagCgcaugacagaacuugggU....    1       2       seq
..............................................ucaguAcaugacagaacuugggU....    1       2       seq
..............................................ucagugcaugaUagaacuugggU....    2       2       seq
..............................................ucUgugcaugacagaacuugggA....    1       2       seq
..............................................ucagugcaugacagaacuuggAG....    160     2       seq
..............................................ucagugUaugacagaacuugggA....    1       2       seq
..............................................ucagugcaugacagaacuugggA....    1324    1       seq
..............................................ucagugcaugacagaacuuggUc....    7       1       seq
..............................................ucagugcaugacagaacuugUgU....    5       2       seq
..............................................ucagugcaCgacagaacuugggU....    1       2       seq
..............................................uUagugcaugacagaacuugggU....    1       2       seq
..............................................ucagugcaugacagaaUuuggUc....    1       2       seq
..............................................ucagugcaugacagaacuuggCA....    9       2       seq
..............................................ucagugcaugacagaacuuggUU....    1235    2       seq
..............................................ucagugcaugacagaacuugUgA....    2       2       seq
..............................................ucagugcaugacagCacuugggA....    2       2       seq
..............................................ucagugcaugacagaacuuCggA....    1       2       seq
..............................................ucaguAcaugacagaacuuggAc....    1       2       seq
..............................................ucagGgcaugacagaacuugggA....    2       2       seq
..............................................ucagugcaugGcagaacuugggA....    1       2       seq
..............................................ucagugcaugacagaacCugggA....    2       2       seq
..............................................ucagugcaugacagaacuugggc....    19      0       seq
..............................................ucagugcaugacagaacuugAAc....    1       2       seq
..............................................ucagugcaugacagaacuuggUG....    39      2       seq
..............................................ucagugcaugacagaacuuggAU....    382     2       seq
..............................................ucagugcaugacagaacuuggCU....    10      2       seq
..............................................ucagugcGugacagaacuugggU....    1       2       seq
..............................................ucagugcaugacagaacuuggUcc...    2       1       seq
..............................................ucagugcaugacagaacuuggAcA...    7       2       seq
..............................................ucagugcaugacagaacuugggAU...    152     2       seq
..............................................ucagugcaugacagaacuugggUc...    1       1       seq
..............................................ucagugcaugacagaacuuggAGc...    2       2       seq
..............................................ucagugcaugacagaacuugggUG...    2       2       seq
..............................................ucagugcaugacagaacuugggcA...    2       1       seq
..............................................ucagugcaugacagaacuugggUU...    107     2       seq
..............................................ucagugcaugacagaacuugggUA...    62      2       seq
..............................................ucagugcaugacagaacuuggUUc...    2       2       seq
..............................................ucagugcaugacagaacuuggUcU...    3       2       seq
..............................................ucagugcaugacagaacuuggAcU...    3       2       seq
..............................................ucagugcaugacagaacuugggGA...    2       2       seq
..............................................ucagugcaugacagaacuugggAc...    16      1       seq
..............................................ucagugcaugacagaacuugggAA...    98      2       seq
..............................................ucagugcaugacagaacuugggAG...    40      2       seq
..............................................ucagugcaugacagaacuuggUcG...    1       2       seq
..............................................ucagugcaugacagaacuuggAAc...    58      2       seq
..............................................ucagugcaugacagaacuuggUAc...    27      2       seq
..............................................ucagugcaugacagaacuuggUAcc..    2       2       seq
..............................................ucagugcaugacagaacuuggAcGc..    2       2       seq
..............................................ucagugcaugacagaacuugggUAc..    6       2       seq
..............................................ucagugcaugacagaacuugggAAc..    14      2       seq
..............................................ucagugcaugacagaacuugggcUU..    1       2       seq
..............................................ucagugcaugacagaacuugggAcU..    2       2       seq
...............................................cagugcaugacagaacu.........    4       0       seq
...............................................cagugcaugacagaacuu........    1       0       seq
...............................................cagugcaugacagaacuug.......    3       0       seq
...............................................cCgugcaugacagaacuugg......    1       1       seq
...............................................cagugcaugacagaacuugU......    30      1       seq
...............................................cagugcaugaAagaacuugA......    1       2       seq
...............................................cagugcaugacCgaacuugg......    1       1       seq
...............................................cagCgcaugacagaacuugg......    1       1       seq
```

```
                                              mmu-miR-152-3p
       mmu-miR-152-5p
ccgggccuagguucugugauacacuccgacucgggcucuggagcagucagugcaugacagaacuugggcccgg

...............................................cagugcaugacagaacuuCU......    1          2          seq
...............................................cagugcaGgacagaacuugg......    1          1          seq
...............................................cagugcaugacagaacuugC......    1          1          seq
...............................................cagugcGugacagaacuugg......    2          1          seq
...............................................cagugcaugacaCaacuugg......    2          1          seq
...............................................cagugcaugacagaCcuugg......    2          1          seq
...............................................cagugcauAacagaacuugg......    1          1          seq
...............................................cagugcaugaUagaacuugg......    2          1          seq
...............................................cagGgcaugacagaacuugg......    10         1          seq
...............................................cagugAaugacagaacuugg......    1          1          seq
...............................................caguAcaugacagaacuugg......    3          1          seq
...............................................Aagugcaugacagaacuugg......    8          1          seq
...............................................cagugcaugacagaacuGgg......    2          1          seq
...............................................Uagugcaugacagaacuugg......    2          1          seq
...............................................cagugcaugacagaacuugA......    12         1          seq
...............................................cagugcaugGcagaacuugg......    1          1          seq
...............................................cagugcCugacagaacuugg......    1          1          seq
...............................................caguCcaugacagaacuugg......    1          1          seq
...............................................caCugcaugacagaacuugg......    3          1          seq
...............................................cagugcaugacagaacuugg......    3424       0          seq
...............................................cagugcaugacagaacuCgg......    1          1          seq
...............................................cagugcaugaAagaacuugg......    3          1          seq
...............................................Gagugcaugacagaacuugg......    2          1          seq
...............................................cagugcaugacagCacuugg......    1          1          seq
...............................................cagugcaCgacagaacuugg......    4          1          seq
...............................................caAugcaugacagaacuugg......    1          1          seq
...............................................cagugcaugacagaaUuugg......    2          1          seq
...............................................cagugcaugCcagaacuugg......    2          1          seq
...............................................cagugcaugacagaacuAgg......    1          1          seq
...............................................caAugcaugacagaacuuggU.....    1          2          seq
...............................................cagugcaugacagaacuuCgA.....    1          2          seq
...............................................AagugcaugacagaacuuggA.....    1          2          seq
...............................................cagugcaugaUagaacuuggU.....    1          2          seq
...............................................cagugcaugacagaacuuggg.....    154        0          seq
...............................................cagugcaugaAagaacuuggA.....    1          2          seq
...............................................cagCgcaugacagaacuuggg.....    1          1          seq
...............................................cagugcGugacagaacuuggU.....    1          2          seq
...............................................cagugcauAacagaacuuggA.....    1          2          seq
...............................................cagCgcaugacagaacuuggU.....    1          2          seq
...............................................cagugcaugaGagaacuuggA.....    1          2          seq
...............................................cagugcaugacagaacuuCgU.....    1          2          seq
...............................................GagugcaugacagaacuuggA.....    1          2          seq
...............................................cagugcaugacagaacuugUA.....    2          2          seq
...............................................AagugcaugacagaacuuggU.....    2          2          seq
...............................................cagugcaugacaCaacuuggg.....    1          1          seq
...............................................cagugcaugacagCacuuggA.....    2          2          seq
...............................................cagugcaugacGgaacuuggU.....    2          2          seq
...............................................cagugcGugacagaacuuggg.....    1          1          seq
...............................................cagugcaugacagaacuuggC.....    8          1          seq
...............................................cagugcaugacagaacuuggU.....    593        1          seq
...............................................cagugGaugacagaacuuggU.....    1          2          seq
...............................................cagGgcaugacagaacuuggU.....    3          2          seq
...............................................cagugcaugacagaacuuggA.....    606        1          seq
...............................................cagugcaugCcagaacuuggA.....    4          2          seq
...............................................cagugcaugacaUaacuuggA.....    1          2          seq
...............................................cagGgcaugacagaacuuggA.....    1          2          seq
...............................................cagugcaugCcagaacuuggU.....    3          2          seq
...............................................cagugcCugacagaacuuggA.....    1          2          seq
...............................................cagugcaugacagaacuugUU.....    2          2          seq
...............................................cagugcaugacCgaacuuggU.....    1          2          seq
...............................................cagugcaugacagaacuuggAc....    2          1          seq
...............................................cagugcaugacagaacuuggCU....    1          2          seq
...............................................cagugcaugacagaacuuggUU....    38         2          seq
...............................................cagugcaugCcagaacuugggA....    1          2          seq
...............................................cagugcaugacagaacuuggUA....    26         2          seq
...............................................cagugcaugacagaacuuggAU....    7          2          seq
...............................................cagugcaugacagaacuuggCA....    1          2          seq
...............................................cagugcaugacagaacuugggA....    28         1          seq
...............................................cagugcaugacagaacuuggAA....    18         2          seq
```

```
                                              mmu-miR-152-3p
       mmu-miR-152-5p
ccgggccuagguucugugauacacuccgacucgggcucuggagcagucagugcaugacagaacuugggcccgg

...............................................cagugcaugacagaacuuggAG....   1     2    seq
...............................................cagugcaugacagaacuugggU....   19    1    seq
...............................................cagugcaugacagaacuugggAA...   1     2    seq
...............................................cagugcaugacagaacuugggAU...   6     2    seq
...............................................cagugcaugacagaacuuggAAc...   1     2    seq
...............................................cagugcaugacagaacuugggUU...   7     2    seq
...............................................cagugcaugacagaacuugggUA...   2     2    seq
...............................................cagugcaugacagaacuugggUAc..   1     2    seq
................................................agugcaugacagaacuu........   3     0    seq
................................................agugcaugacagaacuug.......   3     0    seq
................................................agugcaugacCgaacuugg......   2     1    seq
................................................aAugcaugacagaacuugg......   1     1    seq
................................................agugcaugacagaacuugU......   30    1    seq
................................................agugAaugacagaacuugg......   1     1    seq
................................................agugcaugacagaacuCgg......   3     1    seq
................................................agugcaugacagaacuugC......   1     1    seq
................................................agugcaugacagaacCugg......   1     1    seq
................................................agugcaugacagaacuuUU......   2     2    seq
................................................agGgcaugacagaacuugg......   2     1    seq
................................................agugcauUacagaacuugg......   4     1    seq
................................................agugcaugacagaacuugg......   2270  0    seq
................................................agugcaugacagCacuugg......   1     1    seq
................................................agugcaugacagaacuuAU......   1     2    seq
................................................aguCcaugacagaacuugg......   1     1    seq
................................................agugcaugacagaacuugA......   5     1    seq
................................................agugcauAacagaacuugg......   2     1    seq
................................................UCugcaugacagaacuugg......   3     2    seq
................................................CAugcaugacagaacuugg......   1     2    seq
................................................agugcaugacagaGcuugg......   1     1    seq
................................................agugcaugacGgaacuugg......   1     1    seq
................................................agugcaugUcagaacuugg......   1     1    seq
................................................agCgcaugacagaacuugg......   3     1    seq
................................................Cgugcaugacagaacuugg......   1     1    seq
................................................agugcauCacagaacuugg......   1     1    seq
................................................agugUaugacagaacuugg......   1     1    seq
................................................agugcaugacaUaacuugg......   1     1    seq
................................................CgGgcaugacagaacuugg......   1     2    seq
................................................agugcaugacaAaacuugU......   1     2    seq
................................................agugcaugacagaacuuggg.....   97    0    seq
................................................agugcauAacagaacuuggA.....   2     2    seq
................................................aguCcaugacagaacuuggA.....   1     2    seq
................................................agugcaugacaCaacuuggA.....   1     2    seq
................................................agCgcaugacagaacuuggA.....   2     2    seq
................................................agugAaugacagaacuuggU.....   1     2    seq
................................................agugcaugGcagaacuuggg.....   1     1    seq
................................................agCgcaugacagaacuuggU.....   1     2    seq
................................................agugcaCgacagaacuuggU.....   1     2    seq
................................................agugcaugacagaacuuggA.....   384   1    seq
................................................agugcaugacagaacuugCA.....   1     2    seq
................................................agugcaugacagaacuuggU.....   400   1    seq
................................................agugcaugaUagaacuuggU.....   1     2    seq
................................................agugcaugacGgaacuuggA.....   1     2    seq
................................................agugcaugacagaacuuCgU.....   1     2    seq
................................................agugcaugacagaacuuggC.....   4     1    seq
................................................aUugcaugacagaacuuggU.....   2     2    seq
................................................agugcaugacagaacuugUA.....   1     2    seq
................................................agugcaugacagaacuuggCA....   1     2    seq
................................................agugcaugacagaacuuggAG....   1     2    seq
................................................agugcaugacagaacuuggUU....   20    2    seq
................................................agugcaugacagaacuuggAU....   8     2    seq
................................................agugcaugacagaacuuggAA....   10    2    seq
................................................agugcaugacagaacuuggUA....   15    2    seq
................................................agugcaugacagaacuugggU....   16    1    seq
................................................agugcaugacagaacuugggG....   1     1    seq
................................................agugcaugacagaacuugggA....   22    1    seq
................................................agugcaugacagaacuuggAc....   1     1    seq
................................................agugcaugacagaacuugggUU...   1     2    seq
................................................agugcaugacagaacuugggAc...   1     1    seq
................................................agugcaugacagaacuugggAA...   2     2    seq
```

```
                                              mmu-miR-152-3p
       mmu-miR-152-5p
ccgggccuagguucugugauacacuccgacucgggcucuggagcagucagugcaugacagaacuugggcccgg

................................................agugcaugacagaacuugggAU...   2   2   seq
................................................agugcaugacagaacuuggUUc...   1   2   seq
................................................agugcaugacagaacuuggAAc...   1   2   seq
................................................agugcaugacagaacuugggUUc..   2   2   seq
................................................agugcaugacagaacuugggAAc..   1   2   seq
.................................................gCgcaugacagaacuugg......   1   1   seq
.................................................gugcaugacagaacuugg......   78  0   seq
.................................................Augcaugacagaacuugg......   1   1   seq
.................................................gugcaugacagaacuuggU.....   22  1   seq
.................................................gugcaugacagaacuuggg.....   2   0   seq
.................................................gugcaugacagaacuuggA.....   11  1   seq
.................................................gugcaugacagaacuuggAG....   1   2   seq
.................................................gugcaugacagaacuuggUU....   1   2   seq
..................................................ugcaugacagaacuugU......   1   1   seq
..................................................ugcaugacagaacuugg......   42  0   seq
..................................................ugcaugacagaacuugC......   1   1   seq
..................................................ugcaugacagaacuuggU.....   2   1   seq
..................................................ugcaugacagaacuuggg.....   1   0   seq
..................................................ugcaugacagaacuuggA.....   3   1   seq
..................................................ugcaugacagaacuuggUU....   1   2   seq
..................................................ugcaugacagaacuugggU....   1   1   seq
...................................................gcaugacagaacuuggA.....   19  1   seq
...................................................gcaugacagaacuuggg.....   5   0   seq
...................................................gcaugacagaacuuggU.....   23  1   seq
...................................................gcaugacagaacuugggA....   1   1   seq
...................................................gcaugacagaacuuggAA....   4   2   seq
...................................................gcaugacagaacuuggAG....   1   2   seq
...................................................gcaugacagaacuugggU....   2   1   seq
...................................................gcaugacagaacuugggUA...   1   2   seq
....................................................caugacagaacuuggUU....   1   2   seq
```
