# Supplementary material for: mirPRo–a novel standalone program for differential expression and variation analysis of miRNAs
Source: Sci Rep. 2015 Oct 5;5:14617. doi: 10.1038/srep14617 (PMC4592965; doi:10.1038/srep14617)
Supplement: Supplementary Data 12-21 [file srep14617-s25.zip › Supplementary Data 13.pdf]

miRBase precursor : mmu-mir-152
Total read count : 347248
mmu-miR-152-5p read count : 751
mmu-miR-152-3p read count : 346483
remaining reads : 14

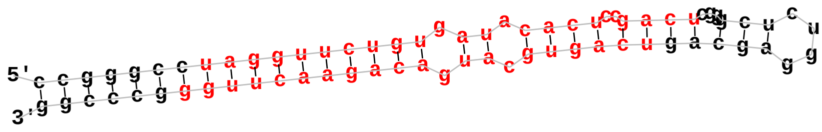

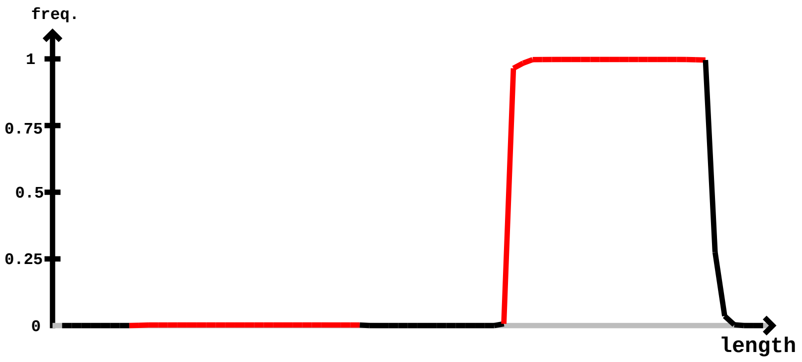

mmu-miR-152-3p
mmu-miR-152-5p

```
5'- ccgggccuagguucugugauacacuccgacucgggcucuggagcagucagugcaugacagaacuugggcccgg   -3'    exp
    ((((((((((((((((.((.((((..((((...(((....))))))))))).)).))))))))))))))))    reads      mm         sample
    .......uagguucugugauacacu................................................    1          0          seq
    .......uagguucugugauacacucc..............................................    2          0          seq
    .......uagguucugugauacacucGg.............................................    1          1          seq
    .......uagguucugugauacacuccg.............................................    6          0          seq
    .......uagguucugugauacacuccga............................................    15         0          seq
    .......uagguucugugauacacuccgU............................................    7          1          seq
    .......uagCuucugugauacacuccgU............................................    1          2          seq
    .......uagguucugugauacacuccgac...........................................    8          0          seq
    .......uagguucugugauacacuccgUU...........................................    4          2          seq
    .......uagguucugugauacacuccgaA...........................................    1          1          seq
    .......uagUuucugugauacacuccgacu..........................................    2          1          seq
    .......uagguucugugauacacuccgacA..........................................    3          1          seq
    .......uaggGucugugauacacuccgacu..........................................    1          1          seq
    .......uagguucugugauacacuccgaUu..........................................    1          1          seq
    .......uagguucugugauacacuccgacG..........................................    1          1          seq
    .......uagguucugugaGacacuccgacu..........................................    1          1          seq
    .......uagguucugugauacacuccgaAG..........................................    1          2          seq
    .......uagguucugugauacacuccgacu..........................................    244        0          seq
    .......uagguucugugauacacuccgacuU.........................................    2          1          seq
    .......uagguucugugauacacuccgacuAA........................................    1          2          seq
    .......uagguucugugauacacuccgacuUU........................................    1          2          seq
    .......uagguucugugauacacuccgacuAAg.......................................    1          2          seq
    .......uagguucugugauacacuccgacucgg.......................................    1          0          seq
    ........agguucugugauacacu................................................    1          0          seq
    ........agguucugugauacacucc..............................................    1          0          seq
    ........agguucugugauacacuccg.............................................    1          0          seq
    ........agguucuguUauacacuccga............................................    1          1          seq
    ........agguucugugauacacuccga............................................    2          0          seq
    ........agguucugugauacacuccgU............................................    1          1          seq
    ........agguucugugauacacuccgac...........................................    13         0          seq
    ........agguucuguUauacacuccgacu..........................................    1          1          seq
    ........agguucugugauacacuccgGcu..........................................    1          1          seq
    ........agUuucugugauacacuccgacu..........................................    4          1          seq
    ........agguucugugauacacAccgacu..........................................    1          1          seq
```

```
                                                 mmu-miR-152-3p
       mmu-miR-152-5p
ccgggccuagguucugugauacacuccgacucgggcucuggagcagucagugcaugacagaacuugggcccgg

........agguCcugugauacacuccgacu..........................................    1      1      seq
........agguucugugauacacuccgacA..........................................    5      1      seq
........agguucugugauacacuAcgacu..........................................    1      1      seq
........agguucugugauacacuccgacu..........................................    379    0      seq
........agguucuUugauacacuccgacA..........................................    1      2      seq
........agguucugugaCacacuccgacu..........................................    1      1      seq
........agguucugugauacacuccgacuc.........................................    10     0      seq
........agguucugugauacacuccgacuU.........................................    2      1      seq
........agguucugugauacacuccgacucU........................................    6      1      seq
........agguucugugauacacuccgacucAA.......................................    1      2      seq
........agguucugugauacacuccgacucAg.......................................    1      1      seq
.........Uguucugugauacacuccgacu..........................................    1      1      seq
.........gguucugugauacacuccgacu..........................................    2      0      seq
...........uucugugauacacucGgacu..........................................    1      1      seq
...........uucugugauacacuccgacu..........................................    6      0      seq
.............................cucgggcucuggagcagucag.......................    1      0      seq
..........................................CcaAucagugcaugacagaacuugg......    1      2      seq
...........................................cUAucagugcaugacagaacuugg......    1      2      seq
...........................................GaAucagugcaugacagaacuugg......    1      2      seq
...........................................cGAucagugcaugacagaacuugg......    4      2      seq
...........................................cUgucagugcaugacagaacuugg......    4      1      seq
...........................................caCucagugcaugacagaacuuggA.....    1      2      seq
...........................................cUgucagugcaugacagaacuugggAc...    1      2      seq
............................................UCucagugcaugacagaacuugg......    2      2      seq
............................................CUucagugcaugacagaacuugg......    1      2      seq
.............................................gucagugcaugacagaacuug.......    3      0      seq
.............................................gucagugcaugacagaacuGg.......    1      1      seq
.............................................gucagugcaugCcagaacuugg......    1      1      seq
.............................................gucagugcaugacagaacuugU......    5      1      seq
.............................................gucagugcaugacagCacuugg......    1      1      seq
.............................................gucaguUcaugacagaacuugg......    1      1      seq
.............................................gucagugcauAacagaacuugg......    1      1      seq
.............................................gucagugcCugacagaacuugg......    1      1      seq
.............................................gCcagugcaugacagaacuugg......    1      1      seq
.............................................gucagugAaugacagaacuugg......    1      1      seq
.............................................gucagugcaugaUagaacuugg......    1      1      seq
.............................................gucaAugcaugacagaacuugg......    1      1      seq
.............................................UucagugcaugacagaacuugU......    12     2      seq
.............................................guUagugcaugacagaacuugg......    1      1      seq
.............................................gucagugcaugacagaacuCgg......    1      1      seq
.............................................gucaUugcaugacagaacuugg......    3      1      seq
.............................................CucagGgcaugacagaacuugg......    1      2      seq
.............................................Cucagugcaugacagaacuugg......    17     1      seq
.............................................gucagugcaugacGgaacuugg......    1      1      seq
.............................................gucagugcaugacagaacuugg......    1737   0      seq
.............................................gucagugcGugacagaacuugg......    2      1      seq
.............................................UGcagugcaugacagaacuugg......    1      2      seq
.............................................gucaCugcaugacagaacuugg......    1      1      seq
.............................................Uucagugcaugacagaacuugg......    9      1      seq
.............................................gucagugcaugacagaacuugAU.....    1      2      seq
.............................................gucagugUaugacagaacuuggA.....    1      2      seq
.............................................gucagugcaugacagaacuuggA.....    251    1      seq
.............................................gucagugcaugacagGacuuggU.....    1      2      seq
.............................................UucagugcaugacagaacuuggA.....    1      2      seq
.............................................Cucagugcaugacagaacuuggg.....    1      1      seq
.............................................gucagugcaugacagaacuugAg.....    1      1      seq
.............................................gucagugcaugacagaGcuuggA.....    1      2      seq
.............................................gucagugcaugacagaacuuggU.....    155    1      seq
.............................................CucagugcaugacagaacuuggU.....    2      2      seq
.............................................gucagugcaugacGgaacuuggA.....    1      2      seq
.............................................CucagugcaugacagaacuuggA.....    7      2      seq
.............................................gucagugcaugacagaacuuggg.....    1      0      seq
.............................................gucagugcaugacagaacuuggC.....    1      1      seq
.............................................AucagugcaugacagaacuuggA.....    1      2      seq
.............................................Uucagugcaugacagaacuuggg.....    1      1      seq
.............................................gucagugcaugacagaacuuggUA....    5      2      seq
.............................................gucagugcaugacagaacuuggAA....    1      2      seq
.............................................gucagugcaugacagaacuuggAG....    2      2      seq
.............................................gucagugcaugacagaacuugggA....    2      1      seq
```

```
                                              mmu-miR-152-3p
       mmu-miR-152-5p
ccgggccuagguucugugauacacuccgacucgggcucuggagcagucagugcaugacagaacuugggcccgg

..............................................ucagugcaugacagaac..........   22    0    seq
..............................................ucagugcaugacagaacu.........   48    0    seq
..............................................ucagugcaugacagaaAA.........   1     2    seq
..............................................ucagugcaugacagaacGC........   1     2    seq
..............................................ucagugcaugacagaacuu........   300   0    seq
..............................................ucagugcaCgacagaacuA........   1     2    seq
..............................................ucagugcaugaUagaacuu........   1     1    seq
..............................................ucaguAcaugacagaacuu........   1     1    seq
..............................................ucagugcaugacagCacuu........   1     1    seq
..............................................ucagugcaugacagaacuug.......   300   0    seq
..............................................ucagugcaugaGagaacuug.......   1     1    seq
..............................................ucagugcaugacagaacuuC.......   1     1    seq
..............................................ucagugcauCacagaacuug.......   2     1    seq
..............................................ucagugcaugacagaacuGg.......   6     1    seq
..............................................ucagugcaugacagaacuuA.......   13    1    seq
..............................................ucagugcauUacagaacuug.......   1     1    seq
..............................................ucagCgcaugacagaacuug.......   1     1    seq
..............................................ucagugcaugacagaacuuU.......   14    1    seq
..............................................ucagugcaugacagaacuAA.......   2     2    seq
..............................................uUagugcauUacagaacuugg......   1     2    seq
..............................................Gcagugcaugacagaacuugg......   22    1    seq
..............................................ucagAgcaugacagaacuuAg......   1     2    seq
..............................................ucagugcaugaAagaacuugg......   11    1    seq
..............................................ucGgugcaugacagaacuugg......   57    1    seq
..............................................ucagugcaugacagaacCugU......   2     2    seq
..............................................ucaUugUaugacagaacuugg......   1     2    seq
..............................................ucagugcauUacagaacuugA......   1     2    seq
..............................................ucagugcaugacagGacuugg......   26    1    seq
..............................................ucagCUcaugacagaacuugg......   4     2    seq
..............................................ucCgugcaugacagaacuugg......   17    1    seq
..............................................ucagugcUugacagaacuugg......   9     1    seq
..............................................ucagugcaugacGgaacuugg......   75    1    seq
..............................................ucaUugcaugacagaacuugg......   100   1    seq
..............................................ucagugcaugacagaacGugg......   5     1    seq
..............................................ucagugcCugacagaacuugA......   1     2    seq
..............................................ucagugcauCaUagaacuugg......   1     2    seq
..............................................ucaguAcaugGcagaacuugg......   1     2    seq
..............................................ucagugcaugUcagaacuugg......   38    1    seq
..............................................ucagugcaugCcagaacuugU......   1     2    seq
..............................................ucaguUcaugacagaacuugg......   48    1    seq
..............................................ucaAugcaugacagaacuugg......   49    1    seq
..............................................Ccagugcaugacagaacuugg......   67    1    seq
..............................................ucagugcaugaUagaacuugg......   81    1    seq
..............................................uUagugcaugacagaacuugg......   50    1    seq
..............................................uAagugcaugacagaacuugg......   14    1    seq
..............................................GcagugcaugacagaacuugU......   2     2    seq
..............................................ucCgugcaugacagaacuCgg......   1     2    seq
..............................................ucagugcaugacagaacuGgA......   1     2    seq
..............................................ucagugcaugacUgaacuugg......   24    1    seq
..............................................ucaguCcaugacagaacuugU......   1     2    seq
..............................................ucagugcaugCcagaacuugg......   56    1    seq
..............................................ucaCugcaugacCgaacuugg......   1     2    seq
..............................................ucagugAaugacagaacuugg......   16    1    seq
..............................................ucagugcaugacagaacAugg......   7     1    seq
..............................................ucagugcaugacagaacuuUC......   3     2    seq
..............................................ucUgugcaugacagaacuugU......   1     2    seq
..............................................ucagugcGugacagaacuugg......   48    1    seq
..............................................ucagugcaugacagaacuuUU......   8     2    seq
..............................................AcagugUaugacagaacuugg......   1     2    seq
..............................................ucagugcaugacagaacuuUg......   28    1    seq
..............................................ucagugcaugacaCaacuugg......   24    1    seq
..............................................ucagugUaugacagaacuugg......   59    1    seq
..............................................ucagugcaugacagaCcuugA......   1     2    seq
..............................................ucagugcaugacagaCcuugg......   17    1    seq
..............................................ucaCugcaugacagaacuugg......   35    1    seq
..............................................ucagugcauUacagaacuuCg......   1     2    seq
..............................................ucagugcaugacagaacuuCg......   27    1    seq
..............................................ucagugcaugacagaGcuugg......   38    1    seq
..............................................ucagugcauCacagaacuugU......   1     2    seq
```

```
                                              mmu-miR-152-3p
       mmu-miR-152-5p
ccgggccuagguucugugauacacuccgacucgggcucuggagcagucagugcaugacagaacuugggcccgg

..............................................ucaguAcaugacagaGcuugg......   1        2         seq
..............................................ucagugcaugacagaUcuugg......   9        1         seq
..............................................ucagugcaCgacagaacuugU......   1        2         seq
..............................................ucaUugcaugacagaacuugA......   1        2         seq
..............................................ucagugcCugacagaacuugg......   26       1         seq
..............................................ucagugUaugacagaacuugU......   1        2         seq
..............................................ucagugcaugacagaacuuAU......   7        2         seq
..............................................ucaguCcaugacagaacuugg......   42       1         seq
..............................................ucagugcauAacagaacuugg......   49       1         seq
..............................................ucagugcaugGcagaacuugg......   49       1         seq
..............................................ucaCCgcaugacagaacuugg......   1        2         seq
..............................................ucagugcaugacagaacuugC......   69       1         seq
..............................................ucagugcaugacaAaacuugg......   15       1         seq
..............................................Acagugcaugacagaacuugg......   35       1         seq
..............................................ucagugcaugaGagaacuugg......   14       1         seq
..............................................ucagugcauUacagaacuugg......   82       1         seq
..............................................ucagugcaugacagCacuugg......   36       1         seq
..............................................ucagugcGugacagaacuugU......   1        2         seq
..............................................ucagugcaugacagaaUuugg......   50       1         seq
..............................................AcagugcaugacaAaacuugg......   1        2         seq
..............................................ucagCgcaugacagaacuugA......   1        2         seq
..............................................CcagugcaugacaAaacuugg......   1        2         seq
..............................................ucagugcaugacaAaacCugg......   1        2         seq
..............................................ucagugcaugacagaacuugU......   1728     1         seq
..............................................ucGgGgcaugacagaacuugg......   1        2         seq
..............................................ucagCgcaugacagaCcuugg......   1        2         seq
..............................................ucUgugcaugacagaacuugg......   9        1         seq
..............................................GcagugcaugacagaacuuUg......   1        2         seq
..............................................ucagugcaugacagaacuGgU......   1        2         seq
..............................................ucagugcaugacagaaAuugU......   1        2         seq
..............................................ucagugcaugacCgaacuugg......   31       1         seq
..............................................ucagugcaugacCgaacuugU......   1        2         seq
..............................................ucagugcaugacagaacuuAg......   49       1         seq
..............................................ucagugcaugacagaaAuugg......   10       1         seq
..............................................ucagugcauCacagaacuugg......   43       1         seq
..............................................ucagugcaugacagaacuAgU......   1        2         seq
..............................................ucagGgcaugacagaacuugg......   235      1         seq
..............................................ucaCugcaugacagaacuugU......   2        2         seq
..............................................ucagugcaAgacagaacuugU......   1        2         seq
..............................................ucagugcaGgacagaacuugg......   9        1         seq
..............................................ucagugcaugacagaaGuugg......   2        1         seq
..............................................ucagugcaugacagaacuugg......   234989   0         seq
..............................................ucUCugcaugacagaacuugg......   1        2         seq
..............................................ucagugcaugacagaacuuCU......   2        2         seq
..............................................ucagugcaugacagaacuAgg......   7        1         seq
..............................................CcagugcaugacagaacuugU......   1        2         seq
..............................................ucagugcaugacagaacCugg......   61       1         seq
..............................................ucagugcaugaUagaacuugA......   1        2         seq
..............................................ucagugcaugacagaacuugA......   1100     1         seq
..............................................ucagugGaugacagaacuugg......   17       1         seq
..............................................ucagugcaugacagaacuGgg......   10       1         seq
..............................................ucagugcaugacaCaacuugA......   1        2         seq
..............................................ucagugcaAgacagaacuugg......   16       1         seq
..............................................ucagugcaugacagaacuuAA......   14       2         seq
..............................................uGagugcaugacagaacuugg......   8        1         seq
..............................................ucagugAaugacagaacuugA......   1        2         seq
..............................................CcagugcaugacCgaacuugg......   1        2         seq
..............................................ucagugcaugacagUacuugg......   4        1         seq
..............................................ucagAgcaugacagaacuugg......   72       1         seq
..............................................ucagGgcaugacagaacuugU......   2        2         seq
..............................................ucagugcaugacagaacuCgg......   58       1         seq
..............................................ucagGgcauUacagaacuugg......   1        2         seq
..............................................ucaguAcaugacagaacuugg......   88       1         seq
..............................................ucagugcaCgacagaacuugg......   49       1         seq
..............................................ucagugcaugaUagaacuugU......   1        2         seq
..............................................ucagugcaugacaUaacuugg......   26       1         seq
..............................................ucagugcaugacagaacuCgU......   1        2         seq
..............................................ucagGgcaugacagaacuugA......   1        2         seq
..............................................ucagugcaugacagaacuAgA......   1        2         seq
```

mmu-miR-152-3p

mmu-miR-152-5p

ccgggccuagguucugugauacacuccgacucgggcucuggagcagucagugcaugacagaacuugggcccgg

....ucaCugcaugacagaaUuugg......	1	2	seq
....ucagCgcaugacagaacuugg......	156	1	seq
....ucagugcCugCcagaacuugg......	1	2	seq
....ucagugcaugacagGacuuggA.....	7	2	seq
....ucagugcaugUcagaacuuggg.....	1	1	seq
....GcagugcaugacagaacuuggA.....	5	2	seq
....uUagugcaugacagaacuuggU.....	5	2	seq
....ucagugcaugacagaacAuggU.....	1	2	seq
....ucaAugcaugacagaacuuggA.....	11	2	seq
....ucaguAcaugacagaacuuggU.....	6	2	seq
....uGagugcaugacagaacuuggU.....	1	2	seq
....ucagugcaugacagaGcuuggg.....	2	1	seq
....ucaUugcaugacagaacuuggA.....	15	2	seq
....ucagGgcaugacagaacuuggU.....	37	2	seq
....ucagugcaugacagaacuuCgU.....	6	2	seq
....ucagugcaugacagaaUuuggA.....	5	2	seq
....ucagugcaugacagaGcuuggA.....	3	2	seq
....ucagugcaugacagaacuugAg.....	8	1	seq
....ucagugcaugaGagaacuuggA.....	4	2	seq
....AcagugcaugacagaacuuggU.....	3	2	seq
....ucagugcGugacagaacuuggU.....	10	2	seq
....ucagugcaugacGgaacuuggU.....	10	2	seq
....ucagCgcaugacagaacuuggA.....	37	2	seq
....ucagugcaugacagaacuuCgA.....	7	2	seq
....ucagugcaCgacagaacuuggA.....	7	2	seq
....ucagugcGugacagaacuuggg.....	1	1	seq
....ucUgugcaugacagaacuuggA.....	1	2	seq
....ucagugcaugacagaUcuuggU.....	1	2	seq
....ucagugAaugacagaacuuggg.....	2	1	seq
....ucagugcaugCcagaacuuggA.....	9	2	seq
....uAagugcaugacagaacuuggg.....	1	1	seq
....ucagugGaugacagaacuuggU.....	2	2	seq
....ucagugcaCgacagaacuuggU.....	8	2	seq
....ucagugcaugacagaacuGggU.....	2	2	seq
....ucagugcaugacagaacuuggg.....	9572	0	seq
....ucagugGaugacagaacuuggg.....	1	1	seq
....ucagugcaugacagaacAuggA.....	1	2	seq
....ucagugcaugGcagaacuuggg.....	3	1	seq
....ucagugcaugacaAaacuuggU.....	2	2	seq
....ucagugcaugacagaCcuuggU.....	3	2	seq
....ucagugcaugacagaacuCggU.....	8	2	seq
....ucagugcaCgacagaacuuggg.....	1	1	seq
....ucagugcaugacagaacuugUA.....	49	2	seq
....ucagugcauUacagaacuuUgg.....	1	2	seq
....ucagugcaugacagaacuGggg.....	1	1	seq
....ucagugcaugacagaacCuggU.....	4	2	seq
....ucagugcaugacagCacuuggU.....	6	2	seq
....ucagugcaugaGagaacuuggU.....	5	2	seq
....ucagugcaugacagaacCuggA.....	10	2	seq
....ucagAgcaugacagaacuuggg.....	5	1	seq
....ucagCgcaugacagaacuuggU.....	22	2	seq
....ucagugcaGgacagaacuuggA.....	1	2	seq
....ucaguCcaugacagaacuuggg.....	2	1	seq
....ucaUugcaugacagaacuuggU.....	17	2	seq
....ucagugcaugacagaUcuuggA.....	3	2	seq
....ucagugcaugacagaacuGggA.....	1	2	seq
....ucagugcaugaUagaacuuggA.....	14	2	seq
....ucagugcaugacaCaacuuggU.....	3	2	seq
....ucagugcCugacagaacuuggA.....	2	2	seq
....ucagCgcaugacagaacuuggg.....	8	1	seq
....uUagugcaugacagaacuuggA.....	11	2	seq
....ucagAgcaugacagaacuuggU.....	12	2	seq
....ucagugcaugaGagaacuuggg.....	1	1	seq
....ucagugcauUacagaacuuggA.....	10	2	seq
....ucagugcaugacagaacuuAgA.....	7	2	seq
....ucagugUaugacagaacuuggA.....	19	2	seq
....ucagugcaugUcagaacuuggU.....	3	2	seq
....ucagugcaAgacagaacuuggA.....	1	2	seq
....ucagugcaugGcagaacuuggU.....	6	2	seq

```
                                              mmu-miR-152-3p
       mmu-miR-152-5p
ccgggccuagguucugugauacacuccgacucgggcucuggagcagucagugcaugacagaacuugggcccgg

..............................................ucagGcaugacagaacuuggg.....    6        1          seq
..............................................ucagugcaugacagUacuuggU.....    1        2          seq
..............................................ucagugcaugaAagaacuuggU.....    2        2          seq
..............................................ucagugcaugacagGacuuggU.....    3        2          seq
..............................................ucagugcaugacagaacuugCg.....    2        1          seq
..............................................ucagugcaugacagaacuugCU.....    3        2          seq
..............................................ucagugcaugaUagaacuuggg.....    4        1          seq
..............................................ucGgugcaugacagaacuuggU.....    6        2          seq
..............................................ucagugAaugacagaacuuggU.....    1        2          seq
..............................................ucaguUcaugacagaacuuggg.....    5        1          seq
..............................................ucaguUcaugacagaacuuggA.....    10       2          seq
..............................................ucagugcaugacagaacuugAU.....    8        2          seq
..............................................ucagugAaugacagaacuuggA.....    2        2          seq
..............................................ucCgugcaugacagaacuuggg.....    1        1          seq
..............................................ucagugcaugaAagaacuuggA.....    2        2          seq
..............................................ucagugcaugacagaaAuuggA.....    2        2          seq
..............................................ucagugcaugacagaCcuuggg.....    1        1          seq
..............................................ucagugcaugacagaacCuggg.....    4        1          seq
..............................................ucagugcGugacagaacuuggA.....    8        2          seq
..............................................ucagugcaugUcagaacuuggA.....    8        2          seq
..............................................ucagugGaugacagaacuuggA.....    1        2          seq
..............................................ucaguAcaugacagaacuuggA.....    13       2          seq
..............................................ucagugcaugacUgaacuuggA.....    3        2          seq
..............................................ucagugcaugacCgaacuuggA.....    4        2          seq
..............................................ucagugcaugaAagaacuuggC.....    1        2          seq
..............................................ucagugcauUacagaacuuggg.....    3        1          seq
..............................................ucagugcaugacagaacuAggU.....    3        2          seq
..............................................ucagugUaugacagaacuuggU.....    3        2          seq
..............................................ucaCugcaugacagaacuuggA.....    4        2          seq
..............................................ucGgugcaugacagaacuuggA.....    10       2          seq
..............................................ucagugcaugacUgaacuuggg.....    1        1          seq
..............................................ucUgugcaugacagaacuuggg.....    2        1          seq
..............................................ucagugcaugacagaacAuggg.....    1        1          seq
..............................................ucagugcaugacagaacuuCgg.....    3        1          seq
..............................................AcagugcaugacagaacuuggA.....    6        2          seq
..............................................ucagugcaugacagaacuuAgg.....    3        1          seq
..............................................ucagugcaugCcagaacuuggU.....    3        2          seq
..............................................ucagugcaugacagaaUuuggU.....    1        2          seq
..............................................ucagugcaugacagaacuugAA.....    29       2          seq
..............................................ucagugcaugacagaacuugAC.....    3        2          seq
..............................................ucagugcauAacagaacuuggA.....    7        2          seq
..............................................ucagugcaugacagaacuuUgg.....    5        1          seq
..............................................ucagugcaugacagaacuuAgU.....    5        2          seq
..............................................ucagugcUugacagaacuuggA.....    3        2          seq
..............................................ucagugcaugacagaacuugUU.....    66       2          seq
..............................................ucagugcaugacaAaacuuggA.....    5        2          seq
..............................................ucagugcaugacaUaacuuggU.....    2        2          seq
..............................................ucagugcUugacagaacuuggU.....    1        2          seq
..............................................ucaguCcaugacagaacuuggA.....    7        2          seq
..............................................ucagugcaugacagCacuuggA.....    1        2          seq
..............................................ucagugcaugacagaGcuuggU.....    8        2          seq
..............................................ucagugcauAacagaacuuggU.....    8        2          seq
..............................................ucagugcaugacagaacGuggA.....    2        2          seq
..............................................ucaguCcaugacagaacuuggU.....    5        2          seq
..............................................ucGgugcaugacagaacuuggg.....    3        1          seq
..............................................ucagugcaugacaUaacuuggA.....    3        2          seq
..............................................ucagugcaugacagaacuuggC.....    236      1          seq
..............................................ucCgugcaugacagaacuuggU.....    4        2          seq
..............................................uUagugcaugacagaacuuggg.....    1        1          seq
..............................................uAagugcaugacagaacuuggA.....    1        2          seq
..............................................ucagugcaugGcagaacuuggA.....    7        2          seq
..............................................ucagugcaugacagaacuAggA.....    1        2          seq
..............................................ucagugcaugCcagaacuuggg.....    1        1          seq
..............................................ucaAugcaugacagaacuuggU.....    11       2          seq
..............................................ucagugcaugacagaacuuggA.....    38057    1          seq
..............................................ucagugcaugacagaacuuUgA.....    10       2          seq
..............................................ucCgugcaugacagaacuuggA.....    4        2          seq
..............................................ucaCugcaugacagaacuuggg.....    2        1          seq
..............................................ucagugcaugacagaaAuuggU.....    4        2          seq
```

```
                                              mmu-miR-152-3p
       mmu-miR-152-5p
ccgggccuagguucugugauacacuccgacucgggcucuggagcagucagugcaugacagaacuugggcccgg

..............................................ucaguUcaugacagaacuuggU.....   14      2   seq
..............................................ucagugcaugacUgaacuuggU.....   2       2   seq
..............................................GcagugcaugacagaacuuggU.....   7       2   seq
..............................................ucagugcaugacagaacuuggU.....   30802   1   seq
..............................................ucaguAcaugacagaacuuggg.....   4       1   seq
..............................................ucagGgcaugacagaacuuggA.....   34      2   seq
..............................................ucagugUaugacagaacuuggg.....   2       1   seq
..............................................ucagugcaugacGgaacuuggA.....   12      2   seq
..............................................ucagugcaugacagaacuCggA.....   14      2   seq
..............................................ucagugcaugacagaacuCggg.....   6       1   seq
..............................................ucagugcaugacagaaUuuggg.....   1       1   seq
..............................................ucagugcCugacagaacuuggU.....   2       2   seq
..............................................ucagugcauUacagaacuuggU.....   19      2   seq
..............................................uAagugcaugacagaacuuggU.....   4       2   seq
..............................................ucagugcaugacCgaacuuggU.....   1       2   seq
..............................................ucagugcauCacagaacuuggA.....   3       2   seq
..............................................ucaCugcaugacagaacuuggU.....   4       2   seq
..............................................ucaAugcaugacagaacuuggg.....   2       1   seq
..............................................ucagugcaugacaCaacuuggA.....   3       2   seq
..............................................ucagGgcaugacagaacuuggC.....   1       2   seq
..............................................ucagugcaugaAagaacuuggg.....   2       1   seq
..............................................ucagugcCugacagaacuuggg.....   1       1   seq
..............................................ucagugcaugacagaacuugCA.....   10      2   seq
..............................................ucagugcaugacagaCcuuggA.....   3       2   seq
..............................................ucagugcaugacaCaacuuggg.....   1       1   seq
..............................................ucaUugcaugacagaacuuggg.....   7       1   seq
..............................................Ccagugcaugacagaacuuggg.....   1       1   seq
..............................................ucagugcauAacagaacuuggg.....   1       1   seq
..............................................ucagugcauCacagaacuuggg.....   2       1   seq
..............................................uGagugcaugacagaacuuggA.....   3       2   seq
..............................................ucUgugcaugacagaacuuggU.....   1       2   seq
..............................................ucaAugcaugacagaacuuggC.....   2       2   seq
..............................................ucagugcaugacagaacuugUg.....   2       1   seq
..............................................ucagugcaugaUagaacuuggU.....   19      2   seq
..............................................CcagugcaugacagaacuuggU.....   9       2   seq
..............................................CcagugcaugacagaacuuggA.....   16      2   seq
..............................................ucagugcaugacCgaacuuggg.....   1       1   seq
..............................................ucagAgcaugacagaacuuggA.....   13      2   seq
..............................................ucaUugcaugacagaacuugggA....   1       2   seq
..............................................ucUgugcaugacagaacuugggA....   1       2   seq
..............................................ucagugcaugacGgaacuugggA....   1       2   seq
..............................................ucagugcaugacagaacuuggCc....   1       1   seq
..............................................ucagGgcaugacagaacuugggA....   1       2   seq
..............................................ucagugcaugacagaacuugUUc....   1       2   seq
..............................................ucagugcaugacagaacuuggUU....   1860    2   seq
..............................................ucagugcaugacagaacCugggA....   2       2   seq
..............................................ucagugcaugacagaCcuugggA....   3       2   seq
..............................................ucaUugcaugacagaacuugggU....   2       2   seq
..............................................ucagugGaugacagaacuugggA....   1       2   seq
..............................................ucagugcaugacagaacuugAgA....   3       2   seq
..............................................CcagugcaugacagaacuugggA....   1       2   seq
..............................................ucagugcaugacagaacuuUggG....   1       2   seq
..............................................ucagugcaugacagaacuugCgU....   1       2   seq
..............................................ucagugcaugacagaaUuugggA....   1       2   seq
..............................................ucagugcaugacagaacuugggA....   2415    1   seq
..............................................ucagugcCugacagaacuugggA....   1       2   seq
..............................................ucaguCcaugacagaacuugggA....   1       2   seq
..............................................ucagugcaugacagaacuuggAc....   93      1   seq
..............................................ucagugcaugacagaacuuggUG....   61      2   seq
..............................................ucagugcaugacagGacuugggA....   1       2   seq
..............................................ucagugcaugacCgaacuugggA....   1       2   seq
..............................................ucagugcaugacagaacuCgggA....   2       2   seq
..............................................ucagugcaugacagaacuuggAA....   1746    2   seq
..............................................ucagugcaugacagaacuuUggA....   4       2   seq
..............................................ucagugcaugacagaacuuggAU....   719     2   seq
..............................................ucagAgcaugacagaacuugggU....   1       2   seq
..............................................ucagugcaugaUagaacuugggU....   1       2   seq
..............................................ucagugcaugacagaacuugggU....   1389    1   seq
..............................................ucagugcaugacagaacuuggCG....   2       2   seq
```

```
                                              mmu-miR-152-3p
       mmu-miR-152-5p
ccgggccuagguucugugauacacuccgacucgggcucuggagcagucagugcaugacagaacuugggcccgg

..............................................ucagugcaugacagaGcuugggA....   2      2     seq
..............................................ucagugcaugacagaacuugggc....   12     0     seq
..............................................ucagugcaugacagaacuuggUA....   2189   2     seq
..............................................ucagugcaugUcagaacuugggA....   1      2     seq
..............................................ucGgugcaugacagaacuugggA....   1      2     seq
..............................................ucaguAcaugacagaacuugggU....   2      2     seq
..............................................ucagugcauUacagaacuugggA....   3      2     seq
..............................................ucagugcaugacagaacuugggG....   11     1     seq
..............................................ucagugUaugacagaacuugggU....   1      2     seq
..............................................ucagugcauCacagaacuugggU....   1      2     seq
..............................................ucagugUaugacagaacuugggA....   1      2     seq
..............................................ucagugcaugacagaacuugAAc....   1      2     seq
..............................................ucagugcaCgacagaacuugggA....   2      2     seq
..............................................ucagugcaugGcagaacuugggU....   2      2     seq
..............................................ucagugcaugacagaacuuUggU....   2      2     seq
..............................................ucaAugcaugacagaacuugggA....   1      2     seq
..............................................ucagCgcaugacagaacuugggA....   3      2     seq
..............................................ucagugcaugacagCacuugggA....   1      2     seq
..............................................ucagugcaugacagaacuuCggU....   1      2     seq
..............................................ucagugcaugacagaacuuggAG....   318    2     seq
..............................................ucaguAcaugacagaacuugggA....   1      2     seq
..............................................ucagugcaugacagaacuuggCU....   16     2     seq
..............................................ucagugcauUacagaacuugggU....   2      2     seq
..............................................ucagugcaugacagaacuuggUc....   10     1     seq
..............................................ucagugcaugacagaacuuggCA....   25     2     seq
..............................................ucagugcaugacagaacuuggAcU...   3      2     seq
..............................................ucagugcaugacagaacuuggUcU...   14     2     seq
..............................................ucagugcaugacagaacuugggAG...   65     2     seq
..............................................ucagugcaugacagaacuugggUU...   158    2     seq
..............................................ucagugcaugacagaacuuggAUc...   6      2     seq
..............................................ucagugcaugacagaacuugggcA...   3      1     seq
..............................................ucagugcaugacagaacuuggAcG...   2      2     seq
..............................................ucagugcaugacagaacuuggAcA...   16     2     seq
..............................................ucagugcaugacagaacuugggGA...   3      2     seq
..............................................ucagugcaugacagaacuuggUUc...   8      2     seq
..............................................ucagugcaugacagaacuuggUGc...   1      2     seq
..............................................ucagugcaugacagaacuugggUG...   3      2     seq
..............................................ucagugcaugacagaacuugggAA...   176    2     seq
..............................................ucagugcaugacagaacuuggUAc...   52     2     seq
..............................................ucagugcaugacagaacuugggUA...   114    2     seq
..............................................ucagugcaugacagaacuugggcU...   2      1     seq
..............................................ucagugcaugacagaacuuggAGc...   4      2     seq
..............................................ucagugcaugacagaacuuggAAc...   83     2     seq
..............................................ucagugcaugacagaacuuggUcA...   1      2     seq
..............................................ucagugcaugacagaacuugggAc...   17     1     seq
..............................................ucagugcaugacagaacuugggAU...   239    2     seq
..............................................ucagugcaugacagaacuugggUcA..   1      2     seq
..............................................ucagugcaugacagaacuugggcAA..   1      2     seq
..............................................ucagugcaugacagaacuugggAcA..   2      2     seq
..............................................ucagugcaugacagaacuugggcAU..   1      2     seq
..............................................ucagugcaugacagaacuugggAAc..   9      2     seq
..............................................ucagugcaugacagaacuugggAcU..   3      2     seq
..............................................ucagugcaugacagaacuugggUAc..   5      2     seq
...............................................cagugcaugacagaacu.........   1      0     seq
...............................................cagugcaugacagaacuu........   11     0     seq
...............................................cagugcaugacagaacuug.......   9      0     seq
...............................................cagugcaugacCgaacuugg......   1      1     seq
...............................................cagugcaugCAagaacuugg......   1      2     seq
...............................................cagGgcaugCcagaacuugg......   1      2     seq
...............................................cagugcaugacagaacuugU......   34     1     seq
...............................................cagugcaGgacagaacuugg......   1      1     seq
...............................................cagugcaugacagaacuugA......   14     1     seq
...............................................cagGgcaugacagaacuugU......   1      2     seq
...............................................cagugcaugacagaacuuCg......   1      1     seq
...............................................Aagugcaugacagaacuugg......   1      1     seq
...............................................cagugcCugacagaacuugg......   1      1     seq
...............................................cagugcaugUcagaacuugg......   2      1     seq
...............................................cagugcaugacagaacuGgg......   1      1     seq
...............................................Gagugcaugacagaacuugg......   1      1     seq
```

mmu-miR-152-3p

mmu-miR-152-5p

ccgggccuagguucugugauacacuccgacucgggcucuggagcagucagugcaugacagaacuugggcccgg

....cagugcGugacagaacuugg......	1	1	seq
....cagugcaugaUagaacuugg......	1	1	seq
....cagugcaCgacagaacuugg......	1	1	seq
....cagugcauCacagaacuugA......	1	2	seq
....cagugcaAgacagaacuugg......	1	1	seq
....cagugcaugacagaacuCgg......	1	1	seq
....cagugcaugacagGacuugg......	2	1	seq
....cagugcaugacGgaacuugg......	3	1	seq
....cagugcaugacagaacuuAU......	1	2	seq
....cagGgcaugacagaacuugg......	23	1	seq
....cagugcaugacagaCcuugg......	1	1	seq
....cagugcaugaAagaacuugg......	4	1	seq
....cagugcaugacagaaUuugg......	5	1	seq
....Uagugcaugacagaacuugg......	3	1	seq
....cGgugcaugacagaacuugg......	2	1	seq
....cagugcaugacaUaacuugg......	1	1	seq
....cagugcaugacagaacuugg......	4412	0	seq
....cagugcaugacagaacuugC......	1	1	seq
....cagugUaugacagaacuugg......	1	1	seq
....caUugcaugacagaacuugg......	3	1	seq
....caCugcaugacagaacuugg......	4	1	seq
....cagugcaugCcagaacuugg......	17	1	seq
....cagugcaCgacagaacuugU......	1	2	seq
....caguCcaugacagaacuugg......	1	1	seq
....cagugcaugacagaacuuUg......	1	1	seq
....cagGgcaugacagaacuuggU.....	7	2	seq
....caguCcaugacagaacuuggU.....	1	2	seq
....cagugcaugacagaacuugCA.....	1	2	seq
....cagugcaugacagaacuugAA.....	1	2	seq
....cagugcaugacaUaacuuggA.....	4	2	seq
....cagugcaugacaUaacuuggg.....	1	1	seq
....cagugcauUacagaacuuggA.....	2	2	seq
....cagugcaugacagaacuuggg.....	174	0	seq
....cagugcaugacaUaacuuggU.....	4	2	seq
....cagugcaugacGgaacuuggU.....	2	2	seq
....cagugcaugacagaacuuggA.....	853	1	seq
....cagugcUugacagaacuuggU.....	1	2	seq
....cagugcaugacagaacuuggC.....	5	1	seq
....cagugcaugCcagaacuuggU.....	10	2	seq
....GagugcaugacagaacuuggU.....	1	2	seq
....cagugcaugCcagaacuuggg.....	2	1	seq
....cagugcaugacaAaacuuggU.....	1	2	seq
....cagugcaugacagaacuugUU.....	1	2	seq
....AagugcaugacagaacuuggA.....	5	2	seq
....cagugcaugacagaacuuUgg.....	1	1	seq
....cagugcaugCcagaacuuggA.....	8	2	seq
....cGgugcaugacagaacuuggU.....	1	2	seq
....cagugcaugacagaaAuuggU.....	1	2	seq
....cagGgcaugacagaacuuggg.....	2	1	seq
....cagugUaugacagaacuuggU.....	1	2	seq
....cagugcaugacagaacuugUA.....	1	2	seq
....caUugcaugacagaacuuggA.....	1	2	seq
....cagugcaugaUagaacuuggU.....	1	2	seq
....cagugcaugacagaacuuggU.....	719	1	seq
....cagGgcaugacagaacuuggA.....	8	2	seq
....cagugcaugacagaaUuuggg.....	1	1	seq
....cagugcauUacagaacuugggA....	1	2	seq
....cagugcaugacagaacuuggUU....	43	2	seq
....cagugcaugacagaacuuggCA....	1	2	seq
....cagugcaugacagaacuuggUA....	33	2	seq
....cagugcaugacagaacuuggAA....	51	2	seq
....cagugcaugacagaacuugggU....	33	1	seq
....cagugcaugacagaacuugggA....	62	1	seq
....cagugcaugacagaacuuggUG....	1	2	seq
....cagugcaugacagaacuuggAU....	24	2	seq
....cagugcaugacagaacuugggG....	1	1	seq
....cagugcaugacagaacuuggAG....	6	2	seq
....cagugcaugacagaacuuggAc....	2	1	seq
....cagugcaugacagaacuugggAG...	1	2	seq

mmu-miR-152-3p

mmu-miR-152-5p

ccgggccuagguucugugauacacuccgacucgggcucuggagcagucagugcaugacagaacuugggcccgg

....cagugcaugacagaacuugggAA...	6	2	seq
....cagugcaugacagaacuuggUAc...	1	2	seq
....cagugcaugacagaacuugggUA...	3	2	seq
....cagugcaugacagaacuugggUU...	2	2	seq
....cagugcaugacagaacuuggAAc...	3	2	seq
....cagugcaugacagaacuugggAU...	4	2	seq
....agugcaugacagaacuu........	8	0	seq
....agugcaugacagaacuug.......	2	0	seq
....Cgugcaugacagaacuugg......	1	1	seq
....agugcaugacagaaUuugg......	2	1	seq
....agugUaugacagaacuugg......	1	1	seq
....agugcaugacagGacuugg......	1	1	seq
....aAugcaugacagaacuugg......	2	1	seq
....agugcaugaUagaacuugg......	2	1	seq
....agGgcaugacagaacuugg......	8	1	seq
....agugcaugacagaacuugA......	8	1	seq
....agugcaugacagaacuCgg......	1	1	seq
....agugcaugaGagaacuugg......	1	1	seq
....agCgcaugacagaacuugg......	5	1	seq
....agugcaugacagUacuugg......	1	1	seq
....agugcaugacagaacuugU......	34	1	seq
....agugcauAacagaacuugg......	3	1	seq
....aguCcaugacagaacuugg......	2	1	seq
....agugcaugacagaCcuugg......	1	1	seq
....agugcauUacagaacuugg......	1	1	seq
....Ggugcaugacagaacuugg......	2	1	seq
....aUugcaugacagaacuugg......	5	1	seq
....agugcaugGcagaacuugg......	3	1	seq
....UCugcaugacagaacuugg......	17	2	seq
....agugcauCacagaacuugg......	3	1	seq
....agugcaugacGgaacuugg......	1	1	seq
....agugcaugacagaaAuugA......	1	2	seq
....agugcaugacagaacuuUg......	1	1	seq
....agugcaugacagaacuugg......	3131	0	seq
....agugcaCgacagaacuugg......	1	1	seq
....aguUcaugacagaacuugg......	1	1	seq
....agugcaugacGgaacuuggU.....	3	2	seq
....agGgcaugacagaacuuggU.....	1	2	seq
....UCugcaugacagaacuuggg.....	1	2	seq
....agugcaugacagaacuuggg.....	124	0	seq
....agugcaugacagaacuuggA.....	617	1	seq
....agugcaugacaCaacuuggA.....	1	2	seq
....agugcaugacagaacAuggA.....	1	2	seq
....agugcaugacagaacuuggU.....	427	1	seq
....agugcaugacagaacuugAU.....	1	2	seq
....agugcaugaUagaacuuggU.....	1	2	seq
....agugcaugacagCacuuggg.....	1	1	seq
....agugcaugacagGacuuggA.....	1	2	seq
....aAugcaugacagaacuuggU.....	1	2	seq
....agugcaugacagaacuuAgg.....	1	1	seq
....agugcaugacagaacuugUU.....	1	2	seq
....agGgcaugacagaacuuggA.....	1	2	seq
....agugcaugacagaacuugggA....	48	1	seq
....agugcaugacagaacuuggAc....	2	1	seq
....agugcaugacagaacuuggCA....	1	2	seq
....agugcaugacagaacuuggAA....	20	2	seq
....agugcaugacagaacuugggU....	17	1	seq
....agugcaugacagaacuuggAU....	9	2	seq
....agugcaugacagaacuuggAG....	2	2	seq
....agugcaugacagaacuuggUU....	25	2	seq
....agugcaugacagaacuuggUA....	22	2	seq
....agugcaugacagaacuugggUU...	3	2	seq
....agugcaugacagaacuugggAG...	2	2	seq
....agugcaugacagaacuugggUA...	4	2	seq
....agugcaugacagaacuugggAU...	4	2	seq
....agugcaugacagaacuuggUUc...	1	2	seq
....agugcaugacagaacuugggAGc..	2	2	seq
....agugcaugacagaacuugggAcA..	1	2	seq
....gugcaugacagaacuug.......	3	0	seq

```
                                              mmu-miR-152-3p
       mmu-miR-152-5p
ccgggccuagguucugugauacacuccgacucgggcucuggagcagucagugcaugacagaacuugggcccgg

.................................................gugcaugacagaacuugA......    3    1    seq
.................................................gugcaugacagaCcuugg......    1    1    seq
.................................................Uugcaugacagaacuugg......    1    1    seq
.................................................gugcaugacagaacuugg......    80   0    seq
.................................................gugcaugacagaacuuggA.....    19   1    seq
.................................................gugcaugacagaacuuggg.....    2    0    seq
.................................................gugcaugacagaacuuggU.....    10   1    seq
.................................................UugcaugacagaacuuggU.....    1    2    seq
.................................................gugGaugacagaacuuggA.....    1    2    seq
.................................................gugcaugacagaacuuggAA....    3    2    seq
.................................................gugcaugacagaacuuggUU....    1    2    seq
.................................................gugcaugacagaacuugggUcA..    2    2    seq
..................................................uCcaugacagaacuugg......    2    1    seq
..................................................ugcaugacagaacuugg......    33   0    seq
..................................................ugcaugacagUacuugg......    1    1    seq
..................................................ugcaugacagaacuuggA.....    6    1    seq
..................................................ugcaugacagaacuuggg.....    1    0    seq
..................................................ugcaugacagaacuuggU.....    4    1    seq
..................................................ugcaugacagaacuugggU....    1    1    seq
..................................................ugcaugacagaacuugggAA...    1    2    seq
...................................................gcaugacagaacuuggU.....    22   1    seq
...................................................gcaugacagaacuuggA.....    18   1    seq
...................................................UcaugacagaacuuggU.....    1    2    seq
...................................................gcaugacagaacuuggg.....    2    0    seq
...................................................gcaugacagaacuuggAU....    1    2    seq
...................................................gcaugacagaacuuggAA....    4    2    seq
...................................................gcaugacagaacuugggA....    1    1    seq
...................................................gcaugacagaacuugggU....    2    1    seq
....................................................caugacagaacuuggUA....    1    2    seq
.....................................................auAaUagaacuugggcc...    2    2    seq
```
