# Supplementary material for: mirPRo–a novel standalone program for differential expression and variation analysis of miRNAs
Source: Sci Rep. 2015 Oct 5;5:14617. doi: 10.1038/srep14617 (PMC4592965; doi:10.1038/srep14617)
Supplement: Supplementary Data 12-21 [file srep14617-s25.zip › Supplementary Data 14.pdf]

miRBase precursor : mmu-mir-152
Total read count : 257277
mmu-miR-152-5p read count : 744
mmu-miR-152-3p read count : 256515
remaining reads : 18

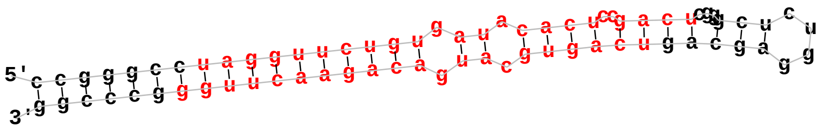

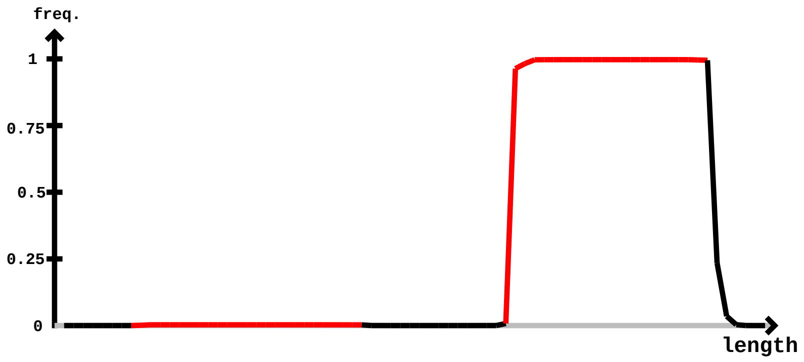


```
                                                mmu-miR-152-3p
          mmu-miR-152-5p
5'- ccgggccuagguucugugauacacuccgacucgggcucuggagcagucagugcaugacagaacuugggcccgg   -3'    exp
    (((((((((((((((((.((.((((..((((...(((....))))))))))).)).)))))))))))))))))   reads      mm         sample
    .......uagguucugugauacacu................................................   3          0          seq
    .......uagguucugugauacacuc...............................................   4          0          seq
    .......uagguucugugauacacucc..............................................   3          0          seq
    .......uagguucugugauacacuccg.............................................   7          0          seq
    .......uagguucugugauacacuccga............................................   17         0          seq
    .......uagguucugugauacacuccgU............................................   4          1          seq
    .......uagguucugugauacacuccgUU...........................................   1          2          seq
    .......uagguucugugauacacuUcgac...........................................   1          1          seq
    .......uagguucugugauacacuccgac...........................................   9          0          seq
    .......uagguucugugauacacuccgaA...........................................   2          1          seq
    .......uagguucugugauacacuccgaAG..........................................   2          2          seq
    .......uagguucugugauacacuccgacG..........................................   2          1          seq
    .......uagguucugugauacacuccgacu..........................................   239        0          seq
    .......uagguucugugauacacuccgacA..........................................   1          1          seq
    .......uagUuucugugauacacuccgacu..........................................   1          1          seq
    .......uagguucugugUuacacuccgacu..........................................   1          1          seq
    .......uagguucugugauacacuUcgacuU.........................................   2          2          seq
    .......uagguucugugauacacuccgacuU.........................................   1          1          seq
    ........agguucugugauacacu................................................   1          0          seq
    ........agguucuUugauacacucc..............................................   1          1          seq
    ........agguucugugauacacuccg.............................................   2          0          seq
    ........agguucugugauacacuccga............................................   7          0          seq
    ........agguucugugauacacuccgac...........................................   11         0          seq
    ........agguucugugauacacuccgacu..........................................   377        0          seq
    ........agguucugCgauacacuccgacu..........................................   1          1          seq
    ........agguucugugauacaUuccgacu..........................................   1          1          seq
    ........agguucugugauacacuGcgacu..........................................   1          1          seq
    ........aggGucugugauacacuccgacu..........................................   2          1          seq
    ........agguucugugUuacacuccgacuc.........................................   1          1          seq
    ........agguucugugauacacuccgacuU.........................................   2          1          seq
    ........agguucugugauacacuccgacAU.........................................   1          2          seq
    ........agguucugugauacacuccgacuA.........................................   1          1          seq
    ........agguucugugauacacuccgacuc.........................................   13         0          seq
    ........agguucugugauacacuccgacucU........................................   13         1          seq
```

```
                                                   mmu-miR-152-3p
       mmu-miR-152-5p
ccgggccuagguucugugauacacuccgacucgggcucuggagcagucagugcaugacagaacuugggcccgg

........agguucugugauacacuccgacuAgU.......................................    2       2       seq
.........gguucugugauacacuccgacu..........................................    2       0       seq
...........uucugugauacacuccgacu..........................................    3       0       seq
............ucugugauacacuccga............................................    2       0       seq
..........................................gcagucagugcaugacagaacuCU.......    1       2       seq
..........................................Ccagucagugcaugacagaacuugg......    1       1       seq
...........................................cCUucagugcaugacagaacuugg......    1       2       seq
...........................................cUAucagugcaugacagaacuugg......    1       2       seq
...........................................cGUucagugcaugacagaacuugg......    2       2       seq
...........................................cagucagugcaugacagaacuugg......    3       0       seq
...........................................cUgucagugcaugacagaacuugg......    1       1       seq
...........................................UUgucagugcaugacagaacuugg......    1       2       seq
...........................................cCgucagugcaugacagaacuugg......    1       1       seq
...........................................cGAucagugcaugacagaacuugg......    1       2       seq
...........................................cCAucagugcaugacagaacuugg......    1       2       seq
...........................................cCCucagugcaugacagaacuugg......    1       2       seq
...........................................caCucagugcaugacagaacuuggA.....    1       2       seq
...........................................cCgucagugcaugacagaacuuggg.....    1       1       seq
...........................................cUgucagugcaugacagaacuuggU.....    1       2       seq
............................................Cgucagugcaugacagaacuugg......    1       1       seq
............................................agucagugcaugacagaacuugg......    2       0       seq
............................................UCucagugcaugacagaacuugg......    1       2       seq
............................................UUucagugcaugacagaacuugg......    1       2       seq
............................................CgucagugcaugacagaacuuggA.....    1       2       seq
............................................UgucagugcaugacagaacuuggU.....    1       2       seq
.............................................Uucagugcaugacagaacuug.......    1       1       seq
.............................................gucagugcaugacagaacuug.......    6       0       seq
.............................................gucagugcaugacagaacuGgg......    1       1       seq
.............................................gucagugcaugacagaacuugg......    1620    0       seq
.............................................gCcagugcaugacagaacuugg......    2       1       seq
.............................................gucGgugcaugacagaacuugg......    1       1       seq
.............................................gucagugcaugacagaaAuugg......    1       1       seq
.............................................gucagAgcaugacagaacuugg......    1       1       seq
.............................................Uucagugcaugacagaacuugg......    6       1       seq
.............................................gucagugcaugacaCaacuugg......    1       1       seq
.............................................gucagGgcaugacagaacuGgg......    1       2       seq
.............................................gucagGgcaugacagaacuugg......    1       1       seq
.............................................gucagugcaugacagaacuugU......    4       1       seq
.............................................gucagugcaugacagaCcuugg......    1       1       seq
.............................................gucagugUaugacagaacuugg......    1       1       seq
.............................................Cucagugcaugacagaacuugg......    11      1       seq
.............................................gucaguAcaugacagaacuugg......    1       1       seq
.............................................gucagugcaugacaUaacuugg......    1       1       seq
.............................................guGagugcaugacagaacuugg......    1       1       seq
.............................................gucagugcaCgacagaacuugg......    2       1       seq
.............................................guUagugcaugacagaacuugg......    1       1       seq
.............................................gucagugcaugacagaacuuggC.....    3       1       seq
.............................................CucagugcaugacagaacuuggU.....    1       2       seq
.............................................gucagugcaugacagaacuuggA.....    191     1       seq
.............................................gucaguCcaugacagaacuuggA.....    1       2       seq
.............................................gucagugcauAacagaacuuggA.....    1       2       seq
.............................................UucagugcaugacagaacuuggU.....    1       2       seq
.............................................gucagugcaugacagaacuuUgC.....    1       2       seq
.............................................gucagugcaugacagaacuuggU.....    112     1       seq
.............................................UucagugcaugacagaacuuggA.....    1       2       seq
.............................................gucagugcaCgacagaacuuggA.....    1       2       seq
.............................................Cucagugcaugacagaacuuggg.....    1       1       seq
.............................................gucagugcauAacagaacuuggU.....    1       2       seq
.............................................gucagugcaugacagaCcuuggU.....    1       2       seq
.............................................gucagugcaugacagaacuuggg.....    3       0       seq
.............................................CucagugcaugacagaacuuggA.....    2       2       seq
.............................................gucagugcGugacagaacuuggA.....    1       2       seq
.............................................gucagugcaugacagaacuuggUU....    1       2       seq
.............................................gucagugcaugacagaacuuggAG....    1       2       seq
.............................................CucagugcaugacagaacuuggAc....    1       2       seq
.............................................gucagugcaugacagaacuuggUA....    1       2       seq
.............................................gucagugcaugacagaacuugggU....    2       1       seq
.............................................gucagugcaugacagaacuuggAAc...    1       2       seq
..............................................ucagugcaugacagaac..........    10      0       seq
```

```
                                              mmu-miR-152-3p
       mmu-miR-152-5p
ccgggccuagguucugugauacacuccgacucgggcucuggagcagucagugcaugacagaacuugggcccgg

..............................................ucagugcaCgacagaac..........    1      1      seq
..............................................ucagugcaugacagaacu.........    26     0      seq
..............................................ucagugcaugacagaacuu........    257    0      seq
..............................................ucGgugcaugacagaacuug.......    1      1      seq
..............................................ucagugcaugacagaacuGg.......    1      1      seq
..............................................ucagugcaugacagaacuuA.......    18     1      seq
..............................................ucagugcaugacagaacuug.......    223    0      seq
..............................................ucagugcaugacagaacuuC.......    1      1      seq
..............................................ucagugcaugacagaacuAA.......    2      2      seq
..............................................ucagugcaugacagaacuuU.......    7      1      seq
..............................................ucagugcaCgacagaacuug.......    1      1      seq
..............................................ucagugcaugacagGacuug.......    1      1      seq
..............................................ucagAgcaugacagaacuug.......    1      1      seq
..............................................ucagugcaugacagaacuAgU......    1      2      seq
..............................................ucagugcaugacagaacuAgg......    4      1      seq
..............................................uAagugcaugacagaacuugg......    15     1      seq
..............................................ucagugcaugacagaacuugA......    742    1      seq
..............................................ucGgugcaugacagaacuugg......    47     1      seq
..............................................ucagugcauCacagaacuugU......    2      2      seq
..............................................ucagugcauUacagaacuugg......    93     1      seq
..............................................ucaUugcaugacagaacuugU......    1      2      seq
..............................................ucagugcaugacGgaacuugg......    45     1      seq
..............................................ucagugcaugaAagaacuugg......    14     1      seq
..............................................ucagugcaugacagaacuuUU......    11     2      seq
..............................................ucagugUaugacagaacuugg......    51     1      seq
..............................................ucagugcaugUcagaacuugg......    35     1      seq
..............................................ucaguCcaugacagaacuugg......    41     1      seq
..............................................ucCgugcaugacagaacuugg......    18     1      seq
..............................................ucagugcaCgacagaacuugg......    34     1      seq
..............................................ucagugcaugacagUacuugg......    8      1      seq
..............................................ucagugcaugaUagaacuugg......    71     1      seq
..............................................ucaAugcaugacagaacuugg......    46     1      seq
..............................................ucagugcaugUcagaacuugA......    1      2      seq
..............................................ucagugcCugacagaacuugg......    24     1      seq
..............................................ucagGgcauAacagaacuugg......    1      2      seq
..............................................ucagugcaugacagaacuuUg......    23     1      seq
..............................................ucagugcUugacagaacuugg......    5      1      seq
..............................................ucagugcaugacagaacuGgg......    6      1      seq
..............................................ucagugcaugacagaGcuugg......    24     1      seq
..............................................ucagugcaugacagaaUuugg......    43     1      seq
..............................................ucagugcaugacagaGcuugU......    1      2      seq
..............................................ucagugcaugGcagaacuugg......    28     1      seq
..............................................ucCgugcaugacagaacuugA......    1      2      seq
..............................................Ccagugcaugacagaacuugg......    46     1      seq
..............................................ucagugcaugacagGacuugg......    27     1      seq
..............................................ucagugGaugacagaacuugg......    10     1      seq
..............................................ucagugcauCacagaacCugg......    1      2      seq
..............................................ucagugcaugacagaaAuugg......    9      1      seq
..............................................ucagugcaugacaCaacuugg......    16     1      seq
..............................................ucagugcaugacagaaGuugg......    4      1      seq
..............................................ucagugcaAgacagaacuugg......    16     1      seq
..............................................ucUgugUaugacagaacuugg......    1      2      seq
..............................................ucagugcauAacagaacuugg......    39     1      seq
..............................................ucagugcaugacagaacuugU......    1511   1      seq
..............................................ucagugUaugacagaacuugU......    1      2      seq
..............................................ucagugcaugacagaacuugC......    42     1      seq
..............................................ucagugcaugCcCgaacuugg......    1      2      seq
..............................................ucagugcaugacaAaacuugg......    12     1      seq
..............................................ucagugcaugacagaacuuCg......    18     1      seq
..............................................ucagugcaugacagaCcuugg......    13     1      seq
..............................................ucaguAcaugacagaacuugg......    52     1      seq
..............................................ucaguUcaugacagaacuugg......    48     1      seq
..............................................ucagCgcaugacCgaacuugg......    1      2      seq
..............................................ucagugcauCacagaacuugg......    39     1      seq
..............................................ucagugcaugacagaacCugg......    60     1      seq
..............................................uUaUugcaugacagaacuugg......    1      2      seq
..............................................ucagugcauCacagaacuugA......    1      2      seq
..............................................ucagugcaugacagaacuuAU......    2      2      seq
..............................................ucagugcaugacagCacuugg......    36     1      seq
```

```
                                              mmu-miR-152-3p
       mmu-miR-152-5p
ccgggccuagguucugugauacacuccgacucgggcucuggagcagucagugcaugacagaacuugggcccgg

..............................................ucagugcaugacagaUcuugg......    3          1          seq
..............................................uUagugcaugacagaacuugg......    46         1          seq
..............................................ucagugcGugacagaacuugg......    43         1          seq
..............................................ucagCgcaugacagaacuugg......    134        1          seq
..............................................Gcagugcaugacagaacuugg......    11         1          seq
..............................................ucagugcaugacagaacuuAA......    12         2          seq
..............................................ucaCugcaugacagaacuugg......    33         1          seq
..............................................CcagugcaugacagaacuugU......    1          2          seq
..............................................ucaUugcaugacagaacuugg......    100        1          seq
..............................................ucaguUUaugacagaacuugg......    1          2          seq
..............................................ucUgugcaugacagaacuugg......    7          1          seq
..............................................ucagugcaugacUgaacuugg......    13         1          seq
..............................................ucGgugcaugacagaacuugU......    1          2          seq
..............................................ucagugcaugacagaacuuAg......    27         1          seq
..............................................ucagugcaugacagaacGugg......    5          1          seq
..............................................CcagugcauUacagaacuugg......    1          2          seq
..............................................ucagGUcaugacagaacuugg......    1          2          seq
..............................................ucagGgcaugacagaacuugg......    157        1          seq
..............................................ucagAgcaugacagaacuugg......    45         1          seq
..............................................ucagGgcaugacagaacuugU......    4          2          seq
..............................................ucaUugcaugaUagaacuugg......    1          2          seq
..............................................ucagugcaugacaCGacuugg......    1          2          seq
..............................................ucGgugcaugacagaacuugA......    1          2          seq
..............................................ucagAgcaugacagaacuugA......    1          2          seq
..............................................ucagugcaugCcagaacuugg......    52         1          seq
..............................................ucagugcaugacagaacAugg......    11         1          seq
..............................................ucagGgcaugacagaacuugA......    2          2          seq
..............................................ucUCugcaugacagaacuugg......    2          2          seq
..............................................uAagugcauAacagaacuugg......    1          2          seq
..............................................ucagugcaugacagaacuugg......    183272     0          seq
..............................................ucagugcauAacagaacuGgg......    1          2          seq
..............................................Acagugcaugacagaacuugg......    23         1          seq
..............................................ucagugcaGgacagaacuugg......    5          1          seq
..............................................ucagugcaugacagaacuCgg......    48         1          seq
..............................................ucagugcaugacagaacuCgU......    1          2          seq
..............................................ucagugcaugacCgaacuugg......    43         1          seq
..............................................ucagugcaugacaUaacuugg......    13         1          seq
..............................................ucagugcaugacagaacCugU......    1          2          seq
..............................................uGagugcaugacagaacuugg......    5          1          seq
..............................................ucagCgcaugacagaacuugA......    1          2          seq
..............................................ucagugcaugaGagaacuugg......    6          1          seq
..............................................ucaAugcaugacagaacuugA......    1          2          seq
..............................................ucagCUcaugacagaacuugg......    2          2          seq
..............................................ucagugAaugacagaacuugg......    17         1          seq
..............................................ucagugcaugacagaCcuuggC.....    1          2          seq
..............................................CcagugcaugacagaacuuggA.....    8          2          seq
..............................................ucagugcaugacagaacuGggA.....    1          2          seq
..............................................ucagugcaugacagaacuuggU.....    14144      1          seq
..............................................ucagugcaugCcagaacuuggg.....    2          1          seq
..............................................ucagugcaugGcagaacuuggU.....    5          2          seq
..............................................ucaguAcaugacagaacuuggU.....    4          2          seq
..............................................uUagugcaugacagaacuuggA.....    9          2          seq
..............................................ucagugcaugacagGacuuggU.....    2          2          seq
..............................................ucagugcaugacagaacuuggA.....    27121      1          seq
..............................................ucagugcaGgacagaacuuggA.....    1          2          seq
..............................................ucagugcaugacagaacuugUU.....    42         2          seq
..............................................ucaguAcaugacagaacuuggA.....    12         2          seq
..............................................ucagugcaugaGagaacuuggU.....    1          2          seq
..............................................ucagugcaugaUagaacuuggA.....    9          2          seq
..............................................ucagugcaugacagaacuugAA.....    30         2          seq
..............................................ucaguAcaugacagaacuuggg.....    1          1          seq
..............................................ucagugcaugacagaaGuuggA.....    1          2          seq
..............................................ucagugcGugacagaacuuggU.....    2          2          seq
..............................................uGagugcaugacagaacuuggA.....    3          2          seq
..............................................ucCgugcaugacagaacuuggA.....    5          2          seq
..............................................ucagugcaugacGgaacuuggg.....    5          1          seq
..............................................ucagugcaugacagaacuCggg.....    1          1          seq
..............................................ucagugcaugaUagaacuuggU.....    5          2          seq
..............................................ucUgugcaugacagaacuuggU.....    2          2          seq
```

```
                                                 mmu-miR-152-3p
      mmu-miR-152-5p
ccgggccuagguucugugauacacuccgacucgggcucuggagcagucagugcaugacagaacuugggcccgg

..............................................ucagugcaugaAagaacuuggA.....  2     2     seq
..............................................ucagugUaugacagaacuuggU.....  3     2     seq
..............................................ucagugcaugacagaaUuuggA.....  9     2     seq
..............................................ucagugcaCgacagaacuuggA.....  1     2     seq
..............................................ucaAugcaugacagaacuuggA.....  12    2     seq
..............................................ucagugcauAacagaacuuggU.....  2     2     seq
..............................................ucagugcaugacagaacuuggC.....  127   1     seq
..............................................ucaguCcaugacagaacuuggU.....  3     2     seq
..............................................ucaUugcaugacagaacuuggg.....  3     1     seq
..............................................ucagugcaCUacagaacuuggg.....  1     2     seq
..............................................ucagugcaugacagaacGuggA.....  2     2     seq
..............................................ucaAugcaugacagaacuuggg.....  2     1     seq
..............................................GcagugcaugacagaacuuggA.....  1     2     seq
..............................................ucagugcCugacagaacuuggA.....  2     2     seq
..............................................ucagugcaugacagaCcuuggU.....  1     2     seq
..............................................ucagugcaugacagaGcuuggU.....  2     2     seq
..............................................ucaCugcaugacagaacuuggg.....  2     1     seq
..............................................ucagugcGugacagaacuuggC.....  1     2     seq
..............................................ucagugcaugacagaGcuuggg.....  1     1     seq
..............................................ucagugGaugacagaacuuggg.....  1     1     seq
..............................................ucagugcaugacagaacuuggg.....  6955  0     seq
..............................................ucagugcCugacagaacuuggU.....  1     2     seq
..............................................ucagugcaugacagaaUuuggU.....  3     2     seq
..............................................ucaguUcaugacagaacuuggU.....  2     2     seq
..............................................ucagugcaugacagaCcuuggA.....  3     2     seq
..............................................ucagugGaugacagaacuuggA.....  1     2     seq
..............................................ucagugcUugacagaacuuggU.....  2     2     seq
..............................................ucagugcaugGcagaacuuggA.....  3     2     seq
..............................................ucagugcaugacCgaacuuggA.....  2     2     seq
..............................................ucagugcaugacagaacuuAgU.....  1     2     seq
..............................................ucagugcaugacaAaacuuggU.....  1     2     seq
..............................................ucagugcaugacagaacuugUg.....  5     1     seq
..............................................ucagugcaugacaAaacuuggA.....  1     2     seq
..............................................ucagugcaugacagaacuugAg.....  6     1     seq
..............................................ucagugcaugacagCacuuggU.....  1     2     seq
..............................................ucagugcaugacaUaacuuggU.....  2     2     seq
..............................................ucagugcaugacagaacuugCg.....  1     1     seq
..............................................ucagGgcaugacaUaacuuggg.....  1     2     seq
..............................................ucagugcaugacagaacCuggU.....  6     2     seq
..............................................ucagugcaugacagaUcuuggU.....  2     2     seq
..............................................ucagugcauUacagaacuuggA.....  10    2     seq
..............................................ucaguCcaugacagaacuuggA.....  10    2     seq
..............................................ucagugcaugCcagaacuuggA.....  6     2     seq
..............................................ucagugcaugacagaacuuAgA.....  4     2     seq
..............................................ucGgugcaugacagaacuuggA.....  5     2     seq
..............................................ucCgugcaugacagaacuuggU.....  3     2     seq
..............................................ucagAgcaugacagaacuuggg.....  3     1     seq
..............................................ucagugcaugacUgaacuuggg.....  1     1     seq
..............................................ucaAugcaugacagaacuuggU.....  1     2     seq
..............................................ucagugcaugacagCacuuggA.....  3     2     seq
..............................................ucagugcaugacagaacuugAU.....  9     2     seq
..............................................ucGgugcaugacagaacuuggU.....  3     2     seq
..............................................ucagugcaugacagGacuuggA.....  5     2     seq
..............................................ucagugcaugacagGacuuggg.....  1     1     seq
..............................................ucagugcaCgacagaacuuggg.....  1     1     seq
..............................................ucagugcaugUcagaacuuggA.....  4     2     seq
..............................................ucagGgcaugacagaacuuggA.....  37    2     seq
..............................................ucagugcaugacagCacuuggg.....  1     1     seq
..............................................ucagugcGugacagaacuuggg.....  5     1     seq
..............................................ucagugcaugacagaacuuAgg.....  1     1     seq
..............................................ucagugcaugacagaacuugAC.....  1     2     seq
..............................................ucagugcaugaAagaacuuggU.....  2     2     seq
..............................................ucagugcaugacaUaacuuggg.....  1     1     seq
..............................................ucagugcaugacagaGcuuggA.....  3     2     seq
..............................................ucagugcaugacCgaacuuggg.....  4     1     seq
..............................................ucaCugcaugacagaacuuggU.....  3     2     seq
..............................................ucagugcaugacaUaacuuggA.....  4     2     seq
..............................................ucagugcaugacagaacuuCgg.....  2     1     seq
..............................................ucagugcaugacUgaacuuggA.....  2     2     seq
```

```
                                              mmu-miR-152-3p
       mmu-miR-152-5p
ccgggccuagguucugugauacacuccgacucgggcucuggagcagucagugcaugacagaacuugggcccgg

..............................................ucagugcaAgacagaacuuggA.....    2          2          seq
..............................................ucagugcaugacagaUcuuggg.....    1          1          seq
..............................................ucagugUaugacagaacuuggA.....    3          2          seq
..............................................ucaguUcaugacagaacuuggA.....    8          2          seq
..............................................ucagCgcaugacagaacuuggA.....    26         2          seq
..............................................ucagCgcaugacagaacuuggg.....    7          1          seq
..............................................ucGgugcaugacagaacuuggg.....    1          1          seq
..............................................ucagugcUugacagaacuuggA.....    1          2          seq
..............................................ucagAgcaugacagaacuuggA.....    10         2          seq
..............................................ucagugcauCacagaacuuggA.....    5          2          seq
..............................................ucagugcauAacagaacuuggg.....    2          1          seq
..............................................ucagugcGugacagaacuuggA.....    6          2          seq
..............................................ucagugcaugacagaaGuuggg.....    1          1          seq
..............................................ucagugcaugacagaacuCggU.....    7          2          seq
..............................................ucagugcauCacagaacuuggg.....    3          1          seq
..............................................ucagugcaugacGgaacuuggA.....    4          2          seq
..............................................ucagugcaugacagaUcuuggA.....    3          2          seq
..............................................ucagugcauAacagaacuuggA.....    11         2          seq
..............................................ucagugcaCgacagaacuuggU.....    1          2          seq
..............................................ucagugcaugaUagaacuuggg.....    3          1          seq
..............................................ucagugcaugacagaacuAggA.....    1          2          seq
..............................................ucagugcaugaAagaacuuggg.....    2          1          seq
..............................................ucagugcaugacagaacuugUA.....    37         2          seq
..............................................ucagugcaugacaCaacuuggA.....    3          2          seq
..............................................Ccagugcaugacagaacuuggg.....    2          1          seq
..............................................ucagugcaugacagaacCuggg.....    2          1          seq
..............................................ucagugcaugacaCaacuuggU.....    2          2          seq
..............................................GcagugcaugacagaacuuggU.....    1          2          seq
..............................................ucagugcaugacagaacuGggU.....    2          2          seq
..............................................ucagugUaugacagaacuuggg.....    2          1          seq
..............................................ucagGgcaugacagaacuuggg.....    11         1          seq
..............................................ucagugcaugacagUacuuggU.....    1          2          seq
..............................................ucagugcaugacagaaUuuggg.....    1          1          seq
..............................................ucagAgcaugacagaacuuggU.....    3          2          seq
..............................................ucagugcaugacagaacuuCgU.....    6          2          seq
..............................................ucagugcaugacagaacuugCA.....    4          2          seq
..............................................ucagugcaugacGgaacuuggU.....    4          2          seq
..............................................ucaUugcaugacagaacuuggA.....    10         2          seq
..............................................ucagugcaugacagaacuuUgA.....    9          2          seq
..............................................ucagugcaugacagaacuCggA.....    4          2          seq
..............................................ucagugcaugacagaacuuUgg.....    4          1          seq
..............................................AcagugcaugacagaacuuggA.....    6          2          seq
..............................................ucagugcaugaGagaacuuggg.....    1          1          seq
..............................................ucagCgcaugacagaacuuggU.....    20         2          seq
..............................................ucagugcaugacagaacuuCgA.....    4          2          seq
..............................................uGagugcaugacagaacuuggU.....    1          2          seq
..............................................ucagugcaugacagaacCuggA.....    16         2          seq
..............................................ucagugcaugCcagaacuuggU.....    4          2          seq
..............................................ucagGgcaugacagaaGuuggg.....    1          2          seq
..............................................ucCgugcaugacagaacuuggg.....    4          1          seq
..............................................ucagGgcaugacagaacuuggU.....    14         2          seq
..............................................CcagugcaugacagaacuuggU.....    4          2          seq
..............................................ucagugcaugacCgaacuuggU.....    3          2          seq
..............................................ucaguUcaugacagaacuuggg.....    3          1          seq
..............................................ucagugcauUacagaacuuggU.....    14         2          seq
..............................................ucaCugcaugacagaacuuggA.....    13         2          seq
..............................................ucagugcaugacagaacAuggA.....    2          2          seq
..............................................ucaUugcaugacagaacuuggU.....    4          2          seq
..............................................ucagugcaugacagaacuuggAA....    1455       2          seq
..............................................ucagugcaugacagaacuuggUc....    14         1          seq
..............................................ucagugcaAgacagaacuugggA....    1          2          seq
..............................................ucagugcaugacagaacuCgggU....    1          2          seq
..............................................ucGgugcaugacagaacuugggA....    1          2          seq
..............................................ucagugcaugacGgaacuugggA....    2          2          seq
..............................................ucagugcaugacaUaacuugggU....    1          2          seq
..............................................ucagugcaugacagaacuuggCU....    11         2          seq
..............................................ucaguAcaugacagaacuugggA....    1          2          seq
..............................................ucagugcCugacagaacuugggU....    1          2          seq
..............................................ucagugcaugacagaacuuggCA....    7          2          seq
```

```
                                              mmu-miR-152-3p
       mmu-miR-152-5p
ccgggccuagguucugugauacacuccgacucgggcucuggagcagucagugcaugacagaacuugggcccgg

..............................................ucagugcaugacagaacuuggUA....    1339    2    seq
..............................................ucaguUcaugacagaacuuggAc....    1       2    seq
..............................................ucagugcaugacagaacuugggU....    866     1    seq
..............................................ucagugUaugacagaacuugggA....    1       2    seq
..............................................ucagugcaugacCgaacuugggU....    1       2    seq
..............................................ucagugcaugacagaacuugggc....    8       0    seq
..............................................ucaUugcaugacagaacuugggA....    1       2    seq
..............................................ucagugcaugacagaacuuggAG....    267     2    seq
..............................................ucagugcaugacagaacCugggU....    2       2    seq
..............................................ucagCgcaugacagaacuugggA....    2       2    seq
..............................................ucagugcaugacagaacuugggG....    9       1    seq
..............................................ucagugcaugacagaacuuggUG....    30      2    seq
..............................................GcagugcaugacagaacuugggA....    1       2    seq
..............................................ucagugcaugacGgaacuugggU....    1       2    seq
..............................................ucaCugcaugacagaacuugggA....    1       2    seq
..............................................ucagugcaugacagaacuuggAU....    584     2    seq
..............................................ucagGgcaugacagaacuugggA....    2       2    seq
..............................................ucagugcaugacagaacuuUggA....    1       2    seq
..............................................ucagugcaCgacagaacuugggA....    2       2    seq
..............................................ucagugcaugacaUaacuugggA....    1       2    seq
..............................................ucagugcaugacagaacuugAgU....    1       2    seq
..............................................ucagugcaugacagaacuugAgA....    4       2    seq
..............................................ucagugcaugacagaacuugAAc....    1       2    seq
..............................................ucagugcaugacagaacuuggAc....    66      1    seq
..............................................ucagugcaugacagaacuuAggU....    1       2    seq
..............................................ucagugcaugaUagaacuugggU....    2       2    seq
..............................................ucagugcaugacagaacuugggA....    1866    1    seq
..............................................ucagugcaugaUagaacuugggA....    1       2    seq
..............................................ucagugAaugacagaacuugggU....    2       2    seq
..............................................ucagugcaugacagaacuuggCG....    1       2    seq
..............................................ucagugcaAgacagaacuugggG....    1       2    seq
..............................................ucCgugcaugacagaacuugggA....    1       2    seq
..............................................ucagAgcaugacagaacuugggA....    1       2    seq
..............................................ucagugcaugacagaacuuggUU....    1295    2    seq
..............................................ucagugcaugacagaacuuAggA....    1       2    seq
..............................................ucagugcaugacagaacuugUgA....    4       2    seq
..............................................ucagAgcaugacagaacuugggU....    1       2    seq
..............................................ucagugcaugacagaacuugggAA...    162     2    seq
..............................................ucagugcaugacagaacuuggUUc...    11      2    seq
..............................................ucagugcaugacagaacuuggAAc...    105     2    seq
..............................................ucagugcaugacagaacuugggAG...    60      2    seq
..............................................ucagugcaugacagaacuugggUG...    2       2    seq
..............................................ucagugcaugacagaacuugggUU...    119     2    seq
..............................................ucagugcaugacagaacuuggUcU...    6       2    seq
..............................................ucagugcaugacagaacuugggAU...    182     2    seq
..............................................ucagugcaugacagaacuuggUAc...    34      2    seq
..............................................ucaUugcaugacagaacuugggAc...    1       2    seq
..............................................ucagugcaugacagaacuugggUA...    70      2    seq
..............................................ucagugcaugacagaacuuggUcc...    4       1    seq
..............................................ucagugcaugacagaacuuggAcG...    2       2    seq
..............................................ucagugcaugacagaacuuggAcA...    8       2    seq
..............................................ucagugcaugacagaacuuggAcU...    7       2    seq
..............................................ucagugcaugacagaacuuggUcA...    1       2    seq
..............................................ucagugcaugacagaacuuggAUc...    3       2    seq
..............................................ucagugcaugacagaacuuggUcG...    1       2    seq
..............................................ucagugcaugacagaacuugggGA...    2       2    seq
..............................................ucagugcaugacagaacuugggAc...    13      1    seq
..............................................ucagugcaugacagaacuugggAAc..    9       2    seq
..............................................ucagugcaugacagaacuugggAcU..    2       2    seq
..............................................ucagugcaugacagaacuuggAcGc..    2       2    seq
..............................................ucagugcaugacagaacuugggAcA..    7       2    seq
...............................................cagugcaugacagaacu.........    1       0    seq
...............................................cagugcauAacagaacu.........    1       1    seq
...............................................cagugcaugacagaacuu........    4       0    seq
...............................................cagugcaugacagaacuGg.......    1       1    seq
...............................................cagugcaugacagaacuug.......    8       0    seq
...............................................cagugcaugacagaacuuU.......    1       1    seq
...............................................cagGgcauUacagaacuugg......    1       2    seq
...............................................Uagugcaugacagaacuugg......    1       1    seq
```

```
                                              mmu-miR-152-3p
       mmu-miR-152-5p
ccgggccuagguucugugauacacuccgacucgggcucuggagcagucagugcaugacagaacuugggcccgg

...............................................cagugcaugacUgaacuugg......   1      1      seq
...............................................cagugcaugacagaGcuugg......   1      1      seq
...............................................cagGgcaugacagaacuugg......   59     1      seq
...............................................cagugcaugacaAaacuugg......   1      1      seq
...............................................cagugcaugacGgaacuugg......   3      1      seq
...............................................cagugcaugacagaCcuugg......   1      1      seq
...............................................cagugcaugGcagaacuugg......   2      1      seq
...............................................cagugcaugaAagaacuugg......   2      1      seq
...............................................cagugcaugacaCaacuugg......   1      1      seq
...............................................cagugcaugacagaacuAgg......   1      1      seq
...............................................cagugcaugacagaacuugC......   2      1      seq
...............................................cagCgcaugacagaacuugU......   1      2      seq
...............................................cagugcaugacagaacuugg......   3317   0      seq
...............................................cCgugcaugacagaacuugg......   2      1      seq
...............................................cagugcaugacagaaUuugg......   2      1      seq
...............................................cGgugcaugacagaacuugg......   1      1      seq
...............................................cagugcaugacagaacuGgg......   1      1      seq
...............................................UCgugcaugacagaacuugg......   1      2      seq
...............................................cagugcaugCcagaacuugg......   27     1      seq
...............................................cagugcaugacagaacuuUg......   1      1      seq
...............................................GagugcaugacagaacuugA......   1      2      seq
...............................................cagGgcaugacagaacuugU......   1      2      seq
...............................................cagugcaugacagaacuugA......   12     1      seq
...............................................cagugcaugUcagaacuugg......   1      1      seq
...............................................Gagugcaugacagaacuugg......   2      1      seq
...............................................Aagugcaugacagaacuugg......   13     1      seq
...............................................cUgugcaugacagaacuugg......   1      1      seq
...............................................cagugcCugacagaacuugg......   1      1      seq
...............................................caguAcaugacagaacuugg......   2      1      seq
...............................................cagugcauAacagaacuugg......   2      1      seq
...............................................caUugcaugacagaacuugg......   2      1      seq
...............................................cagugcaugacagaacuugU......   29     1      seq
...............................................caguCcaugacagaacuugg......   2      1      seq
...............................................cagGgcaugacagaacuuggg.....   4      1      seq
...............................................cagugcauUacagaacuuggU.....   1      2      seq
...............................................cagugcaugacagaacuugUA.....   4      2      seq
...............................................caAugcaugacagaacuuggg.....   2      1      seq
...............................................UagugcaugacagaacuuggA.....   1      2      seq
...............................................cagGgcaugacagaacuuggU.....   4      2      seq
...............................................AagugcaugacagaacuuggA.....   1      2      seq
...............................................cagugcaugacagaacuugUU.....   1      2      seq
...............................................cagugcaugacagaacuuggA.....   614    1      seq
...............................................cagGgcaugacagaacuuggA.....   12     2      seq
...............................................cagugcaugacagaacuuUgA.....   1      2      seq
...............................................cagugcUugacagaacuuggU.....   1      2      seq
...............................................cagugcaugCcagaacuuggU.....   5      2      seq
...............................................cagugcaugacagaUcuuggA.....   1      2      seq
...............................................cagugcauUacagaacuuggA.....   1      2      seq
...............................................cagugcaugacagaacCuggU.....   1      2      seq
...............................................AagugcaugacagaacuuggU.....   2      2      seq
...............................................Uagugcaugacagaacuuggg.....   1      1      seq
...............................................cGgugcaugacagaacuuggU.....   1      2      seq
...............................................cagugcaugacagaacuuggU.....   343    1      seq
...............................................cagugcaugCcagaacuuggA.....   2      2      seq
...............................................UagugcaugacagaacuuggU.....   2      2      seq
...............................................cagugcaugacagaCcuuggA.....   1      2      seq
...............................................cagugcaugaAagaacuuggA.....   1      2      seq
...............................................cagugcaugacagaacuCggg.....   1      1      seq
...............................................cagugcaugacagaacuuggC.....   4      1      seq
...............................................cagugcaugacagaacuuggg.....   127    0      seq
...............................................cagugcaAgacagaacuuggA.....   1      2      seq
...............................................caUuUcaugacagaacuuggg.....   1      2      seq
...............................................cagugcaugacagaacuuggAG....   3      2      seq
...............................................cagugcaugacagaacuuggUc....   1      1      seq
...............................................AagugcaugacagaacuugggA....   1      2      seq
...............................................cagGgcaugacagaacuugggU....   1      2      seq
...............................................cagGgcaugacagaacuugggA....   1      2      seq
...............................................cagugcaugacagaacuugggU....   19     1      seq
...............................................cagugcaugacagaacuuggAA....   24     2      seq
```

```
                                              mmu-miR-152-3p
       mmu-miR-152-5p
ccgggccuagguucugugauacacuccgacucgggcucuggagcagucagugcaugacagaacuugggcccgg

...............................................cagugcaugacagaacuuggAc....    3      1     seq
...............................................cagugcaugacagaacuuggUA....    30     2     seq
...............................................cagugcaugacagaacuuggAU....    7      2     seq
...............................................cagugcaugacagaacuuggUU....    32     2     seq
...............................................cagugcaugacagaacuugggA....    41     1     seq
...............................................cagugcaugacagaacuuggUAc...    2      2     seq
...............................................cagugcaugacagaacuuggAGc...    1      2     seq
...............................................cagugcaugacagaacuugggUA...    4      2     seq
...............................................cagugcaugacagaacuugggAU...    4      2     seq
...............................................cagugcaugacagaacuugggUU...    7      2     seq
...............................................cagugcaugacagaacuuggAAc...    2      2     seq
...............................................cagugcaugacagaacuugggAG...    2      2     seq
...............................................cagugcaugacagaacuugggAA...    4      2     seq
...............................................cagugcaugacagaacuugggcAA..    1      2     seq
................................................agugcaugacagaacuu........    3      0     seq
................................................agugcaugacagaacuug.......    4      0     seq
................................................aCugcaugacagaacuugg......    1      1     seq
................................................agugcaugacagaacuugC......    1      1     seq
................................................agGgcaugacagaacuugg......    3      1     seq
................................................agugcaugacagCacuugg......    3      1     seq
................................................aAugcaugacagaacuugg......    2      1     seq
................................................agugcauUacagaacuugg......    1      1     seq
................................................agugcaugCcagaacuugg......    1      1     seq
................................................agugUaugacagaacuugg......    1      1     seq
................................................agugcaugacGgaacuugg......    1      1     seq
................................................agugcaugacagaacuugU......    22     1     seq
................................................agugcaugacagaacuuAg......    1      1     seq
................................................UCugcaugacagaacuugg......    10     2     seq
................................................agugcaugacagGacuugg......    1      1     seq
................................................agAgcaugacagaacuugg......    1      1     seq
................................................agugcaCgacagaacuugg......    1      1     seq
................................................agugcaugUcagaacuugg......    2      1     seq
................................................agugcaugaUagaacuugg......    2      1     seq
................................................agugcaugacagaacuugg......    2572   0     seq
................................................aguUcaugacagaacuugg......    1      1     seq
................................................agugcGugacagaacuugg......    3      1     seq
................................................agugcaugacagaacuuAA......    1      2     seq
................................................agugcaugGcagaacuugg......    2      1     seq
................................................Cgugcaugacagaacuugg......    1      1     seq
................................................agugcaugacagaacuugA......    9      1     seq
................................................aCugcaugacagaacuuggA.....    1      2     seq
................................................agugcauUacagaacuuggg.....    1      1     seq
................................................agugcaugacagCacuuggA.....    1      2     seq
................................................aguAcaugacagaacuuggU.....    1      2     seq
................................................agugcaugacagaacuuggU.....    274    1     seq
................................................agugcaugacagaacuuggg.....    98     0     seq
................................................agGgcaugacagaacuuggA.....    1      2     seq
................................................agugcaugacagaacuuggC.....    3      1     seq
................................................agugcaugacagaacuuggA.....    473    1     seq
................................................agugcaugCcagaacuuggU.....    2      2     seq
................................................agugcaugacagaacuuggAU....    9      2     seq
................................................agugcaugacagaacuugggA....    30     1     seq
................................................agugcaugacagaacuuggAG....    4      2     seq
................................................agugcaugacagaacuuggUA....    21     2     seq
................................................agugcaugacagaacuuggAA....    16     2     seq
................................................agugcaugacagaacuuggAc....    1      1     seq
................................................agugcaugacagaacuugggU....    16     1     seq
................................................agugcaugacagaacuuggUU....    14     2     seq
................................................agugcaugacagaacuugggAU...    4      2     seq
................................................agugcaugacagaacuuggAAc...    1      2     seq
................................................agugcaugacagaacuuggUAc...    1      2     seq
................................................agugcaugacagaacuugggAA...    1      2     seq
................................................agugcaugacagaacuugggUU...    7      2     seq
................................................agugcaugacagaacuugggUA...    1      2     seq
.................................................gugcaugacagaacuugg......    70     0     seq
.................................................gugcaugacagaacuugU......    1      1     seq
.................................................gugcaugacagaacuuggA.....    8      1     seq
.................................................gugcaugacagaacuuggU.....    6      1     seq
.................................................gugcaugacagaacuuggAG....    1      2     seq
```

```
                                         mmu-miR-152-3p
       mmu-miR-152-5p
ccgggccuagguucugugauacacuccgacucgggcucuggagcagucagugcaugacagaacuugggcccgg

.................................................gugcaugacagaacuuggAA....    2    2    seq
.................................................gugcaugacagaacuuggUU....    3    2    seq
.................................................gugcaugacagaacuuggUcA...    1    2    seq
..................................................uUcaugacagaacuugg......    1    1    seq
..................................................ugcaugacagaacuugg......    27   0    seq
..................................................ugcaugacagaacuugU......    1    1    seq
..................................................ugcaugacagaacuuggg.....    2    0    seq
..................................................ugcaugacagaacuuggA.....    3    1    seq
..................................................ugcaugacagaacuuggU.....    2    1    seq
..................................................ugcaugacagaacuuggUU....    1    2    seq
..................................................ugcaugacagaacuuggAA....    1    2    seq
...................................................gcaugacagaacuuggU.....    4    1    seq
...................................................gcauUacagaacuuggA.....    1    2    seq
...................................................gcaugacagaacuuggA.....    8    1    seq
...................................................gcaugacagaacuugAA.....    1    2    seq
...................................................gcaugaUagaacuuggU.....    1    2    seq
...................................................gcaugacagaacuuggUA....    1    2    seq
...................................................gcaugacagaacuugggU....    1    1    seq
...................................................gcaugacagaacuuggAA....    1    2    seq
...................................................gcaugacagaacuugggA....    4    1    seq
...................................................gcaugacagaacuugggUA...    1    2    seq
```
