# Supplementary material for: mirPRo–a novel standalone program for differential expression and variation analysis of miRNAs
Source: Sci Rep. 2015 Oct 5;5:14617. doi: 10.1038/srep14617 (PMC4592965; doi:10.1038/srep14617)
Supplement: Supplementary Data 12-21 [file srep14617-s25.zip › Supplementary Data 15.pdf]

miRBase precursor : mmu-mir-152
Total read count : 251779
mmu-miR-152-5p read count : 631
mmu-miR-152-3p read count : 251133
remaining reads : 15

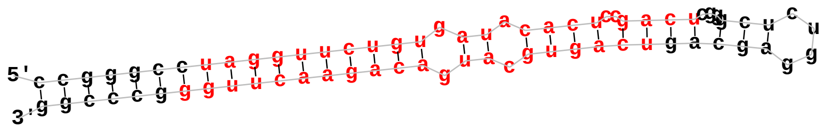

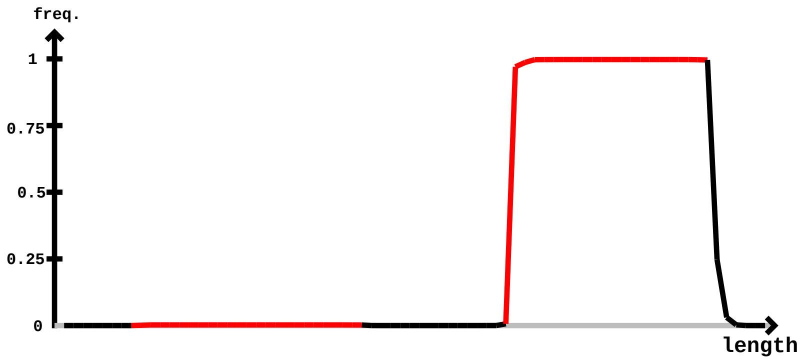


```
                                                          mmu-miR-152-3p
        mmu-miR-152-5p
5'- ccgggccuagguucugugauacacuccgacucgggcucuggagcagucagugcaugacagaacuugggcccgg   -3'    exp
    (((((((((((((((.((.((((..((((...((((....)))))))))))).)).)))))))))))))))   reads    mm    sample
    .......uagguucugugauacacu................................................   1        0     seq
    .......uagguucugugauacacuA...............................................   1        1     seq
    .......uagguucugugauacacucc..............................................   2        0     seq
    .......uagguucugugauacacuccg.............................................   4        0     seq
    .......uagguucugugauacacuccgU............................................   4        1     seq
    .......uagguucugugauGcacuccga............................................   1        1     seq
    .......uagguucugugauacacuccga............................................   26       0     seq
    .......uagguucugugauacacucUga............................................   1        1     seq
    .......uagguucugugauacacucUgG............................................   1        2     seq
    .......uagguucugugCuacacuccgU............................................   1        2     seq
    .......uagguGcugugauacacuccga............................................   1        1     seq
    .......uagguucugugauacacuccgaG...........................................   1        1     seq
    .......uGgguucugugauacacuccgac...........................................   1        1     seq
    .......uagguucugugauacacuccgac...........................................   12       0     seq
    .......uagguucugugauacacuccgaA...........................................   1        1     seq
    .......uagguucugugauacacuccgaU...........................................   4        1     seq
    .......uagguGcugugauacacuccgacu..........................................   3        1     seq
    .......uagguucugugauacacuccgacG..........................................   1        1     seq
    .......uagguucugugauacacuccgaAG..........................................   1        2     seq
    .......uagguucugugGuacCcuccgacu..........................................   1        2     seq
    .......uagguCcCgugauacacuccgacu..........................................   1        2     seq
    .......uaAguucugugauacacuccgacu..........................................   1        1     seq
    .......uagguucugugauUcacuccgacu..........................................   2        1     seq
    .......uagguucugugaCacacuccgacu..........................................   1        1     seq
    .......uagguucugugauacacuccgaAA..........................................   1        2     seq
    .......uagguucugugauacacuccgacA..........................................   1        1     seq
    .......uagguAcugugauacacuccgacu..........................................   1        1     seq
    .......uagguucugugauacacuccgacC..........................................   1        1     seq
    .......uGgguucugugauacacuccgacu..........................................   1        1     seq
    .......uagguucugugauacacucGgacu..........................................   3        1     seq
    .......uagguucugugauacacuccgacu..........................................   192      0     seq
    .......uagguucugugauacCcuccgacu..........................................   1        1     seq
    .......uagUucugugauacacuccgacu...........................................   1        1     seq
    .......uagguucugugauacacuccgacuU.........................................   1        1     seq
```

mmu-miR-152-3p

mmu-miR-152-5p

ccgggccuagguucugugauacacuccgacucgggcucuggagcagucagugcaugacagaacuugggcccgg

........agguucugugauacacu..	1	0	seq
........agguucugugauacacuc...	1	0	seq
........agguucugugauacacuccgU..	1	1	seq
........agguucugugauacacuccga..	4	0	seq
........agguucugugauacacuccgaU...	1	1	seq
........agguuGugugauacacuccgac...	1	1	seq
........agguucugugauacacuccgac...	8	0	seq
........agguucugugauacacCccgacu..	1	1	seq
........agguucugugauacacuccgacu..	275	0	seq
........aggGucugugauacacuccgacA..	1	2	seq
........agguuAugugauacacuccgacu..	2	1	seq
........aggGucugugaCacacuccgacu..	1	2	seq
........aUguucugugauacacuccgacu..	1	1	seq
........aggGucugugauacacuccgacu..	3	1	seq
........agguucugugauacacuccgaAu..	1	1	seq
........aggCucugugauacacuccgacu..	1	1	seq
........agguucugugauacaUuccgacu..	1	1	seq
........agguuGugugauacacuccgacu..	1	1	seq
........agUuucugugauacacuccgacu..	1	1	seq
........agguucugugauacacuccgacA..	1	1	seq
........agguucugugauaUacuccgacu..	3	1	seq
........agguGcugugauacacuccgacu..	1	1	seq
........agguuUugugauacacuccgacu..	4	1	seq
........agguucugugauaGacuccgacu..	1	1	seq
........agguucugugaGacacuccgacu..	1	1	seq
........aggGucugugaAacacuccgacuc...	1	2	seq
........agguucugugauacacuccgacuc...	14	0	seq
........aggGucugugauacacuccgacuc...	2	1	seq
........aggAucugugauacacuccgacuc...	1	1	seq
........aggCucugugauacacuccgacuc...	1	1	seq
........agguucugugauaUacuccgacuc...	1	1	seq
........agguucugugauacacuccgCcucU..	1	2	seq
........agguucugugauacacuccgacuGU..	1	2	seq
........agguucugugauacacuccgacucU..	5	1	seq
.........gguucugugauacacuccgacu..	4	0	seq
.........gguGcugugauacacuccgacu..	1	1	seq
..........UuucugugauacacuccAacu..	1	2	seq
...........uucugugauacacuccga..	1	0	seq
...........uuGugugauacacuccgacu..	1	1	seq
...........uucugugauacacuccgacu..	1	0	seq
...........uucugugauacacuccgacuc...	1	0	seq
............ucugugauacacuccgacu..	1	0	seq
....cGUucagugcaugacagaacuugg......	5	2	seq
....cUgucCgugcaugacagaacuugg......	1	2	seq
....ACgucagugcaugacagaacuugg......	1	2	seq
....cUguGagugcaugacagaacuugg......	1	2	seq
....cagucagugcaugacagaacuugg......	2	0	seq
....GCgucagugcaugacagaacuugg......	1	2	seq
....caCucagugcaugacagaacuugg......	1	1	seq
....cUCucagugcaugacagaacuugg......	2	2	seq
....caUucagugcaugacagaacuugg......	1	1	seq
....agucagugcaugacagaacuugg......	2	0	seq
....CCucagugcaugacagaacuugg......	1	2	seq
....UCucagugcaugacagaacuugg......	1	2	seq
....aCucagugcaugacagaacuugg......	1	1	seq
....Cgucagugcaugacagaacuugg......	1	1	seq
....gCcagugcaugacagaacuug.......	1	1	seq
....gucagugcaugacagaacuug.......	1	0	seq
....guGagugcaugacagaacuugg......	1	1	seq
....gucCgugcaugacagCacuugg......	2	2	seq
....gucagugcaugacagGacuugg......	1	1	seq
....Cucagugcaugacagaacuugg......	26	1	seq
....gCcagugcaugacagaacuugg......	5	1	seq
....gCcCgugcaugacagaacuugg......	1	2	seq
....gucagugcaugacaUaacuugg......	1	1	seq
....gucUgugcaugacagaacuugg......	1	1	seq
....gucGgugcaugacagaacuugg......	3	1	seq
....gucagugcaugacagaacuCgg......	1	1	seq
....Uucagugcaugacagaacuugg......	2	1	seq

```
                                              mmu-miR-152-3p
       mmu-miR-152-5p
ccgggccuagguucugugauacacuccgacucgggcucuggagcagucagugcaugacagaacuugggcccgg

.............................................guGCgugcaugacagaacuugg......   1      2      seq
.............................................gAcagugcaugacagaacuugg......   11     1      seq
.............................................gucagAgcaugacagaacuugg......   3      1      seq
.............................................CGcagugcaugacagaacuugg......   1      2      seq
.............................................gucaguAcaugacagaacuugg......   1      1      seq
.............................................gucagugcaugacagCacuugg......   3      1      seq
.............................................gucagugcaugCcagCacuugg......   1      2      seq
.............................................gucCgugcaugacagaacuugg......   8      1      seq
.............................................gucCgugcaugCcagaacuugg......   1      2      seq
.............................................gucagugcaugacagaacuugU......   3      1      seq
.............................................gucagugcaugGcagCacuugg......   1      2      seq
.............................................gucagugcaugacagaGcuugg......   1      1      seq
.............................................CucGgugcaugacagaacuugg......   1      2      seq
.............................................gucagugcaugacagaacuugC......   2      1      seq
.............................................gucagugcaugacGgaacuugg......   6      1      seq
.............................................gucagugcaugGcagaacuugg......   1      1      seq
.............................................gucagGgcaugacagaacuugg......   8      1      seq
.............................................gucagCgcaugacagaacuugg......   1      1      seq
.............................................gGcagugcaugacagaacuugg......   19     1      seq
.............................................gucagugcaugacUgaacuugg......   3      1      seq
.............................................gucagugcaugacagUacuugg......   5      1      seq
.............................................gucagugcaugacagaacuuAg......   1      1      seq
.............................................gucagugcaugacagaacuugg......   1178   0      seq
.............................................guAagugcaugacagaacuugg......   8      1      seq
.............................................gucagugcaugUcagaacuugg......   1      1      seq
.............................................gucagugcaugaUagaacuugg......   3      1      seq
.............................................gAcagugcaugacagaacuugU......   1      2      seq
.............................................gucagugcaugCcagaacuugg......   1      1      seq
.............................................gucagugcaugacagaacuGgg......   10     1      seq
.............................................gucagugcaugacUgaacuuggA.....   1      2      seq
.............................................CucagugcaugacagaacuuggA.....   3      2      seq
.............................................gGcagugcaugacagaacuuggU.....   3      2      seq
.............................................gucagugcaugacUgaacuuggU.....   2      2      seq
.............................................gucagAgcaugacagaacuuggU.....   1      2      seq
.............................................gucagugcaugacagaacuuggU.....   75     1      seq
.............................................guGagugcaugacagaacuuggU.....   1      2      seq
.............................................gucagCgcaugacagaacuuggA.....   1      2      seq
.............................................gucagugcaugacagaacuAggU.....   2      2      seq
.............................................gucagugcaugacagaacuuggg.....   1      0      seq
.............................................Cucagugcaugacagaacuuggg.....   1      1      seq
.............................................gucagGgcaugacagaacuuggA.....   3      2      seq
.............................................gucagugcGugacagaacuuggU.....   1      2      seq
.............................................gucagugcaugacagaacuGggA.....   3      2      seq
.............................................gucaUugcaugacagaacuuggU.....   2      2      seq
.............................................gucagugcaugacagaacuuggC.....   1      1      seq
.............................................gCcagugcaugacagaacuuggA.....   1      2      seq
.............................................AucagugcaugacagaacuuggA.....   1      2      seq
.............................................gucagugcaugacagaacuuggA.....   166    1      seq
.............................................gucagugcaugacagaacuCggA.....   1      2      seq
.............................................CucagugcaugacagaacuuggU.....   6      2      seq
.............................................gucagugcaugacagaacuuggAG....   1      2      seq
.............................................gucagugcaugacagaacuuggUA....   3      2      seq
.............................................CucagugcaugacagaacuugggA....   3      2      seq
.............................................gucagugcaugacagaacuuggAA....   1      2      seq
.............................................gucagugcaugacagaacuuggAcc...   2      1      seq
..............................................uGagugcaugacagaac..........   1      1      seq
..............................................ucagugcaugacagaac..........   7      0      seq
..............................................ucagugcaugacagaacu.........   17     0      seq
..............................................ucagugcaugacagaacuu........   111    0      seq
..............................................ucCgugcaugacagaacuu........   1      1      seq
..............................................ucaguCcaugacagaacuu........   1      1      seq
..............................................ucagugcaugacCCaacuu........   1      2      seq
..............................................ucagugcaugaGagaCcuu........   1      2      seq
..............................................ucaguUcaugacagaacuu........   1      1      seq
..............................................ucaCugcaugacagaacuu........   1      1      seq
..............................................uUagugcaugacagaacuug.......   1      1      seq
..............................................ucagugcaugacagaacuuU.......   4      1      seq
..............................................ucagugcaugacagaCcuug.......   2      1      seq
..............................................ucagugcaugacagaacuAA.......   1      2      seq
```

```
                                              mmu-miR-152-3p
       mmu-miR-152-5p
ccgggccuagguucugugauacacuccgacucgggcucuggagcagucagugcaugacagaacuugggcccgg

..............................................ucagugcaugacCgaacuug.......    1          1          seq
..............................................ucGgugcaugacagaacuug.......    1          1          seq
..............................................ucCgugcaugacagaGcuug.......    1          2          seq
..............................................ucCgugcaugacagaacuug.......    2          1          seq
..............................................uGagugcaugacagaacuug.......    4          1          seq
..............................................ucagugcaugacagaacuGg.......    5          1          seq
..............................................ucagCgcaugacagaacuug.......    1          1          seq
..............................................ucagugcaugacagaacuug.......    215        0          seq
..............................................ucagAgcaugacagaacuug.......    1          1          seq
..............................................ucagugcaugaAagaCcuug.......    1          2          seq
..............................................ucagugcauUacagaacuug.......    2          1          seq
..............................................ucagugcGugacagaacuug.......    1          1          seq
..............................................ucagugcaugacagaacuuC.......    1          1          seq
..............................................ucagugcaugacagaacuuA.......    5          1          seq
..............................................ucagugcaugacaUaacuug.......    1          1          seq
..............................................ucCgugcaugacagaCcuug.......    2          2          seq
..............................................ucagugcaugaAagaacuug.......    1          1          seq
..............................................ucagugcaugCcCgaacuugg......    3          2          seq
..............................................ucCgugcaugacagaCcuugg......    249        2          seq
..............................................ucaguUcauAacagaacuugg......    1          2          seq
..............................................uUagugcaugacagaacuugg......    247        1          seq
..............................................ucaCugcaugacagaacuugA......    1          2          seq
..............................................ucaguUUaugacagaacuugg......    2          2          seq
..............................................ucagugGaugacagCacuugg......    1          2          seq
..............................................uAGgugcaugacagaacuugg......    1          2          seq
..............................................uGagugcaugacagGacuugg......    1          2          seq
..............................................ucaCugcaugacagaaUuugg......    1          2          seq
..............................................ucaCuUcaugacagaacuugg......    1          2          seq
..............................................ucaguUcaugacagaacCugg......    1          2          seq
..............................................uGGgugcaugacagaacuugg......    1          2          seq
..............................................ucagugcaugacagaacCugg......    38         1          seq
..............................................ucagugcaCgacagaacuugg......    24         1          seq
..............................................ucagugcauAacagaacuugg......    39         1          seq
..............................................uGagugcaugacagaacuugg......    1236       1          seq
..............................................uUagugcaugacagaacuugU......    2          2          seq
..............................................ucaCugcaugacagaacuugg......    240        1          seq
..............................................ucaguUcaugacagaacuuAg......    1          2          seq
..............................................ucGCugcaugacagaacuugg......    1          2          seq
..............................................ucagugcaugaAagaCcuugg......    121        2          seq
..............................................uAagugcaugacagaaUuugg......    1          2          seq
..............................................ucagGgcaugacagaacuugU......    3          2          seq
..............................................uGagugcaugacagaacCugg......    1          2          seq
..............................................uGagugGaugacagaacuugg......    2          2          seq
..............................................ucagugcaugacagaacuuUA......    1          2          seq
..............................................ucagugcauUacagaacuugU......    1          2          seq
..............................................ucaUugcauUacagaacuugg......    2          2          seq
..............................................ucGgugAaugacagaacuugg......    2          2          seq
..............................................ucagugcaugaAUgaacuugg......    1          2          seq
..............................................ucaguUcaugacagaUcuugg......    1          2          seq
..............................................CcagugcaugacagaacuugA......    1          2          seq
..............................................ucaguUcaugacagaacuugA......    6          2          seq
..............................................ucagugcCugacagaacuugg......    9          1          seq
..............................................ucagugcaugaAagaacuugg......    99         1          seq
..............................................ucaguCcaugacagaacCugg......    1          2          seq
..............................................ucaguUcaugaUagaacuugg......    1          2          seq
..............................................ucagGgcaugacagaacuuCg......    2          2          seq
..............................................ucagugcaugacUgaCcuugg......    1          2          seq
..............................................AcagugcCugacagaacuugg......    1          2          seq
..............................................ucGgugcaugacagaacuugg......    310        1          seq
..............................................ucGgugcaugacagaacuugU......    2          2          seq
..............................................ucagCgcaugacagaacuugg......    116        1          seq
..............................................ucaCugcaugacagaCcuugg......    1          2          seq
..............................................ucagugcaugacagGacuugg......    30         1          seq
..............................................ucagugcaugacagaacuCgU......    1          2          seq
..............................................ucCgugcaugacagaaAuugg......    1          2          seq
..............................................ucagCgcaugacaUaacuugg......    1          2          seq
..............................................ucagugcaugacaAaacuugg......    156        1          seq
..............................................ucaUuUcaugacagaacuugg......    1          2          seq
..............................................ucagugcaugaAagaacuugU......    2          2          seq
```

```
                                              mmu-miR-152-3p
       mmu-miR-152-5p
ccgggccuagguucugugauacacuccgacucgggcucuggagcagucagugcaugacagaacuugggcccgg

..............................................ucagGgcaugacagaacuuUg......    1          2          seq
..............................................AcCgugcaugacagaacuugg......    2          2          seq
..............................................ucagugcaugacagaaAuuUg......    1          2          seq
..............................................ucagugcaugaUaUaacuugg......    1          2          seq
..............................................ucagugcaugacGgCacuugg......    1          2          seq
..............................................AcagugcaugacaUaacuugg......    1          2          seq
..............................................Acagugcaugacagaacuugg......    64         1          seq
..............................................ucaguUAaugacagaacuugg......    4          2          seq
..............................................ucagugcaugacagaCcuugU......    1          2          seq
..............................................ucagugUaugacUgaacuugg......    1          2          seq
..............................................ucagugUaugacaUaacuugg......    1          2          seq
..............................................ucagugcaugaUagaCcuugg......    88         2          seq
..............................................uAagugcaugacagaacuugU......    4          2          seq
..............................................ucagugcaugGcagaCcuugg......    1          2          seq
..............................................ucagugcaugacUgaaUuugg......    2          2          seq
..............................................ucagugGaugacagaacuugg......    13         1          seq
..............................................ucagugcaugacagaUcuugg......    46         1          seq
..............................................uGagugcaugUcagaacuugg......    1          2          seq
..............................................ucagugcaugGcagaacuugg......    29         1          seq
..............................................ucagGgcaugacagaacuugg......    243        1          seq
..............................................ucGgugcaugacagaCcuugg......    2          2          seq
..............................................uAaCugcaugacagaacuugg......    1          2          seq
..............................................ucaAugcaugaAagaacuugg......    1          2          seq
..............................................ucaguCcaugacagaacuugA......    1          2          seq
..............................................ucagCgcaugacagaacuugA......    1          2          seq
..............................................ucagugcaugacaCaacuugU......    2          2          seq
..............................................ucCgugcaugacagaGcuugg......    10         2          seq
..............................................ucagAgcaugacagaacuugA......    1          2          seq
..............................................ucagugcaugacCgaacCugg......    1          2          seq
..............................................Gcagugcaugacagaacuugg......    16         1          seq
..............................................ucagugcaugaUagaacuugA......    1          2          seq
..............................................ucagugcaugacUgaacuugg......    381        1          seq
..............................................ucCgugcaugacagaacuuUg......    1          2          seq
..............................................ucagugcaugacCgaacuugg......    699        1          seq
..............................................ucagugcaugCcagaacuugg......    110        1          seq
..............................................ucagugAaugacagaacuugU......    1          2          seq
..............................................uUagugcaugacaUaacuugg......    1          2          seq
..............................................ucagugcaAgacagaacuugg......    12         1          seq
..............................................ucagugcaugacagaacuCUg......    1          2          seq
..............................................ucagugcGugacaUaacuugg......    1          2          seq
..............................................ucagugcaugacagaUcuugA......    1          2          seq
..............................................ucagugcaugacagaaUuugg......    31         1          seq
..............................................ucagugcaugacagaacuuUU......    6          2          seq
..............................................ucagugcaugacagaacuuCU......    1          2          seq
..............................................ucagugcGugacagaacuugg......    27         1          seq
..............................................ucagugcaugacagaacuAgg......    7          1          seq
..............................................uGagugcaugacaCaacuugg......    1          2          seq
..............................................ucCguCcaugacagaacuugg......    1          2          seq
..............................................ucagugcaugacGgaacuugU......    1          2          seq
..............................................ucagAgcaugacagaGcuugg......    1          2          seq
..............................................ucagCgcaugacagaacuugC......    1          2          seq
..............................................ucCgCgcaugacagaacuugg......    1          2          seq
..............................................ucagugcaugCcagaacuugU......    1          2          seq
..............................................uGagugcaugacagaacuugA......    4          2          seq
..............................................ucagAgcaugacagaacuugg......    65         1          seq
..............................................ucagugcaugacGgaacuugA......    1          2          seq
..............................................ucagugcaugaUagaacuugg......    102        1          seq
..............................................ucCgugcaugacagaUcuugg......    46         2          seq
..............................................uGagAgcaugacagaacuugg......    3          2          seq
..............................................ucagCgcCugacagaacuugg......    1          2          seq
..............................................ucagGgcauAacagaacuugg......    1          2          seq
..............................................ucagugcaGgacagaacuugg......    7          1          seq
..............................................Ccagugcaugacagaacuugg......    58         1          seq
..............................................ucagugcaugCcagaCcuugg......    2          2          seq
..............................................ucagugcaugacaUaacuugU......    3          2          seq
..............................................ucaguUcaugacagaacuugU......    3          2          seq
..............................................uACgugcaugacagaacuugg......    1          2          seq
..............................................ucagugcGugacagaacuugC......    1          2          seq
..............................................ucagugcGugacUgaacuugg......    1          2          seq
```

```
                                              mmu-miR-152-3p
       mmu-miR-152-5p
ccgggccuagguucugugauacacuccgacucgggcucuggagcagucagugcaugacagaacuugggcccgg

..............................................ucagugcaugacGgaacuugg......    168       1          seq
..............................................ucGguUcaugacagaacuugg......    1         2          seq
..............................................ucagugcUugacagCacuugg......    1         2          seq
..............................................ucagugcCugacaUaacuugg......    1         2          seq
..............................................ucaguCcaugacagaacuugg......    229       1          seq
..............................................ucaguUcaugacagaacuugg......    828       1          seq
..............................................uGCgugcaugacagaacuugg......    1         2          seq
..............................................ucagugcaugacagaacGugg......    16        1          seq
..............................................ucUgugcaugacagaacuugg......    30        1          seq
..............................................ucagugcaugaGagaCcuugg......    221       2          seq
..............................................ucCgugcaugacagaacuugg......    1270      1          seq
..............................................ucagugUaugacagaacuugU......    1         2          seq
..............................................uGagGgcaugacagaacuugg......    6         2          seq
..............................................uGagugcauUacagaacuugg......    1         2          seq
..............................................ucaguAcaugacagaacuugg......    157       1          seq
..............................................ucCgugcaugacagaacuAgg......    1         2          seq
..............................................ucagugUaugacagaacuugg......    58        1          seq
..............................................uGaguUcaugacagaacuugg......    1         2          seq
..............................................ucCgugcCugacagaacuugg......    3         2          seq
..............................................ucagugAaugacagaacuugg......    34        1          seq
..............................................ucagugcUugacagaacuugg......    8         1          seq
..............................................ucagugcaugacagaacuugU......    950       1          seq
..............................................ucagugcaugacCgaacuugA......    2         2          seq
..............................................ucagugcaugacaUaacuugA......    6         2          seq
..............................................ucagugcaugacagaCcuuAg......    1         2          seq
..............................................ucagugcaugacCCaacuugg......    1         2          seq
..............................................ucCgugcaugCcagaacuugg......    1         2          seq
..............................................ucagugcauCacagaacuugg......    35        1          seq
..............................................ucagugcaugacagaAuugg......    12        1          seq
..............................................ucCgugcaugaUagaacuugg......    1         2          seq
..............................................ucaUugUaugacagaacuugg......    1         2          seq
..............................................ucUCugcaugacagaacuugg......    1         2          seq
..............................................ucagugcaugacagaacuCgg......    48        1          seq
..............................................ucagugcauUacagaacuugg......    91        1          seq
..............................................ucagugcaugacaUaacuugg......    721       1          seq
..............................................ucagugcaugacGUaacuugg......    1         2          seq
..............................................ucCgugcaugacagaacuugU......    2         2          seq
..............................................ucagugcaugacagaacuuCg......    122       1          seq
..............................................ucagugcaugacGAaacuugg......    1         2          seq
..............................................ucagugcaugacaAaacuugU......    2         2          seq
..............................................uAagugcaugacagaacuugg......    456       1          seq
..............................................ucaUugcaugacagaacuugg......    210       1          seq
..............................................ucagugAaugacagGacuugg......    1         2          seq
..............................................ucaAugcaugacagaacuugg......    85        1          seq
..............................................ucagugcaugacagaacuuAU......    1         2          seq
..............................................ucagugcaugacaCaacuugg......    248       1          seq
..............................................ucagugcaugacGgaCcuugg......    1         2          seq
..............................................ucCgugcaugacaUaacuugg......    1         2          seq
..............................................ucagCUcaugacagaacuugg......    5         2          seq
..............................................ucCgugGaugacagaacuugg......    2         2          seq
..............................................AcagugcaugacagaCcuugg......    1         2          seq
..............................................ucagAgcaugaAagaacuugg......    1         2          seq
..............................................ucaguCcaugacagaacAugg......    1         2          seq
..............................................ucCgugAaugacagaacuugg......    5         2          seq
..............................................ucagugcaugaUCgaacuugg......    2         2          seq
..............................................ucagugcaugacagaacuuUg......    531       1          seq
..............................................ucagugcaugacagaaGuugg......    3         1          seq
..............................................ucagugcaugUcagaacuugg......    26        1          seq
..............................................ucagugcaCgacaUaacuugg......    1         2          seq
..............................................uGaguAcaugacagaacuugg......    1         2          seq
..............................................ucagugcaugaGagaUcuugg......    2         2          seq
..............................................ucagGgcaugacagaaUuugg......    1         2          seq
..............................................ucagugcaugacagaGcuugg......    24        1          seq
..............................................ucagugAaugacagaaAuugg......    1         2          seq
..............................................ucagugcaugacagaacuugg......    169039    0          seq
..............................................ucagugcaugacCgaacuugU......    2         2          seq
..............................................ucagGgcaugCcagaacuugg......    1         2          seq
..............................................ucagugcaugacagaacuGgA......    1         2          seq
..............................................ucaguUcaugacagaCcuugg......    1         2          seq
```

```
                                              mmu-miR-152-3p
       mmu-miR-152-5p
ccgggccuagguucugugauacacuccgacucgggcucuggagcagucagugcaugacagaacuugggcccgg

..............................................ucaUugcaugacagaacuugU......      2      2      seq
..............................................ucagugcaugaAagaUcuugg......      1      2      seq
..............................................ucagugcaugacagaCcuugg......      321    1      seq
..............................................ucaguUcaugacaAaacuugg......      1      2      seq
..............................................ucagGgcaugacagaacuGgg......      1      2      seq
..............................................ucagugcCugacagGacuugg......      1      2      seq
..............................................ucagugcaugacagaacAugg......      6      1      seq
..............................................uAagugAaugacagaacuugg......      2      2      seq
..............................................ucagugcaugacaUaacuugC......      1      2      seq
..............................................uUagugcaugacagGacuugg......      1      2      seq
..............................................CcCgugcaugacagaacuugg......      1      2      seq
..............................................ucaAugcaugacagaacGugg......      1      2      seq
..............................................ucagugcaugacagCacuugg......      24     1      seq
..............................................ucagugcauUacagaacuCgg......      1      2      seq
..............................................ucagGgcaugacUgaacuugg......      1      2      seq
..............................................uGagugcaugacagaacuugC......      1      2      seq
..............................................ucaAugcaugacagaacCugg......      1      2      seq
..............................................ucagugcaugacCgaacuGgg......      1      2      seq
..............................................ucCgugUaugacagaacuugg......      1      2      seq
..............................................ucaguAcaugacaUaacuugg......      1      2      seq
..............................................ucaCugcaugacCgaacuugg......      2      2      seq
..............................................ucagCgcaugacUgaacuugg......      1      2      seq
..............................................ucagugcaugacagaacuugA......      664    1      seq
..............................................ucagAgcaugacagaacuugU......      1      2      seq
..............................................ucagCgcaugacCgaacuugg......      1      2      seq
..............................................ucaguUGaugacagaacuugg......      1      2      seq
..............................................ucagugcaugacagaacuGgg......      10     1      seq
..............................................ucaguCcauAacagaacuugg......      1      2      seq
..............................................uAagugcaugacagaacuugA......      4      2      seq
..............................................ucaCugcaugacagaacuugU......      2      2      seq
..............................................ucagGgcaugaAagaacuugg......      1      2      seq
..............................................uGagugcaugacagaacuugU......      8      2      seq
..............................................ucaguAcaugacagaacuugU......      1      2      seq
..............................................ucCgugcaugacagaacuugA......      5      2      seq
..............................................ucagugcaugacCgaacuCgg......      1      2      seq
..............................................ucagugcaugacagaacuGUg......      2      2      seq
..............................................ucaguAAaugacagaacuugg......      1      2      seq
..............................................ucGgugcaugacagGacuugg......      1      2      seq
..............................................ucaAugcaugacagaacuugA......      1      2      seq
..............................................ucaguCcaugacagaacuugU......      1      2      seq
..............................................ucaguUcaugacagaacuuUg......      3      2      seq
..............................................uAagugcaugaUagaacuugg......      1      2      seq
..............................................uUagugcaugacCgaacuugg......      1      2      seq
..............................................ucagugcaugaUagaacuugU......      1      2      seq
..............................................ucCCugcaugacagaacuugg......      2      2      seq
..............................................ucagugcauAacagaCcuugg......      1      2      seq
..............................................ucaUugcaugacCgaacuugg......      2      2      seq
..............................................ucagugcaugacagaacuuAA......      6      2      seq
..............................................ucagugcaugaGagaacuugg......      197    1      seq
..............................................ucGgGgcaugacagaacuugg......      1      2      seq
..............................................ucagugcaugacagUacuugg......      9      1      seq
..............................................ucagugcaugacagaacuugC......      41     1      seq
..............................................uUagugcaugacagaacuugA......      1      2      seq
..............................................ucagugcaugacagaacuuAg......      66     1      seq
..............................................ucagGgcaugacCgaacuugg......      1      2      seq
..............................................ucUgugcaugacagaacuugA......      1      2      seq
..............................................ucagugcaugacUgaacuugA......      2      2      seq
..............................................ucagugcauCacagaacuugU......      1      2      seq
..............................................ucaUugcauCacagaacuuggg.....      1      2      seq
..............................................ucagugcaCgacagaacuuUgg.....      1      2      seq
..............................................ucagugcCugacagaacuuggg.....      1      1      seq
..............................................ucagugcaugacagaCcuuggg.....      20     1      seq
..............................................Ccagugcaugacagaacuuggg.....      2      1      seq
..............................................ucagugcauAacagaacuuggU.....      3      2      seq
..............................................ucagugcaugCcagaacuuggU.....      10     2      seq
..............................................ucagugcaGgacagaacuuggA.....      2      2      seq
..............................................ucagugcaugacaUaacuuggA.....      136    2      seq
..............................................ucagugcaugCcagaacuuggA.....      13     2      seq
..............................................ucagugcGugacagaacuuggU.....      3      2      seq
```

```
                                              mmu-miR-152-3p
       mmu-miR-152-5p
ccgggccuagguucugugauacacuccgacucgggcucuggagcagucagugcaugacagaacuugggcccgg

..............................................ucagugcaugaAagaaAuuggg.....    1        2          seq
..............................................ucagugcaugacaCaacuuggU.....    24       2          seq
..............................................ucagugcauAacagaacuuggA.....    5        2          seq
..............................................ucagugcaugacagaCcuuggU.....    38       2          seq
..............................................ucagugcauUacagaacuuggU.....    25       2          seq
..............................................ucaUugcaugacagaacuuggA.....    23       2          seq
..............................................ucagGgcaugacagaacuuggA.....    40       2          seq
..............................................ucagugcaugacagaacuuCgg.....    6        1          seq
..............................................ucagugcaGgacagaacuuggg.....    1        1          seq
..............................................ucagugcaugacagaacuGggg.....    1        1          seq
..............................................uGagugcaugacagaacuuggA.....    208      2          seq
..............................................ucagugcaugGcagaacuuggA.....    3        2          seq
..............................................ucagugcaugacaUaacuuggU.....    99       2          seq
..............................................CcagugcaugacagaacuuggU.....    6        2          seq
..............................................uACgugcaugacagaacuuggg.....    1        2          seq
..............................................ucCgugcaugacagaacuuggA.....    202      2          seq
..............................................ucagugcaugaUagaCcuuggg.....    4        2          seq
..............................................ucaguUcaugacagaacuuggg.....    17       1          seq
..............................................ucagugcauUacagaacuuggg.....    4        1          seq
..............................................ucagugcaugacagaacuugAU.....    1        2          seq
..............................................ucaAugcaugacagaacuuggA.....    14       2          seq
..............................................ucaAugcaugacagaacuuggU.....    8        2          seq
..............................................ucagugcaugacagaaAuuggA.....    1        2          seq
..............................................ucagugcaugaGagaCcuuggg.....    11       2          seq
..............................................ucagugcaugacaCaacuuggA.....    43       2          seq
..............................................ucagugcaugacagaacuuggg.....    6790     0          seq
..............................................uUagugcaugacagaacuuggg.....    14       1          seq
..............................................ucaguCcaugacagaacuuggg.....    13       1          seq
..............................................ucagugcaugacaUaacuuggg.....    30       1          seq
..............................................ucagugcaugacagaacuuggA.....    25235    1          seq
..............................................ucagugcaugacagaacuugUA.....    26       2          seq
..............................................ucagugcaugGcagaacuuggU.....    2        2          seq
..............................................AcagugcaugacagaacuuggA.....    12       2          seq
..............................................ucaUugcaugacagaacuuggg.....    6        1          seq
..............................................ucagugcauUacagaacuuggA.....    12       2          seq
..............................................uAagugcaugacagaacuuggg.....    20       1          seq
..............................................ucagugcaugacagaacuAggg.....    1        1          seq
..............................................ucaguUcaugacagaacuuggU.....    98       2          seq
..............................................ucagugcaugacCgaacuuggA.....    122      2          seq
..............................................ucaUugcaugacagaacuuggU.....    24       2          seq
..............................................ucagugcaugacagaGcuuggA.....    2        2          seq
..............................................ucagugcaugaAagaacuuggA.....    12       2          seq
..............................................ucaCugcaugacagaacuuggg.....    9        1          seq
..............................................ucGgugcaugacagaacuuggg.....    6        1          seq
..............................................ucagugcaugacagaacuuggC.....    94       1          seq
..............................................ucagugcaugacGgaacuuggU.....    16       2          seq
..............................................ucagugcaugacagaacuugCU.....    1        2          seq
..............................................ucagugcaugacagaacuGggA.....    1        2          seq
..............................................ucagugUaugacagaacuuggg.....    6        1          seq
..............................................ucGgugcaugacagaacuuggU.....    30       2          seq
..............................................ucagCgcaugacagaacuuggg.....    5        1          seq
..............................................ucagAgcaugacagaacuuggg.....    4        1          seq
..............................................ucGgugcaugacagaacuuggA.....    45       2          seq
..............................................ucagugcaAgacagaacuuggU.....    1        2          seq
..............................................Acagugcaugacagaacuuggg.....    4        1          seq
..............................................ucaguAcaugacagaacuuggU.....    17       2          seq
..............................................ucagugcaugacagaacGuggA.....    2        2          seq
..............................................ucagugcaugacaCaacuuggg.....    10       1          seq
..............................................ucagugcaugacagaacuAggU.....    2        2          seq
..............................................ucagugcauCacCgaacuuggg.....    1        2          seq
..............................................ucagugcaugacaAaacuuggg.....    5        1          seq
..............................................ucagugcaugacagaaUuuggg.....    1        1          seq
..............................................ucagAgcaugacagaacuuggU.....    6        2          seq
..............................................ucagugcaugacUgaacuuggg.....    19       1          seq
..............................................ucagugcaugacagaaGuuggA.....    1        2          seq
..............................................ucUgugcaugacagaacuuggg.....    2        1          seq
..............................................ucagugcaugacagaacuugAA.....    32       2          seq
..............................................ucagugcaugaGagaacuuggg.....    6        1          seq
..............................................ucagAgcaugacagaacuuggA.....    10       2          seq
```

```
                                              mmu-miR-152-3p
       mmu-miR-152-5p
ccgggccuagguucugugauacacuccgacucgggcucuggagcagucagugcaugacagaacuugggcccgg

..............................................ucagugcaugGcagaacuuggg.....    1      1      seq
..............................................ucagugcCugacagaacuuggU.....    1      2      seq
..............................................ucGgugAaugacagaacuuggg.....    1      2      seq
..............................................CcagugcaugacagaacuuggA.....    9      2      seq
..............................................ucUgugcaugacagaacuuggU.....    3      2      seq
..............................................ucagugcaugUcagaacuuggA.....    7      2      seq
..............................................ucagugcaugacagaacuCggg.....    1      1      seq
..............................................ucagugAaugacagaacuuggU.....    2      2      seq
..............................................ucCgugcaugacagaacuuggg.....    51     1      seq
..............................................ucagugcaugacagaacuCggA.....    5      2      seq
..............................................ucagugcauCacagaacuuggg.....    2      1      seq
..............................................ucagCgcaugacagaacuuggA.....    15     2      seq
..............................................ucagugcCugacagaacuuggA.....    1      2      seq
..............................................ucagugcaugacagaaUuuggU.....    1      2      seq
..............................................ucagugcaugacagaacuugUg.....    1      1      seq
..............................................ucCgugcaugacagaUcuuggg.....    2      2      seq
..............................................ucagGgcaugacagaacuuggg.....    13     1      seq
..............................................ucagugcaugacagaacuuUgg.....    26     1      seq
..............................................ucagugcaugCcagaacuuggg.....    6      1      seq
..............................................ucaguCcaugacagaacuuggU.....    25     2      seq
..............................................uUagugcaugacagaacuuggA.....    35     2      seq
..............................................ucagugcaugacagGacuuggU.....    2      2      seq
..............................................ucagugcaugacagaacuugUU.....    31     2      seq
..............................................ucagugcaugacagaacuuCgU.....    9      2      seq
..............................................ucagugcaugacagaacuuAgU.....    12     2      seq
..............................................ucagugcaugacagaacuuggU.....    17208  1      seq
..............................................ucagugcaugaGagaacuuggU.....    16     2      seq
..............................................ucagugcaugaUagaacuuggU.....    13     2      seq
..............................................ucagugcaugacagCacuuggU.....    5      2      seq
..............................................ucagugcaugacagaUcuuggA.....    6      2      seq
..............................................ucagugcaugacagaacuAggA.....    1      2      seq
..............................................ucagugcaCgacagaacuuggA.....    6      2      seq
..............................................ucagugcaugacGgaacuuggg.....    4      1      seq
..............................................ucaCugcaugacagaacuuggU.....    22     2      seq
..............................................uAagugcaugacagaacuuggA.....    79     2      seq
..............................................ucagugAaugacagaacuuggA.....    3      2      seq
..............................................ucagugUaugacagaacuuggA.....    2      2      seq
..............................................ucagugcaugacCgaacuuggg.....    39     1      seq
..............................................ucagugcaugacCgaacuuggU.....    78     2      seq
..............................................ucagugcaugacUgaacuuggA.....    54     2      seq
..............................................ucCgugcaugacagaacuuggU.....    117    2      seq
..............................................uAagugcaugacagaacuuggU.....    52     2      seq
..............................................ucagugcaugaUagaacuuggg.....    8      1      seq
..............................................ucagugGaugacagaacuuggA.....    3      2      seq
..............................................ucagugcaugacagaCcuuggA.....    38     2      seq
..............................................GcagugcaugacagaacuuggU.....    3      2      seq
..............................................ucagugcaugaGagaacuuggA.....    26     2      seq
..............................................ucagugcaugacagaacuugAg.....    3      1      seq
..............................................ucagugcaugacagaacuuCgA.....    14     2      seq
..............................................ucaAugcaugacagaacuuggg.....    6      1      seq
..............................................uUagugcaugacagaacuuggU.....    26     2      seq
..............................................ucagugcaugacagaGcuuggU.....    2      2      seq
..............................................ucagugcauAacagaacuuggg.....    1      1      seq
..............................................ucagugcaugacagaUcuuggg.....    3      1      seq
..............................................ucagugcaugacagaacuugAC.....    1      2      seq
..............................................ucagugcaugacagaacuugCg.....    3      1      seq
..............................................ucagugcaugacagaacuuUgA.....    88     2      seq
..............................................ucagugcaugUcagaacuuggU.....    4      2      seq
..............................................ucagugcaugacagGacuuggA.....    1      2      seq
..............................................ucaguUcaugacagaacuuggA.....    111    2      seq
..............................................ucagugcaugaAagaacuuggg.....    5      1      seq
..............................................ucagugcaugacagaUcuuggU.....    5      2      seq
..............................................ucagugcaCUacagaacuuggg.....    3      2      seq
..............................................ucagugcaugacagaacuuAgA.....    13     2      seq
..............................................ucagugcaugacagaacuuAgg.....    2      1      seq
..............................................ucagugcaugaAagaCcuuggg.....    9      2      seq
..............................................ucaguAcaugacagaacuuggg.....    5      1      seq
..............................................ucagugcaugacaUaacuuggC.....    2      2      seq
..............................................ucagugcaugacagaacCuggA.....    4      2      seq
```

```
                                              mmu-miR-152-3p
       mmu-miR-152-5p
ccgggccuagguucugugauacacuccgacucgggcucuggagcagucagugcaugacagaacuugggcccgg

..............................................ucagugcaugacagaGcuuggg.....    1          1          seq
..............................................ucaCugcaGgacagaacuuggg.....    1          2          seq
..............................................ucagugcaugacagaaAuuggU.....    2          2          seq
..............................................ucagugcaugacagaaUuuggA.....    5          2          seq
..............................................ucUgugcaugacagaacuuggA.....    4          2          seq
..............................................ucagugcaugacaAaacuuggA.....    23         2          seq
..............................................ucaCugcaugacagaacuuggA.....    35         2          seq
..............................................ucagugcaugacagaacCuggg.....    2          1          seq
..............................................ucaguAcaugacagaacuuggA.....    23         2          seq
..............................................ucaguCcaugacagaacuuggA.....    26         2          seq
..............................................ucagCgcaugacagaacuuggU.....    10         2          seq
..............................................uGagugcaugacagaacuuggU.....    116        2          seq
..............................................uGagugcaugacagaacuuggg.....    56         1          seq
..............................................ucagugcaugUcagaacuuggg.....    1          1          seq
..............................................ucagugcaugacGgaacuuggA.....    26         2          seq
..............................................ucagugcaugaUagaacuuggA.....    13         2          seq
..............................................GcagugcaugacagaacuuggA.....    2          2          seq
..............................................ucagugcaugacagaacuugCA.....    7          2          seq
..............................................ucagugcGugacagaacuuggA.....    3          2          seq
..............................................AcagugcaugacagaacuuggU.....    9          2          seq
..............................................ucagugcauCacagaacuuggA.....    7          2          seq
..............................................ucagugcaCgacagaacuuggg.....    6          1          seq
..............................................ucagGgcaugacagaacuuggU.....    52         2          seq
..............................................ucagugcaCgacagaacuuggU.....    3          2          seq
..............................................ucCgugcaugacagaacuuggC.....    1          2          seq
..............................................ucCgugcaugacagaCcuuggg.....    10         2          seq
..............................................ucagugcaugaAagaacuuggU.....    7          2          seq
..............................................ucagugUaugacagaacuuggU.....    2          2          seq
..............................................ucagugcaugacagaacuGggU.....    1          2          seq
..............................................ucagugcaugacaAaacuuggU.....    16         2          seq
..............................................ucagugGaugacagaacuuggU.....    1          2          seq
..............................................ucagugcaugacUgaacuuggU.....    42         2          seq
..............................................ucagugcaugacagCacuuggA.....    2          2          seq
..............................................ucagugcaugacagaacuuUggU....    5          2          seq
..............................................ucagugcaugacagaacuuAggU....    1          2          seq
..............................................ucagCgcaugacagaacuugggA....    3          2          seq
..............................................ucagugcaugacagCacuugggU....    2          2          seq
..............................................ucUgugcaugacagaacuugggA....    1          2          seq
..............................................ucagugcaugacagaacuugggc....    11         0          seq
..............................................uAagugcaugacagaacuugggU....    2          2          seq
..............................................ucagugcaugacagaacuuggAU....    458        2          seq
..............................................ucGgugcaugacagaacuugggU....    2          2          seq
..............................................ucagugcaugacagaacuuUggA....    9          2          seq
..............................................ucaUugcaugacagaacuugggA....    1          2          seq
..............................................ucagugcaugacCgaacuugggU....    4          2          seq
..............................................ucagugcaugacagaacuugUgA....    3          2          seq
..............................................ucagugcaugacagaacuugggU....    943        1          seq
..............................................ucagugcaugacagaacuugUUc....    1          2          seq
..............................................uGagugcaugacagaacuugggA....    10         2          seq
..............................................uGagugcaugacagaacuugggc....    1          1          seq
..............................................ucaguAcaugacagaacuugggA....    3          2          seq
..............................................ucaguCcaugacagaacuugggA....    3          2          seq
..............................................uUagugcaugacagaacuugggU....    4          2          seq
..............................................ucagugcaugUcagaacuugggU....    1          2          seq
..............................................ucagugcaugacagaacuuggAc....    76         1          seq
..............................................ucaCugcaugacagaacuugggU....    1          2          seq
..............................................ucagugcaugacagaacuGgggU....    1          2          seq
..............................................ucaguUcaugacagaacuugggU....    7          2          seq
..............................................ucaguAcaugacagaacuuggUc....    1          2          seq
..............................................ucagugcaugacagaUcuugggU....    1          2          seq
..............................................ucagugcaugacagaacuuggCA....    7          2          seq
..............................................ucagugcaugacagGacuugggA....    1          2          seq
..............................................ucaguUcaugacagaacuugggA....    6          2          seq
..............................................ucagugcaugaAagaacuugggA....    2          2          seq
..............................................ucGgugcaugacagaacuuggCc....    1          2          seq
..............................................ucGgugcaugacagaacuugggA....    5          2          seq
..............................................uUagugcaugacagaacuugggA....    2          2          seq
..............................................ucagGgcaugacagaacuugggU....    3          2          seq
..............................................ucagugcaugacUgaacuugggA....    6          2          seq
```

```
                                              mmu-miR-152-3p
       mmu-miR-152-5p
ccgggccuagguucugugauacacuccgacucgggcucuggagcagucagugcaugacagaacuugggcccgg

..............................................ucagugcaugacaUaacuugggU....   3      2       seq
..............................................ucagugcaugacagaacuugUgU....   1      2       seq
..............................................ucaCugcaugacagaacuugggA....   1      2       seq
..............................................ucagugcaugacCgaacuugggA....   7      2       seq
..............................................ucagugcaugacagaacuugggG....   8      1       seq
..............................................ucagugcaugaGagaacuugggA....   4      2       seq
..............................................ucagugcaugacagaacuuCggA....   1      2       seq
..............................................ucagugcaugacagaacuuggUU....   959    2       seq
..............................................ucagugcaugaUagaacuugggU....   1      2       seq
..............................................ucagGgcaugacagaacuugggA....   3      2       seq
..............................................ucaAugcaugacagaacuugggA....   2      2       seq
..............................................CcagugcaugacagaacuugggA....   1      2       seq
..............................................ucagugcaugacagaacuuggUc....   10     1       seq
..............................................ucagugcaugacUgaacuugggU....   2      2       seq
..............................................ucUgugcaugacagaacuugggU....   1      2       seq
..............................................ucagugcaugacagaacuuggCU....   10     2       seq
..............................................ucagAgcaugacagaacuugggA....   2      2       seq
..............................................ucagugcaugacagaCcuugggA....   4      2       seq
..............................................ucagugcaugacaCaacuugggA....   1      2       seq
..............................................uGagugcaugacagaacuugggU....   12     2       seq
..............................................ucagugcaugacagaacuuggUA....   957    2       seq
..............................................ucagugcaugacagaGcuugggU....   1      2       seq
..............................................ucagugcaugCcagaacuugggU....   1      2       seq
..............................................ucagugcaugacagaacuugggA....   1757   1       seq
..............................................ucagugcaugacGgaacuugggA....   3      2       seq
..............................................ucagugcaugacagaacuuggAG....   203    2       seq
..............................................ucagugcaugacagaacuCgggU....   1      2       seq
..............................................ucagugcaugacagaacuugAAc....   1      2       seq
..............................................ucCgugcaugacagaacuugggU....   8      2       seq
..............................................ucagugcaugacagaacuuggUG....   29     2       seq
..............................................ucagugcaugaAagaacuugggU....   2      2       seq
..............................................ucagAgcaugacagaacuugggU....   1      2       seq
..............................................ucagugcaugacagaacuuAggA....   2      2       seq
..............................................ucagugcaugaGagaacuugggU....   1      2       seq
..............................................ucagugcaugacagaCcuugggU....   2      2       seq
..............................................CcagugcaugacagaacuugggU....   1      2       seq
..............................................ucagugcaugacagaacuuggAA....   1162   2       seq
..............................................ucagugcaugacagaacuuggCG....   1      2       seq
..............................................ucagugcUugacagaacuugggA....   1      2       seq
..............................................ucagugcaugacagaacuuCggU....   1      2       seq
..............................................ucCgugcaugacagaacuugggA....   14     2       seq
..............................................ucagugcaugacaCaacuugggU....   1      2       seq
..............................................ucagugcaugacaUaacuugggA....   3      2       seq
..............................................uAagugcaugacagaacuugggA....   5      2       seq
..............................................ucagugcaugacagaacuugggUc...   1      1       seq
..............................................ucagugcaugacagaacuuggUUc...   1      2       seq
..............................................ucagugcaugacagaacuuggAAc...   63     2       seq
..............................................ucagugcaugacagaacuugggAc...   18     1       seq
..............................................ucagugcaugacagaacuuggAUc...   3      2       seq
..............................................ucagugcaugacagaacuuggUcU...   9      2       seq
..............................................ucagugcaugacagaacuuggUcA...   1      2       seq
..............................................ucGgugcaugacagaacuugggAc...   1      2       seq
..............................................ucagugcaugacagaacuuggAcc...   2      1       seq
..............................................ucagugcaugacagaacuugggGA...   1      2       seq
..............................................ucagugcaugacagaacuugggUA...   47     2       seq
..............................................ucagugcaugacagaacuuggAcA...   10     2       seq
..............................................ucagugcaugacagaacuugggAU...   169    2       seq
..............................................ucagugcaugacagaacuuggUcG...   2      2       seq
..............................................ucagugcaugacagaacuugggUU...   109    2       seq
..............................................ucagugcaugacagaacuugggAA...   141    2       seq
..............................................ucagugcaugacagaacuugggGc...   1      1       seq
..............................................ucagugcaugacagaacuuggUAc...   15     2       seq
..............................................uGagugcaugacagaacuugggAc...   1      2       seq
..............................................ucagugcaugacagaacuugggUG...   2      2       seq
..............................................ucagugcaugacagaacuugggAG...   42     2       seq
..............................................ucagugcaugacagaacuuggCAc...   1      2       seq
..............................................ucagugcaugacagaacuugggGU...   1      2       seq
..............................................ucagugcaugacagaacuugggUAc..   3      2       seq
..............................................ucagugcaugacagaacuugggAAc..   15     2       seq
```

```
                                              mmu-miR-152-3p
       mmu-miR-152-5p
ccgggccuagguucugugauacacuccgacucgggcucuggagcagucagugcaugacagaacuugggcccgg

..............................................ucagugcaugacagaacuuggAcGc..    5      2      seq
..............................................ucagugcaugacagaacuugggcAU..    2      2      seq
..............................................ucagugcaugacagaacuugggAcA..    3      2      seq
..............................................ucagugcaugacagaacuugggcAA..    1      2      seq
..............................................ucagugcaugacagaacuugggAcU..    2      2      seq
..............................................ucagugcaugacagaacuuggUcUcg.    1      2      seq
...............................................cagugcaugacagaacuu........    2      0      seq
...............................................cagGgcaugacagaacuug.......    2      1      seq
...............................................cagugcaugacagaacuuU.......    1      1      seq
...............................................cagugcaugacagaacuug.......    3      0      seq
...............................................cagugcCugacagaacuug.......    1      1      seq
...............................................cagugcaugacagaacuugU......    26     1      seq
...............................................cagGgcaugacUgaacuugg......    2      2      seq
...............................................cagGgcaugacCgaacuugg......    2      2      seq
...............................................cagGgcaugacagaaUuugg......    2      2      seq
...............................................cagugGaugCcagaacuugg......    1      2      seq
...............................................cagugAaugacagaacuugg......    14     1      seq
...............................................cagGgcCugacagaacuugg......    3      2      seq
...............................................cagugcaugacaUaacuugg......    2      1      seq
...............................................cagGgcaugacaUaacuugg......    1      2      seq
...............................................cagugcaugCcagaacuugg......    14     1      seq
...............................................cagugcaugacaAaacuugg......    2      1      seq
...............................................cCgugcaugacagaacuugU......    1      2      seq
...............................................cagugcaugacagGacuugg......    3      1      seq
...............................................Aagugcaugacagaacuugg......    2      1      seq
...............................................cagugGCugacagaacuugg......    1      2      seq
...............................................cagugcaugacagUacuugg......    17     1      seq
...............................................cagugcUugacagaacuugg......    1      1      seq
...............................................cagGgcaugCcagaacuugg......    1      2      seq
...............................................cagugcaugacaCaacuugg......    5      1      seq
...............................................AagugcaCgacagaacuugg......    1      2      seq
...............................................cagGgAaugacagaacuugg......    1      2      seq
...............................................cagugcaugCcaCaacuugg......    1      2      seq
...............................................cagugcaugacagaGcuugg......    2      1      seq
...............................................cagugcaugacagaacCugg......    1      1      seq
...............................................cGgugcaugacagaacuugg......    27     1      seq
...............................................cagugUaugacagaacuugg......    16     1      seq
...............................................cagGgcaugacagGacuugg......    1      2      seq
...............................................cUgugcaugacagaacuugg......    4      1      seq
...............................................cagGgcaugacagaacuCgg......    1      2      seq
...............................................cagGgUaugacagaacuugg......    4      2      seq
...............................................cagugcaugacagaaUuugg......    4      1      seq
...............................................cagugcaugacGgaacuugg......    1      1      seq
...............................................cagugcaugCcagaacuugA......    1      2      seq
...............................................cagugcaugacagCacuugg......    2      1      seq
...............................................cagGgcaugacagaacuugg......    256    1      seq
...............................................cagGgcaugacaCaacuugg......    2      2      seq
...............................................cagAgcaugacagaacuugg......    1      1      seq
...............................................cagugcaugacagaacuugA......    10     1      seq
...............................................caCugcaugacagaacuugg......    2      1      seq
...............................................caguUcaugacagaacuugg......    2      1      seq
...............................................UGgugcaugacagaacuugg......    1      2      seq
...............................................cagugcaugacagaacuuUg......    1      1      seq
...............................................cagGgcaugacGgaacuugg......    9      2      seq
...............................................cagugGaugacagaacuugg......    10     1      seq
...............................................caguAcaugacagaacuugg......    1      1      seq
...............................................cagugcaugacagaacuugg......    2482   0      seq
...............................................cagGgcaugacagaaAuugg......    1      2      seq
...............................................caCGgcaugacagaacuugg......    1      2      seq
...............................................cagCgcaugacagaacuugg......    4      1      seq
...............................................cagugcaugacagaacuGgg......    1      1      seq
...............................................Uagugcaugacagaacuugg......    4      1      seq
...............................................cagGgcaugacagaacuugU......    1      2      seq
...............................................cagugcaugacagaacuuAA......    1      2      seq
...............................................cagugcaugacagaaAuugg......    4      1      seq
...............................................cagugcaCgacagaacuugg......    1      1      seq
...............................................cCgugcaugacagaacuugC......    1      2      seq
...............................................cagugAaugCcagaacuugg......    5      2      seq
...............................................cCgugcaugacagaacuugg......    18     1      seq
```

```
                                              mmu-miR-152-3p
       mmu-miR-152-5p
ccgggccuagguucugugauacacuccgacucgggcucuggagcagucagugcaugacagaacuugggcccgg

...............................................cagugUaugaGagaacuugg......      1      2      seq
...............................................cagugcaugaAagaacuuggA.....      1      2      seq
...............................................cagugcaugacagGacuuggA.....      1      2      seq
...............................................cagugcaugacagaaUuuggA.....      1      2      seq
...............................................cagugcaugacagaacuugAC.....      1      2      seq
...............................................cagugAaugacagaacuuggA.....      2      2      seq
...............................................cagAgcaugacagaacuuggA.....      1      2      seq
...............................................cagugcaugacagaacuuUgg.....      1      1      seq
...............................................cagugcaugacaUaacuuggA.....      1      2      seq
...............................................cCgugcaugacagaacuuggg.....      1      1      seq
...............................................cagugcaugacagUacuuggA.....      1      2      seq
...............................................cagCgcaugacagaacuuggU.....      1      2      seq
...............................................cagugUaugacagaacuuggA.....      4      2      seq
...............................................cUgugcaugacagaacuuggg.....      1      1      seq
...............................................cagugcauUacagaacuuggA.....      1      2      seq
...............................................cagGgcaugacagaacuuggg.....      6      1      seq
...............................................cagugcaugacagaacAuggA.....      1      2      seq
...............................................cagugcGugacagaacuuggU.....      1      2      seq
...............................................cagugcaugacaCaacuuggg.....      1      1      seq
...............................................UagugcaugacagaacuuggU.....      2      2      seq
...............................................cagugcaugacagCacuuggU.....      1      2      seq
...............................................cagugGaugacagaacuuggA.....      2      2      seq
...............................................caCugcaugacagaacuuggA.....      1      2      seq
...............................................cCgugcaugacagaacuuggU.....      1      2      seq
...............................................cagAgcaugacagaacuuggU.....      1      2      seq
...............................................cagugcCugacagaacuuggg.....      1      1      seq
...............................................cagugcaugCcagaacuuggA.....      1      2      seq
...............................................cagugcaugacagaacuuggA.....      454    1      seq
...............................................cagugGaugacagaacuuggg.....      2      1      seq
...............................................cagugcaugGcagaacuuggU.....      1      2      seq
...............................................cCgugcaugacagaacuuggA.....      2      2      seq
...............................................cagugAaugCcagaacuuggg.....      1      2      seq
...............................................cagugcaugacagUacuuggg.....      1      1      seq
...............................................cGgugcaugacagaacuuggA.....      3      2      seq
...............................................cagGgcaugacagaacuuggA.....      34     2      seq
...............................................cagugcaugacagaacuuggU.....      303    1      seq
...............................................caAugcaugacagaacuuggA.....      2      2      seq
...............................................caUugcaugacagaacuuggg.....      2      1      seq
...............................................cagugcaugacagaacuugCA.....      2      2      seq
...............................................cagugcaugCcagaaUuuggg.....      1      2      seq
...............................................cagugcaugacagaacuuggg.....      90     0      seq
...............................................cagugcaugCcagaacuuggU.....      6      2      seq
...............................................cagugUaugacagaacuuggg.....      2      1      seq
...............................................cagugcauAacagaacuuggA.....      1      2      seq
...............................................cagugcaugacagUacuuggU.....      1      2      seq
...............................................cagGgcaugacagaacuuggU.....      25     2      seq
...............................................cagugcaugacagaacuugUg.....      1      1      seq
...............................................cGgugcaugacagaacuuggg.....      2      1      seq
...............................................cGgugcaugacagaacuuggU.....      1      2      seq
...............................................cCgugcaugacagaacuugggU....      1      2      seq
...............................................cagGgcaugacagaacuugggU....      4      2      seq
...............................................cGgugcaugacagaacuugggA....      1      2      seq
...............................................cagugcaugacagaacuugggA....      33     1      seq
...............................................cagugcaugacagaacuugggU....      13     1      seq
...............................................cagugcaugacagaacuuggAU....      6      2      seq
...............................................cagugcaugacagaacuuggAA....      25     2      seq
...............................................cagGgcaugacagaacuugggA....      2      2      seq
...............................................cagugcaugacagaacuuggUU....      13     2      seq
...............................................cagugcaugacagaacuuggUA....      17     2      seq
...............................................cagugcaugacagaacuuggAG....      3      2      seq
...............................................cagugcaugacagaacuugggUA...      1      2      seq
...............................................cagugcaugacagaacuugggAU...      2      2      seq
...............................................cagugcaugacagaacuugggUU...      2      2      seq
................................................agugcaugacagaacuu........      3      0      seq
................................................agugcaugacagaacuuA.......      1      1      seq
................................................agugcaugacagaacuug.......      2      0      seq
................................................agugcaugacagaacuugg......      1777   0      seq
................................................agugcaugaUagaacuugg......      1      1      seq
................................................agugcaugacagaacuGgg......      1      1      seq
```

```
                                              mmu-miR-152-3p
       mmu-miR-152-5p
ccgggccuagguucugugauacacuccgacucgggcucuggagcagucagugcaugacagaacuugggcccgg

................................................aguCcaugacagaacuugg......   4     1     seq
................................................agugcauUacagaacuugg......   1     1     seq
................................................aguCcaugacagaacuugU......   1     2     seq
................................................agugcUugacagaacuugU......   1     2     seq
................................................agugcaugaAagaacuugg......   1     1     seq
................................................agugcaugacaCaacuugg......   4     1     seq
................................................agugcaugacagaacuugA......   6     1     seq
................................................agugcaugacagaacuugU......   9     1     seq
................................................agCgcaugacagaacuugg......   7     1     seq
................................................agugcaugacagUacuugg......   1     1     seq
................................................agugcUugCcagaacuugg......   1     2     seq
................................................UCugcaugacagaacuugg......   3     2     seq
................................................aCugcaugacagaacuugg......   7     1     seq
................................................Cgugcaugacagaacuugg......   1     1     seq
................................................agugcaugacGgaacuugg......   1     1     seq
................................................agugcaugacagaacCugg......   2     1     seq
................................................agugcaugacagaCcuugg......   3     1     seq
................................................agugcCugacagaacuugg......   13    1     seq
................................................agugcauUaUagaacuugg......   1     2     seq
................................................agugcaugacagaaUuugg......   2     1     seq
................................................agugcGugacagaacuugg......   11    1     seq
................................................agugcaugacaAaacuugg......   2     1     seq
................................................agugcaugacagaacuCgg......   1     1     seq
................................................agAgcaugacagaacuugg......   13    1     seq
................................................agugcaugacagaUcuugg......   9     1     seq
................................................agugcaugacagaGcuugg......   3     1     seq
................................................aUugcaugacagaacuugg......   1     1     seq
................................................agugcUugacagaacuugg......   8     1     seq
................................................aAugcaugacagaacuugg......   8     1     seq
................................................agugcaugacagaacuuAg......   1     1     seq
................................................Ugugcaugacagaacuugg......   1     1     seq
................................................agugcaugacagGacuugg......   1     1     seq
................................................agugcaugacagCacuugg......   16    1     seq
................................................agGgcaugacagaacuugg......   14    1     seq
................................................agugcUugacCgaacuugg......   1     2     seq
................................................agugcaugacaUaacuugg......   1     1     seq
................................................aCugcaugacagaacuuggA.....   2     2     seq
................................................agugcaugCcagaacuuggA.....   1     2     seq
................................................agugUaugacagaacuuggU.....   1     2     seq
................................................agugcGugacagaacuuggA.....   1     2     seq
................................................agugcUugacagaacuuggU.....   2     2     seq
................................................agAgcaugacagaacuuggg.....   1     1     seq
................................................aguUcaugacagaacuuggU.....   1     2     seq
................................................agugcaugacagaacAuggA.....   2     2     seq
................................................agugcaugacagaacuuggC.....   2     1     seq
................................................agGgcaugacagaacuuggU.....   2     2     seq
................................................agugcaugacagaaUuuggg.....   1     1     seq
................................................agGgcaugacagaacuuggA.....   3     2     seq
................................................agCgcaugacagaacuuggA.....   1     2     seq
................................................agugcaugaAagaacuuggg.....   1     1     seq
................................................agugcaugacagaacGuggU.....   1     2     seq
................................................agugcaugacagaUcuuggA.....   2     2     seq
................................................agugcaugacGgaacuuggA.....   1     2     seq
................................................agugcaugacaAaacuuggA.....   1     2     seq
................................................agugcaugacagaacuuggg.....   70    0     seq
................................................agugcaugacagaGcuuggU.....   1     2     seq
................................................agAgcaugacagaacuuggU.....   2     2     seq
................................................agugcUugacagaacuuggA.....   1     2     seq
................................................agugcCugacagaacuuggA.....   4     2     seq
................................................agugcaugacagCacuuggA.....   3     2     seq
................................................agugcaugacagaacuuggA.....   308   1     seq
................................................agugcaugacagaUcuuggU.....   1     2     seq
................................................aUugcaugacagaacuuggA.....   2     2     seq
................................................agugcUugacagaacuuggg.....   2     1     seq
................................................agugcaugacagaacuuggU.....   191   1     seq
................................................agugcaugacagaacCuggA.....   1     2     seq
................................................agGgcaugacagaacuuggg.....   2     1     seq
................................................aguAcaugacagaacuuggA.....   1     2     seq
................................................agugcauAacagaacuuggg.....   1     1     seq
```

```
                                              mmu-miR-152-3p
       mmu-miR-152-5p
ccgggccuagguucugugauacacuccgacucgggcucuggagcagucagugcaugacagaacuugggcccgg

................................................agugcaugacaCaacuuggA.....    2     2     seq
................................................agAgcaugacagaacuuggA.....    3     2     seq
................................................agugcaugacagaaUuugggA....    1     2     seq
................................................agugcaugacagaacuuggUU....    9     2     seq
................................................agugcaugacagaacuuggAA....    14    2     seq
................................................aUugcaugacagaacuugggU....    1     2     seq
................................................agugcaugacagaacuugggA....    18    1     seq
................................................agugcaugGcagaacuugggA....    1     2     seq
................................................agugcaugacagaacuugggG....    1     1     seq
................................................agugcaugacagaacuuggUA....    12    2     seq
................................................agugcaugacagaacuuggAU....    7     2     seq
................................................agugcaugacagaacuugggU....    5     1     seq
................................................agAgcaugacagaacuugggA....    1     2     seq
................................................agugcaugacagaacuuggAc....    1     1     seq
................................................agugcaugacagaacuuggAG....    4     2     seq
................................................agugcaugacagaacuugggAA...    2     2     seq
................................................agugcaugacagaacuugggUU...    2     2     seq
................................................agugcaugacagaacuugggUA...    1     2     seq
................................................agugcaugacagaacuugggAU...    2     2     seq
................................................agugcaugacagaacuugggAG...    1     2     seq
.................................................gugcaGgacagaacuugg......    1     1     seq
.................................................gugcaugacagaacuugg......    39    0     seq
.................................................Cugcaugacagaacuugg......    1     1     seq
.................................................gugcaugacagaacuuggU.....    4     1     seq
.................................................gugcaugacagaacuuggA.....    12    1     seq
.................................................gugcaugacagaacuuggAA....    1     2     seq
.................................................gugcaugacagaacuugggA....    1     1     seq
.................................................gugcaugacagaacuuggUU....    1     2     seq
.................................................gugcaugacagaacuuggUA....    2     2     seq
..................................................ugcaugacagaacuugg......    27    0     seq
..................................................ugcaugacagaacuuggg.....    1     0     seq
..................................................ugcaugacagaacuuggU.....    3     1     seq
..................................................ugcaugacagaacuuggA.....    1     1     seq
..................................................ugcaugacagaacuugggA....    1     1     seq
...................................................gcaugacagaacuuggU.....    4     1     seq
...................................................gcaugacagaacuuggg.....    4     0     seq
...................................................gcaugaUagaacuuggg.....    1     1     seq
...................................................gcaugacagaacuuggA.....    4     1     seq
...................................................gcaugacagaacuuggAA....    3     2     seq
...................................................gcaugacagaacuuggUU....    2     2     seq
...................................................gcaugacagaacuugggAU...    1     2     seq
.....................................................auAaUagaacuugggcc...    1     2     seq
.....................................................augacagaacuugggUA...    1     2     seq
```
