# Supplementary material for: mirPRo–a novel standalone program for differential expression and variation analysis of miRNAs
Source: Sci Rep. 2015 Oct 5;5:14617. doi: 10.1038/srep14617 (PMC4592965; doi:10.1038/srep14617)
Supplement: Supplementary Data 12-21 [file srep14617-s25.zip › Supplementary Data 16.pdf]

miRBase precursor : hsa-mir-324
Total read count : 1146
hsa-miR-324-5p read count : 954
hsa-miR-324-3p read count : 189
remaining reads : 3

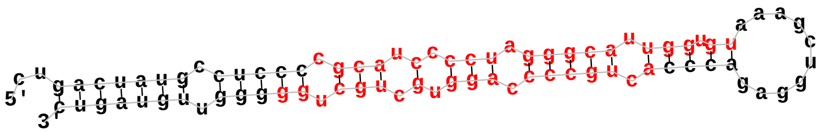

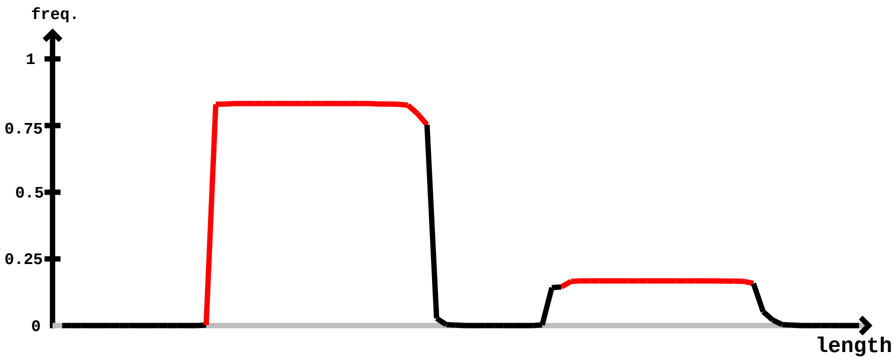


```
                                                            hsa-miR-324-3p
                   hsa-miR-324-5p
5'- cugacuaugccuccccgcauccccuagggcauuggugugaaagcuggagacccacugccccaggugcugcugggggguuguaguc   -3'    exp
    ..(((((((.(((((.(((.(.(((.(((((.(((.((..........))))).))))).))).).))).))))).)))))))   reads     mm        sample
    ..............ccgcauccccuagggcauuggugu.............................................   1         0         seq
    ..............ccgcauccccuagggcauuggugua............................................   1         0         seq
    ...............cgcauccccuagggcau...................................................   1         0         seq
    ...............cgcauccccuaAggcau...................................................   1         1         seq
    ...............cgcauccccuagggcauug.................................................   1         0         seq
    ...............cgcauccccuagggcauugg................................................   4         0         seq
    ...............cgcauccccuagggcauuggC...............................................   6         1         seq
    ...............cgcauccccuagggcauuggu...............................................   29        0         seq
    ...............cgcauccccuagUgcauuggu...............................................   1         1         seq
    ...............cgcauccccuagggcauugguA..............................................   4         1         seq
    ...............Ugcauccccuagggcauuggug..............................................   1         1         seq
    ...............cgcauccccuaAggcauuggug..............................................   1         1         seq
    ...............cgcauccccuagggcauuggug..............................................   40        0         seq
    ...............cgcauccccuagggcauugguU..............................................   2         1         seq
    ...............cgcauccccuaggCAauuggugu.............................................   1         2         seq
    ...............cgcauccccuagggcauuCgugu.............................................   1         1         seq
    ...............cgcauccccuagggUauuggugu.............................................   1         1         seq
    ...............cgcauccccuaAggcauuggugu.............................................   2         1         seq
    ...............cgcauccccuaggCcauuggugu.............................................   2         1         seq
    ...............cgcauccccuagggcauuggugG.............................................   4         1         seq
    ...............cgcauccccuaCggcauuggugu.............................................   1         1         seq
    ...............cgcauccccuagggcauugguAu.............................................   2         1         seq
    ...............cgcaucccUuagggcauuggugC.............................................   1         2         seq
    ...............cgcauccccuagggcauugUugC.............................................   1         2         seq
    ...............cgcauccccuagggcauugAugu.............................................   1         1         seq
    ...............cgcauccccuagggcauuggugC.............................................   126       1         seq
    ...............cgcauccccuagggcauuggCgu.............................................   1         1         seq
    ...............cgcaucccUuagggcauuggugu.............................................   1         1         seq
    ...............cgcauccccuagggcauuggugA.............................................   8         1         seq
    ...............cgUauccccuagggcauuggugu.............................................   1         1         seq
    ...............cgcauccccuagggcauuggugu.............................................   674       0         seq
    ...............cgcauccccuagggcauugguguC............................................   5         1         seq
    ...............cgcauccccuagggcauugguguG............................................   5         1         seq
    ...............cgcauccccuagggcauuggugua............................................   10        0         seq
```

```
                                                   hsa-miR-324-3p
               hsa-miR-324-5p
cugacuaugccucccCGCAUCCCCUAGGGCAUUGGUGUaaagcuggagacccACUGCCCCAGGUGCUGCUGGggguuguaguc
```

```
...............cgcaucccuagggcauugguguU............................................   6     1     seq
...............cgcaucccuagggcauugguguaC...........................................   2     1     seq
...............cgcaucccuagggcauugguguaUU..........................................   2     2     seq
................gcaucccuagggcauuggugA.............................................   1     1     seq
.................caucccuagggcauuggugu.............................................   2     0     seq
................................................ccAacugccccaggugcugcugg...........   1     1     seq
................................................cccacugccccaggugcugcugg...........   2     0     seq
.................................................ccacugccccaggugcug..............   1     0     seq
.................................................ccacugccccaggugcugcu.............   1     0     seq
.................................................ccacugccccaggugcugcug............   7     0     seq
.................................................ccacugcccUagUugcugcug............   1     2     seq
.................................................ccacugccccaggugcugcugU...........   7     1     seq
.................................................ccacugccccaggugcugcugg...........   106   0     seq
.................................................ccacugccccaggugcugcuggU..........   14    1     seq
.................................................ccacugccccaggugcugcuggA..........   8     1     seq
.................................................ccacugccccaggugcugcuggUU.........   1     2     seq
.................................................ccacugccccaggugcugcuggAC.........   1     2     seq
.................................................ccacugccccaggugcugcuggUA.........   2     2     seq
.................................................ccacugccccaggugcugcuggAA.........   1     2     seq
.................................................ccacugccccaggugcugcuggAU.........   6     2     seq
.................................................ccacugccccaggugcugcuggAgU........   2     2     seq
.................................................ccacugccccaggugcugcuggAAgu.......   2     2     seq
..................................................cacugccccaggugcugcugg...........   2     0     seq
..................................................AacugccccaggugcugcuggU..........   1     2     seq
...................................................acugccccaggugcugcug............   1     0     seq
...................................................acugccccaggugcugcugg...........   3     0     seq
...................................................acugccccaggugcugcuggC..........   2     1     seq
...................................................acugccccaggugcugcuggU..........   11    1     seq
...................................................acugccccaggugcugcuggUU.........   1     2     seq
...................................................acugccccaggugcugcuggUA.........   6     2     seq
....................................................cugccccaggugcugcuggAU.........   2     2     seq
```
