# Supplementary material for: mirPRo–a novel standalone program for differential expression and variation analysis of miRNAs
Source: Sci Rep. 2015 Oct 5;5:14617. doi: 10.1038/srep14617 (PMC4592965; doi:10.1038/srep14617)
Supplement: Supplementary Data 12-21 [file srep14617-s25.zip › Supplementary Data 17.pdf]

miRBase precursor : hsa-mir-324
Total read count : 2004
hsa-miR-324-5p read count : 1783
hsa-miR-324-3p read count : 218
remaining reads : 3

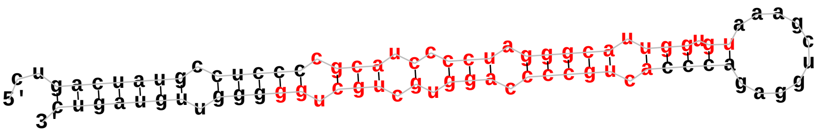

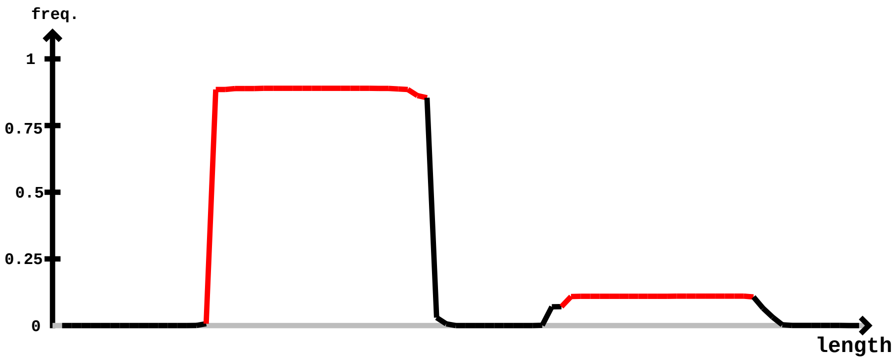


```
                                                                hsa-miR-324-3p
                   hsa-miR-324-5p
5'- cugacuaugccuccccgcauccccuagggcauuggugugaaagcuggagacccacugccccaggugcugcuggggguuguaguc   -3'    exp
    ..((((((.(((((.(((.(.(((.(((((.(((.((..........))))).))))).))).).))).))))).)))))))   reads      mm         sample
    .............cGcgcauccccuagggcauuggugC.............................................   1          2          seq
    ..............cGAcauccccuagggcauuggugu.............................................   2          2          seq
    ..............cGgcauccccuagggcauuggugu.............................................   3          1          seq
    ..............cGCcauccccuagggcauuggugu.............................................   6          2          seq
    ..............ccgcauccccuagggcauuggugu.............................................   1          0          seq
    ..............cGgcauccccuagggcauuggugC.............................................   1          2          seq
    ...............cgcauccccuagggcau...................................................   1          0          seq
    ...............cgcauccccuagggcauug.................................................   4          0          seq
    ...............cgcauccccuagggcauugg................................................   3          0          seq
    ...............cgcauccccuagggcauugU................................................   1          1          seq
    ...............Agcauccccuagggcauuggu...............................................   1          1          seq
    ...............cgcauccccuagggcauuAAu...............................................   1          2          seq
    ...............cgcauccccuaCggcauuggu...............................................   1          1          seq
    ...............cgcauccccuagggcauuggu...............................................   34         0          seq
    ...............cgcauccccuagggcauugUu...............................................   1          1          seq
    ...............cgcauccccuagggUauuggu...............................................   1          1          seq
    ...............cgcauccccuagggcauuggC...............................................   7          1          seq
    ...............cgcauccccuagggcauugguU..............................................   3          1          seq
    ...............cgcauccccuaCggcauuggug..............................................   1          1          seq
    ...............cgcauccccuagggcauugguA..............................................   3          1          seq
    ...............cgcauccccuagggcauuggug..............................................   9          0          seq
    ...............cgcauccccuagggcauugguAA.............................................   3          2          seq
    ...............cgcaucccUuagggcauuggugu.............................................   3          1          seq
    ...............cgcaGUcccuagggcauuggugu.............................................   1          2          seq
    ...............cgcauccAcuagggcauuggugu.............................................   1          1          seq
    ...............cgcauccccuagggcauuAgugu.............................................   8          1          seq
    ...............cgcauccccuagggUauuggugu.............................................   1          1          seq
    ...............cgcaucAccuagggcauuggugu.............................................   1          1          seq
    ...............cgcauccccuagggcauugUugu.............................................   1          1          seq
    ...............cgcauccccuaggCcauuggugu.............................................   1          1          seq
    ...............cgcauccccuaggUcauuggugu.............................................   1          1          seq
    ...............cgcauccccuagggcauAggugu.............................................   2          1          seq
    ...............cgcauccccuagAgcauuggugu.............................................   1          1          seq
    ...............cgcauccccuagggcauugguAu.............................................   5          1          seq
```

```
                                                   hsa-miR-324-3p
               hsa-miR-324-5p
cugacuaugccucccCgcauccccuagggcauugguguaaagcuggagacccacugccccaggugcugcuggggguuguaguc

...............cgcauccccuagggAauuggugu.............................................    1      1      seq
...............cgcauccccuagggGauuggugu.............................................    1      1      seq
...............cAcauccccuagggcauuggugC.............................................    1      2      seq
...............cgcaCccccuagggcauuggugu.............................................    1      1      seq
...............cgcauccccuagggcauugguUA.............................................    1      2      seq
...............cAcauccccuagggcauuggugu.............................................    5      1      seq
...............cgcauUcccuagggcauuggugu.............................................    1      1      seq
...............cgcauccccuaggCcauuggugC.............................................    1      2      seq
...............cgcauccccuagggcauuggugC.............................................    212    1      seq
...............cgcauccccuagggcauugguAC.............................................    1      2      seq
...............cgcauccccuagggcauuggugA.............................................    11     1      seq
...............Agcauccccuagggcauuggugu.............................................    1      1      seq
...............cgcauccUcuagggcauuggugu.............................................    3      1      seq
...............cgUauccccuagggcauuggugu.............................................    3      1      seq
...............cgcauccccuagggcauuggugu.............................................    1336   0      seq
...............cgcauccccuaAggcauuggugu.............................................    2      1      seq
...............cgcUCccccuagggcauuggugu.............................................    1      2      seq
...............cgcauccccuagggcauuUgugu.............................................    2      1      seq
...............cgcaucUccuagggcauuggugu.............................................    4      1      seq
...............cgcauccccuaCggcauuggugu.............................................    2      1      seq
...............cgcauccccuUgggcauuggCgu.............................................    1      2      seq
...............cgcauccccuGgggcauuggugu.............................................    1      1      seq
...............Ugcauccccuagggcauuggugu.............................................    2      1      seq
...............UgcauccccuagggcauuggugC.............................................    1      2      seq
...............cUcauccccuagggcauuggugu.............................................    1      1      seq
...............cgcaCUcccuagggcauuggugu.............................................    1      2      seq
...............cgcauAcccuagggcauuggugu.............................................    2      1      seq
...............cgcauccccuagUgcauuggugu.............................................    2      1      seq
...............cgcauccccuagggcauugguguU............................................    19     1      seq
...............cgcauccccuagggcauuggugCa............................................    1      1      seq
...............cgcauccccuagggcauugguguG............................................    2      1      seq
...............cgcauccccuagggcauuggugua............................................    20     0      seq
...............cgcauccccuagggcauugguguC............................................    4      1      seq
...............cgcauccccuagggcauuggugua U..........................................    3      1      seq
...............cgcauccccuagggcauugguguGU...........................................    1      2      seq
...............cgcauccccuagggcauugguguUU...........................................    4      2      seq
...............cgcauccccuagggcauuggugua C..........................................    5      1      seq
.................cauccAcuagggcauuggugu.............................................    1      1      seq
.................cGuccccuagggcauuggugu.............................................    1      1      seq
.................cauccccuagggcauuggugC.............................................    1      1      seq
.................cauccccuagggcauuggugu.............................................    4      0      seq
....................ccccuagggcauuggugu.............................................    1      0      seq
....................ccccuagggcauugguguU............................................    1      1      seq
.................................................ccAacugccccaggugcugcug............    1      1      seq
.................................................cccacugccccaggugcugcugg...........    1      0      seq
..................................................ccacugccccaggugcugcug............    5      0      seq
..................................................ccacugccccaggugcugcuAA...........    1      2      seq
..................................................ccacugccccaggugcugcugC...........    1      1      seq
..................................................ccacugccccagAugcugcugg...........    2      1      seq
..................................................ccacugccccaggugcugcugU...........    5      1      seq
..................................................ccacugccccaggugcugcugg...........    70     0      seq
..................................................ccacugccccaggugcugcuggU..........    18     1      seq
..................................................ccacugccccaggugcugcGUgg..........    1      2      seq
..................................................ccacugccccaggugcugcuggC..........    2      1      seq
..................................................ccacugccccaggugcugcuggA..........    11     1      seq
..................................................ccacugccccaggugcugcuggAA.........    7      2      seq
..................................................ccacugccccaggugcugcuggUU.........    4      2      seq
..................................................ccacugccccaggugcugcuggAU.........    7      2      seq
..................................................ccacugccccaggugcugcuggUC.........    1      2      seq
..................................................ccacugccccaggugcugcuggUgU........    1      2      seq
..................................................ccacugccccaggugcugcuggAAg........    1      2      seq
..................................................ccacugccccaggugcugcuggAgU........    1      2      seq
..................................................ccacugccccaggugcugcuggAUg........    1      2      seq
...................................................cacugccccaggugcugcuggU..........    1      1      seq
....................................................acugccccaggugcugcugg...........    5      0      seq
....................................................acugccccaggugcugcuggU..........    28     1      seq
....................................................acugccccaggugcugcuggC..........    5      1      seq
....................................................acugccccaggugcugcuggA..........    1      1      seq
....................................................acugccccaggugcugcuggUC.........    2      2      seq
```

```
                                                    hsa-miR-324-3p
               hsa-miR-324-5p
cugacuaugccuccccgcauccccuagggcauuggugucaaagcuggagacccacugccccaggugcugcuggggguuguaguc

....................................................acugccccaggugcugcugAAg.........    1    2    seq
....................................................acugccccaggugcugcuggUA.........    15   2    seq
....................................................acugccccaggugcugcuggUU.........    16   2    seq
....................................................acugccccaggugcugcuggAA.........    2    2    seq
....................................................acugccccaggugcugcuggUAg........    1    2    seq
.....................................................cugccccaggugcugcuggUU.........    2    2    seq
...............................................................GgcugcugggggCuguag..    1    2    seq
```
