# Supplementary material for: mirPRo–a novel standalone program for differential expression and variation analysis of miRNAs
Source: Sci Rep. 2015 Oct 5;5:14617. doi: 10.1038/srep14617 (PMC4592965; doi:10.1038/srep14617)
Supplement: Supplementary Data 12-21 [file srep14617-s25.zip › Supplementary Data 18.pdf]

miRBase precursor : hsa-mir-324
Total read count : 1471
hsa-miR-324-5p read count : 1181
hsa-miR-324-3p read count : 284
remaining reads : 6

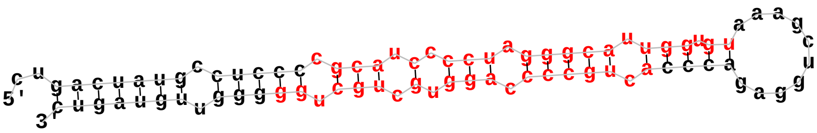

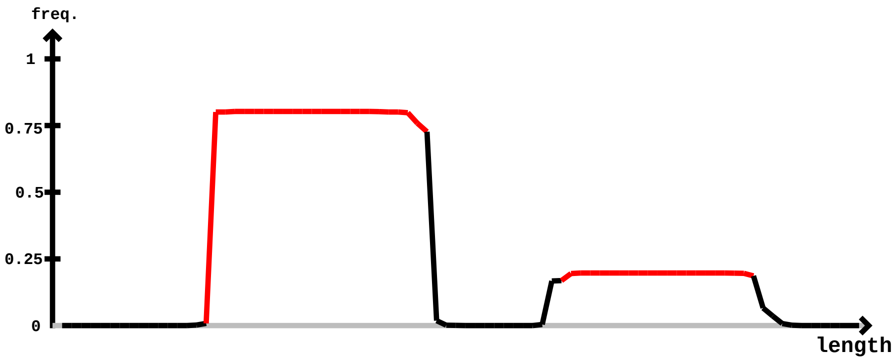


```
                                                        hsa-miR-324-3p
                   hsa-miR-324-5p
5'- cugacuaugccuccccgcauccccuagggcauuggguguaaagcuggagacccacugccccaggugcugcuggggguuguaguc   -3'    exp
    ..((((((.(((((.(((.(.(((.(((((.(((.((..........))))).))))).))).).))).))))).)))))))   reads      mm         sample
    .............cGcgcauccccuagggcauuggugu.............................................   1          1          seq
    .............cGUgcauccccuagggcauuggugu.............................................   2          2          seq
    ..............ccgcauccccuagggcauugU................................................   1          1          seq
    ..............ccgcauccccuagggcauuggug..............................................   1          0          seq
    ..............ccgcauccccuaAggcauuggugu.............................................   1          1          seq
    ..............cGgcauccccuagggcauuggugu.............................................   1          1          seq
    ..............ccgcauccccuagggcauuggugu.............................................   1          0          seq
    ..............Ucgcauccccuagggcauuggugu.............................................   2          1          seq
    ..............GcgcauccccuaAggcauuggugu.............................................   1          2          seq
    ..............cGUcauccccuagggcauuggugu.............................................   1          2          seq
    ...............cgcauccccuagggcau...................................................   1          0          seq
    ...............cgcauccccuagggcauu..................................................   2          0          seq
    ...............cgcauccccuagggcauugg................................................   3          0          seq
    ...............cgcauccccuagggcauuggu...............................................   46         0          seq
    ...............cgcauccccuaAggcauuggu...............................................   1          1          seq
    ...............cgcauccccuaggUcauuggu...............................................   1          1          seq
    ...............cgcauccccuagggcauuggC...............................................   10         1          seq
    ...............cgcauccccuagAgcauuggug..............................................   1          1          seq
    ...............cgcauccccuagggcauugguU..............................................   4          1          seq
    ...............Ugcauccccuagggcauuggug..............................................   1          1          seq
    ...............cgcauccccuagggcauuggug..............................................   30         0          seq
    ...............cgcauccccuagggcauugguC..............................................   2          1          seq
    ...............cgcauccccuagggcauugguA..............................................   8          1          seq
    ...............cgcauccccuaggCcauuggugu.............................................   5          1          seq
    ...............cgcauccccuaggCcauuggugC.............................................   1          2          seq
    ...............cgcauccccuaCggcauuggugu.............................................   3          1          seq
    ...............cgcauccccuaAggcauuggugu.............................................   3          1          seq
    ...............cgcaucccUuagggcauuggugu.............................................   2          1          seq
    ...............cgcauccccuaggCGauuggugu.............................................   1          2          seq
    ...............cgcauccccuagggcauugguAA.............................................   1          2          seq
    ...............cgcauccccuagggcauuggugC.............................................   178        1          seq
    ...............cUcauccccuagggcauuggugu.............................................   1          1          seq
    ...............cgcauccccuagggcauuggugA.............................................   2          1          seq
    ...............cgcauccccuagAgcauuggugu.............................................   1          1          seq
```

```
                                                    hsa-miR-324-3p
               hsa-miR-324-5p
cugacuaugccuccccgcauccccuagggcauuggugu aaagcuggagacccacugccccaggugcugcuggggguuguaguc

...............cgcauccccuagggUauuggugu.............................................    1      1      seq
...............cgcauccccuagggcauuggugu.............................................    822    0      seq
...............cgcauccccuGgggcauuggugu.............................................    1      1      seq
...............cgcauccccuagggcauuggCgu.............................................    1      1      seq
...............cgcauccccuaAggcauuggugC.............................................    1      2      seq
...............cgcauccccuagggcauugguAu.............................................    2      1      seq
...............cgcauccccuagggcauuggugG.............................................    2      1      seq
...............cgcauccccuagggcGuuggugu.............................................    1      1      seq
...............cgcauccccuagggcauuggugua............................................    6      0      seq
...............cgcauccccuagggcauuggugAU............................................    1      2      seq
...............cgcauccccuagggcauugguguU............................................    11     1      seq
...............cgcauccccuagggcauugguguC............................................    4      1      seq
...............cgcauccccuagggcauugguguG............................................    1      1      seq
...............cgcauccccuagggcauuggugAG............................................    2      2      seq
...............cgcauccccuagggcauugguguaC...........................................    1      1      seq
...............cgcauccccuagggcauugguguaUU..........................................    1      2      seq
.................cauccccuagggcauuggugC.............................................    2      1      seq
.................cauccccuagggcauuggugu.............................................    1      0      seq
.................................................ccAGcugccccaggugcugcugg...........    1      2      seq
.................................................ccGacugccccaggugcugcugg...........    1      1      seq
.................................................cccacugccccaggugcugcugg...........    1      0      seq
.................................................ccAacugccccaggugcugcugg...........    1      1      seq
.................................................ccAUcugccccaggugcugcugg...........    2      2      seq
..................................................ccacugccccaggugcugc..............    1      0      seq
..................................................ccacugccccaggugcugcu.............    1      0      seq
..................................................ccacugccccaggugcugcug............    13     0      seq
..................................................ccacugccccaggugcugcCU............    1      2      seq
..................................................ccacugccccaggugcugcugg...........    162    0      seq
..................................................ccacugccccaggugcugcugU...........    4      1      seq
..................................................ccacugccccaggugcugcugA...........    1      1      seq
..................................................ccacugccccaggugcugcuCgg..........    1      1      seq
..................................................ccacugccccaggugcugcCUgg..........    1      2      seq
..................................................ccacugccccaggugcugcuggA..........    14     1      seq
..................................................ccacugccccaggugcugcuggC..........    6      1      seq
..................................................ccacugccccaggugcugcuggU..........    10     1      seq
..................................................ccacugccccaggugcugcuggAg.........    1      1      seq
..................................................ccacugccccaggugcugcuggAC.........    2      2      seq
..................................................ccacugccccaggugcugcuggUU.........    5      2      seq
..................................................ccacugccccaggugcugcuggUA.........    1      2      seq
..................................................ccacugccccaggugcugcuggAU.........    7      2      seq
..................................................ccacugccccaggugcugcuggAA.........    5      2      seq
..................................................ccacugccccaggugcugcuggAgU........    4      2      seq
...................................................cacugccccaggugcugcugg...........    1      0      seq
...................................................AacugccccaggugcugcuggU..........    1      2      seq
....................................................acugccccaggugcugcugg...........    2      0      seq
....................................................GcugccccaggugAugcugg...........    1      2      seq
....................................................acugccccaggugcugcuggU..........    9      1      seq
....................................................acugccccaggugcugcuggC..........    2      1      seq
....................................................acugccccaggugcugcuggUU.........    1      2      seq
....................................................acugccccaggugcugcuggAA.........    3      2      seq
....................................................acugccccaggugcugcuggUA.........    14     2      seq
....................................................acugccccaggugcugcuggAgU........    1      2      seq
....................................................acugccccaggugcugcuggUAg........    4      2      seq
....................................................acugccccaggugcugcuggUAgu.......    1      2      seq
....................................................acugccccaggugcugcuggAUgu.......    1      2      seq
.....................................................cugccccaggugcugcuggAU.........    3      2      seq
```
