# Supplementary material for: mirPRo–a novel standalone program for differential expression and variation analysis of miRNAs
Source: Sci Rep. 2015 Oct 5;5:14617. doi: 10.1038/srep14617 (PMC4592965; doi:10.1038/srep14617)
Supplement: Supplementary Data 12-21 [file srep14617-s25.zip › Supplementary Data 19.pdf]

miRBase precursor : hsa-mir-324
Total read count : 740
hsa-miR-324-5p read count : 635
hsa-miR-324-3p read count : 101
remaining reads : 4

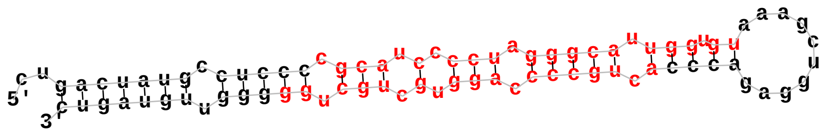

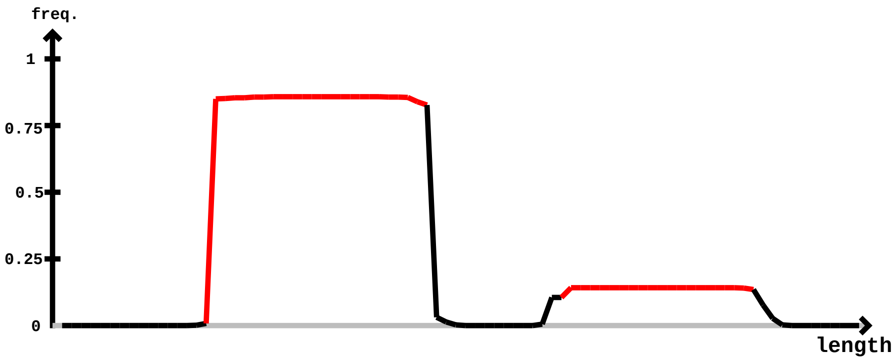

hsa-miR-324-3p

hsa-miR-324-5p

```
5'- cugacuaugccucccCGCAUCCCCUAGGGCAUUGGUGUaaagcuggagacccACUGCCCCAGGUGCUGCUGGggguuguaguc   -3'    exp
    ..(((((((.(((((.(((.(.(((.(((((.(((.((..........))))).))))).))).).))).))))).)))))))    reads      mm         sample
    .............cGcgcauccccuagggcauuggugu.............................................    1          1          seq
    ..............cGCcauccccuagggcauuggugu.............................................    1          2          seq
    ..............Acgcauccccuagggcauuggugu.............................................    1          1          seq
    ..............ccgcauccccuagggcauuggugu.............................................    2          0          seq
    ..............ccgcauccccuagggcauuggugC.............................................    1          1          seq
    ...............cgcauccccuCggAcauu..................................................    1          2          seq
    ...............cgcauccUcuagggcauugg................................................    1          1          seq
    ...............cgcauccccuagggcauuggu...............................................    12         0          seq
    ...............cgcauccccuagggcauugguU..............................................    1          1          seq
    ...............cgcauccccuagggcauuggug..............................................    7          0          seq
    ...............cgcauccccuagggcauugguA..............................................    1          1          seq
    ...............cgcauccccuaggCcauuggugC.............................................    1          2          seq
    ...............cgcauccccuaCggcauuggugu.............................................    1          1          seq
    ...............cgcauccccuagAgcauuggugu.............................................    1          1          seq
    ...............cgcUCccccuagggcauuggugu.............................................    1          2          seq
    ...............cgcaucccUuagggcauuggugu.............................................    1          1          seq
    ...............cgcaCccccuagggcauuggugu.............................................    1          1          seq
    ...............cgcauccccuaggUcauuggugA.............................................    1          2          seq
    ...............cgcauccccuagggcauugguAu.............................................    1          1          seq
    ...............cgcauccccuagggcauuggugA.............................................    3          1          seq
    ...............cgUauccccuagggcauuggugu.............................................    2          1          seq
    ...............cgcauccccuagggcauuggugG.............................................    2          1          seq
    ...............cgcauccccuagggcauuggugu.............................................    457        0          seq
    ...............cgcauccccuagggcauugguAA.............................................    3          2          seq
    ...............cgcauccccuaAggcauuggugu.............................................    2          1          seq
    ...............cgcauccccuaggCcauuggugu.............................................    7          1          seq
    ...............cgcaucUccuagggcauuggugu.............................................    1          1          seq
    ...............cgcauccUcuagggcauuggugu.............................................    1          1          seq
    ...............cgcauccccuaggCGauuggugu.............................................    1          2          seq
    ...............cgcauccccuaggAcUuuggugu.............................................    1          2          seq
    ...............cgcauccccuagggcauuggugC.............................................    87         1          seq
    ...............cgcauccccuaAggcauuggugC.............................................    1          2          seq
    ...............cgcaucccGuagggcauuggugC.............................................    1          2          seq
    ...............cgcauccccuagggcauugguguC............................................    2          1          seq
```

hsa-miR-324-3p

hsa-miR-324-5p

cugacuaugccuccccgcauccccuagggcauugguguaaagcuggagacccacugccccaggugcugcuggggguuguaguc

...............cgcauccccuaAggcauuggugua..	1	1	seq
...............cgcauccccuagggcauuggugua..	7	0	seq
...............cgcauccccuagggcauugguguU..	3	1	seq
...............cgcauccccuagggcauugguguaa...	1	0	seq
...............cgcauccccuagggcauugguguUa...	1	1	seq
...............cgcauccccuagggcauugguguaU...	2	1	seq
...............cgcauccccuagggcauugguguUU...	3	2	seq
...............cgcauccccuagggcauugguguGU...	1	2	seq
...............cgcauccccuagggcauugguguaaU..	1	1	seq
...............cgcauccccuagggcauugguguUaU..	1	2	seq
................gcauccccuagggcauuggugu...	1	0	seq
.................cauccccuagggcauuggugu...	2	0	seq
....................Ccccuagggcauuggugu...	2	1	seq
.....................cccuagggcauuggugu...	1	0	seq
....ccAGcugccccaggugcugcugg...........	2	2	seq
....ccACcugccccaggugcugcugg...........	1	2	seq
....cccacugccccaggugcugcuggU..........	1	1	seq
....ccacugccccaggugcugcu.............	1	0	seq
....ccacugccccaggugcugcug............	3	0	seq
....ccacugccccaggugcugcuUA...........	1	2	seq
....ccacugccccaggugcugcugg...........	35	0	seq
....ccacugccccaggugcugcugU...........	3	1	seq
....ccacugccccaggugcugcugA...........	1	1	seq
....ccacugccccaggugcugcuggU..........	9	1	seq
....ccacugccccaggugcugcCUgg..........	1	2	seq
....ccacugccccaggugcugcuggA..........	13	1	seq
....ccacugccccaggugcugcuggUU.........	1	2	seq
....ccacugccccaggugcugcuggAU.........	3	2	seq
....ccacugccccaggugcugcuggAA.........	1	2	seq
....ccacugccccaggugcugcuggAgU........	1	2	seq
....ccacugccccaggugcugcuggUAg........	1	2	seq
....acugccccaggugcugcug............	1	0	seq
....acugccccaggugcugcuggU..........	11	1	seq
....acugccccaggugcugcuggC..........	2	1	seq
....acugccccaggugcugcuggUA.........	7	2	seq
....acugccccaggugcugcuggUU.........	6	2	seq
