# Supplementary material for: mirPRo–a novel standalone program for differential expression and variation analysis of miRNAs
Source: Sci Rep. 2015 Oct 5;5:14617. doi: 10.1038/srep14617 (PMC4592965; doi:10.1038/srep14617)
Supplement: Supplementary Data 12-21 [file srep14617-s25.zip › Supplementary Data 20.pdf]

miRBase precursor : hsa-mir-324
Total read count : 2222
hsa-miR-324-5p read count : 1862
hsa-miR-324-3p read count : 356
remaining reads : 4

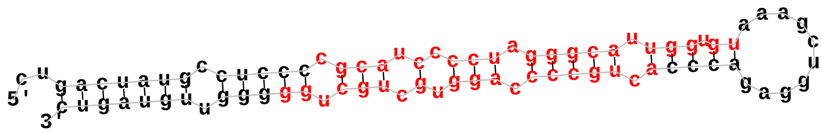

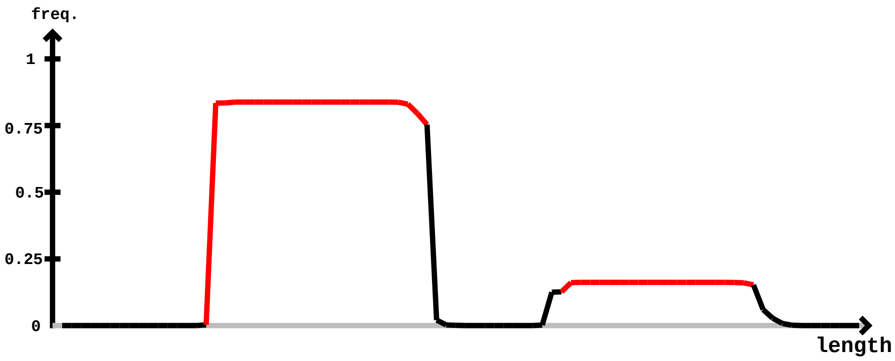


```
                                                                                   hsa-miR-324-3p
                            hsa-miR-324-5p
5'- cugacuaugccucccgcauccccuagggcauugguguaaagcuggagacccacugccccaggugcugcuggggguuguaguc   -3'    exp
    ..(((((((.(((((.(((.(.(((.(((((.(((.((..........))))).))))).))).).))).))))).)))))))    reads    mm     sample
    .............ccgcauccccuagggcauuggugu..............................................    1        0      seq
    .............cGgcauccccuagggcauuggugu..............................................    2        1      seq
    .............cGCcauccccuagggcauuggugu..............................................    2        2      seq
    .............ccgcauccccuaAggcauuggugu..............................................    1        1      seq
    ..............cgcauccccuagggcauug..................................................    1        0      seq
    ..............cgcauccccuagggcauugg.................................................    15       0      seq
    ..............cgcauccccuaAggcauugg.................................................    1        1      seq
    ..............cgcauccccuagggcauuggC................................................    11       1      seq
    ..............cgcauccccuagggcauuggu................................................    66       0      seq
    ..............cgcauccccuaCggcauuggu................................................    1        1      seq
    ..............cgcauccccuagggcauuggug...............................................    74       0      seq
    ..............cgcauccccuagggcauugguU...............................................    2        1      seq
    ..............cgcauccccuaggCcauuggug...............................................    1        1      seq
    ..............cgcauccccuagggcauugguA...............................................    15       1      seq
    ..............cgcauccccuagggcauuggugu..............................................    1336     0      seq
    ..............cgcaucccUuagggcauuggugu..............................................    2        1      seq
    ..............cgcauccccuaggCcauuggugu..............................................    1        1      seq
    ..............cgcauccccuaggAcauuggugC..............................................    1        2      seq
    ..............cgcauccccuGgggcauuggugu..............................................    1        1      seq
    ..............UgcauccccuagggcauuggugC..............................................    1        2      seq
    ..............cgcauccUcuagggcauuggugC..............................................    1        2      seq
    ..............cgcauccccuagggcauugUugu..............................................    1        1      seq
    ..............cgcauccccuagggcauuggugA..............................................    16       1      seq
    ..............cgcauccccuagggcaCuggugu..............................................    1        1      seq
    ..............cgcauccccCUgggcauuggugu..............................................    1        2      seq
    ..............cAcauccccuagggcauuggugu..............................................    3        1      seq
    ..............cgcauccccuagggcauuggugG..............................................    5        1      seq
    ..............cgcauccccuagAgcauuggugu..............................................    2        1      seq
    ..............cgcauccccuagggcauugguUu..............................................    2        1      seq
    ..............cgcauccccuagggcauugguAu..............................................    2        1      seq
    ..............cgcauccccuagggcauugAugu..............................................    1        1      seq
    ..............cgcauccccuaAggcauuggugu..............................................    1        1      seq
    ..............cgcauccccuagggcauuggugC..............................................    234      1      seq
    ..............Ugcauccccuagggcauuggugu..............................................    1        1      seq
```

```
                                                   hsa-miR-324-3p
               hsa-miR-324-5p
cugacuaugccuccccgcauccccuagggcauuggugugaaagcuggagacccacugccccaggugcugcuggggguuguaguc

...............cgUauccccuagggcauuggugu.............................................    2     1     seq
...............cgcauccccuagggcauugguguG............................................    7     1     seq
...............cgcauccccuagggcauugguguC............................................    7     1     seq
...............cgcauccccuagggcauuggugAG............................................    1     2     seq
...............cgcauccccuagggcauuggugAa............................................    1     1     seq
...............cgcauccccuagggcauuggugua............................................    6     0     seq
...............cgcauccccuagggcauugguguU............................................    19    1     seq
...............cgcauccccuagggcauugguguaC...........................................    3     1     seq
...............cgcauccccuagggcauugguguaaa..........................................    2     0     seq
................Ucauccccuagggcauuggugu.............................................    1     1     seq
.................cauccccuagggcauuggugu.............................................    7     0     seq
.................................................ccAacugccccaggugcugcugg...........    1     1     seq
.................................................Gccacugccccaggugcugcugg...........    1     1     seq
.................................................ccACcugccccaggugcugcugg...........    1     2     seq
.................................................ccGacugccccaggugcugcuggA..........    1     2     seq
..................................................ccacugccccaggugcugc..............    1     0     seq
..................................................ccacugccccaggugcugcu.............    3     0     seq
..................................................ccacugccccaggugcugcug............    14    0     seq
..................................................ccacugccccaggugcugcuA............    3     1     seq
..................................................ccacugccccaggugcugcugg...........    188   0     seq
..................................................ccacugccccaggugcugcugA...........    3     1     seq
..................................................ccacugccccaggugcugcugU...........    4     1     seq
..................................................ccacugccccaggugcugcuggA..........    13    1     seq
..................................................ccacugccccaggugcugcuggU..........    17    1     seq
..................................................ccacugccccaggugcugcuggC..........    3     1     seq
..................................................ccacugccccaggugcugcuggAg.........    1     1     seq
..................................................ccacugccccaggugcugcuggUA.........    5     2     seq
..................................................ccacugccccaggugcugcuggAU.........    7     2     seq
..................................................ccacugccccaggugcugcuggUU.........    2     2     seq
..................................................ccacugccccaggugcugcuggAA.........    1     2     seq
..................................................ccacugccccaggugcugcuggAgU........    7     2     seq
..................................................ccacugccccaggugcugcuggAAgu.......    2     2     seq
...................................................cacugccccaggugcugcugg...........    3     0     seq
....................................................acugccccaggugcugcugg...........    5     0     seq
....................................................acugccccaggugcugcuggU..........    36    1     seq
....................................................acugccccaggugcugcuggC..........    2     1     seq
....................................................acugccccaggugcugcuggAU.........    2     2     seq
....................................................acugccccaggugcugcuggUU.........    3     2     seq
....................................................acugccccaggugcugcuggUA.........    20    2     seq
....................................................acugccccaggugcugcuggAA.........    1     2     seq
....................................................acugccccaggugcugcuggUAg........    6     2     seq
....................................................acugccccaggugcugcuggUgU........    1     2     seq
....................................................acugccccaggugcugcuggUAgu.......    1     2     seq
.....................................................cugccccaggugcugcuggAU.........    1     2     seq
.....................................................cugccccaggugcugcuggUAg........    1     2     seq
```
