# Supplementary material for: mirPRo–a novel standalone program for differential expression and variation analysis of miRNAs
Source: Sci Rep. 2015 Oct 5;5:14617. doi: 10.1038/srep14617 (PMC4592965; doi:10.1038/srep14617)
Supplement: Supplementary Data 12-21 [file srep14617-s25.zip › Supplementary Data 21.pdf]

miRBase precursor : hsa-mir-324
Total read count : 1588
hsa-miR-324-5p read count : 1344
hsa-miR-324-3p read count : 240
remaining reads : 4

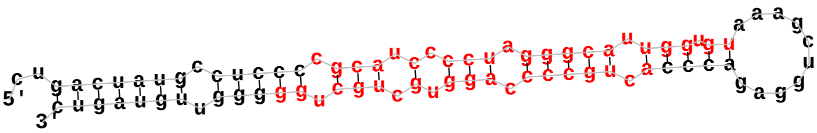

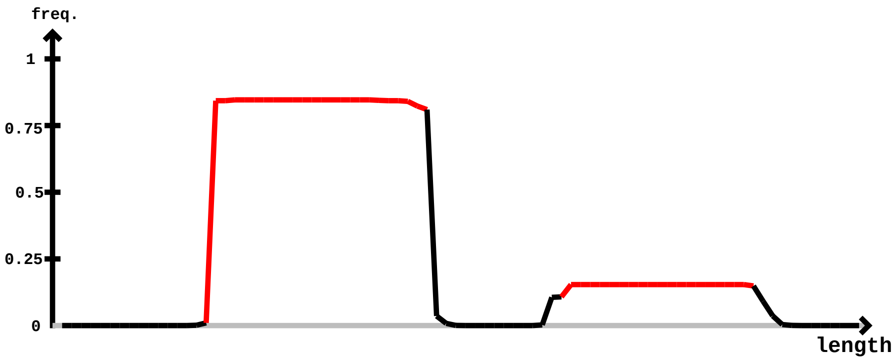

hsa-miR-324-3p

hsa-miR-324-5p

5'- cugacuaugccuccccgcauccccuagggcauugguguaaagcuggagacccacugccccaggugcugcuggggguuguaguc -3'	exp		
..(((((((.(((((.(((.(.(((.(((((.(((.((..........))))).))))).))).).))).))))).)))))))	reads	mm	sample
.............cGcgcauccccuagggcauuggugC...	1	2	seq
.............cGcgcauccccuagggcauugguguU..	1	2	seq
..............ccgcauccccuaCggcaA...	1	2	seq
..............cGgcauccccuagggcauuggugC...	1	2	seq
..............cGUcauccccuagggcauuggugu...	1	2	seq
..............ccgcauccccuaCggcauuggugC...	1	2	seq
..............cGAcauccccuagggcauuggugu...	1	2	seq
..............cGCcauccccuagggcauuggugu...	4	2	seq
..............cGgcauccccuagggcauuggugu...	5	1	seq
...............cgcauccccuagggcau...	2	0	seq
...............cgcauccccuagggcaGu..	1	1	seq
...............cgcauccccuagggcauu..	1	0	seq
...............cgcauccccuaCggcauugg..	1	1	seq
...............cgcauccccuagggcauugg..	3	0	seq
...............cgcauccccCUgggcauuggu...	1	2	seq
...............cgcauccccuagggAauuggu...	1	1	seq
...............Ugcauccccuagggcauuggu...	1	1	seq
...............cgcauccccuaggCcauuggu...	1	1	seq
...............cgcGuccccuagggcauuggu...	1	1	seq
...............cgcauccccuagggcauuggu...	23	0	seq
...............cgcauccccuaggCcauuggug..	1	1	seq
...............cgcauccccuagggcauugguU..	3	1	seq
...............cgcauccccuagggcauuggug..	17	0	seq
...............cgcaAccccuagggcauuggugu...	1	1	seq
...............cgcauccccuGgggcauuggugu...	1	1	seq
...............cgcauccUcuagggcauuggugu...	3	1	seq
...............cgcauccccuagggcauAggugu...	5	1	seq
...............cgcaucccUuagggcauuggugu...	2	1	seq
...............cgcauccccuagggcauuAgugu...	4	1	seq
...............cgcauccccuUgggcauuggugC...	2	2	seq
...............cgcauccccuagggcauuggCgC...	1	2	seq
...............cgcauccccuagggGauuggugu...	2	1	seq
...............Ugcauccccuagggcauuggugu...	2	1	seq
...............cgcauccccuagggUauuggugC...	2	2	seq

hsa-miR-324-3p			
hsa-miR-324-5p			
cugacuaugccucccCGCAUCCCCUAGGGCAUUGGUGUaaagcuggagacccACUGCCCCAGGUGCUGCUGGggguuguagc			
...............cgcauAcccuagggcauuggugu..	2	1	seq
...............cgcauccccuaggCGauuggugu..	1	2	seq
...............cgcauccccuagggcauugAugu..	1	1	seq
...............cgUauccccuaggCcauuggugu..	1	2	seq
...............cgcaucUccuagggcauuggugu..	2	1	seq
...............cgcauccccuagggcauuCgugu..	1	1	seq
...............cgcauccccuagggcauugguUG..	1	2	seq
...............cgcauccccuagggcauugguAu..	3	1	seq
...............cgcauccccuagggcauuggugu..	982	0	seq
...............cgcauccccuagggcauuAgugC..	1	2	seq
...............cgcauccccuagggcauugUugu..	1	1	seq
...............cgcauccccuaggUcauuggugu..	1	1	seq
...............cgcauccccuaAggAauuggugu..	1	2	seq
...............cgcauccAcuagggcauuggugu..	1	1	seq
...............cAcauccccuagggcauuggugu..	4	1	seq
...............cgcauccccuaAggcauuggugu..	1	1	seq
...............cgcauccccuGggUcauuggugu..	1	2	seq
...............cgcauccccuaCggcauuggugu..	2	1	seq
...............cgcauccccuagggcauuggugC..	139	1	seq
...............cgcauccccuaggCcauuggugC..	1	2	seq
...............cgcauccccuagggcauuggugA..	9	1	seq
...............cgcauccccuagggUauuggugu..	1	1	seq
...............cgcauccccuaggCcauuggugu..	11	1	seq
...............cAcauccccAagggcauuggugu..	1	2	seq
...............cgcauccccuagggcauugguAA..	5	2	seq
...............cgcaucGccuagggcauuggugu..	2	1	seq
...............cgcauccccuUgggcauuggugu..	1	1	seq
...............cgcauccccuUAggcauuggugu..	1	2	seq
...............cgcauccccuagggcauuggCgu..	2	1	seq
...............cgcaucUccuagggcauuggugC..	1	2	seq
...............cgcauccccuagggcauuggugG..	4	1	seq
...............cgcauccccuagggcauugguguC...	4	1	seq
...............cgcauccccuagggcauugguguG...	1	1	seq
...............cgcauUcccuaggUcauuggugua...	1	2	seq
...............cgcauccccuagggcauuggugua...	19	0	seq
...............cgcauccccuagggcauugguguU...	16	1	seq
...............cgcauccccuagggcauuggugGC...	1	2	seq
...............cgcauccccuagggUauugguguC...	1	2	seq
...............cgcauccccuagggcauuggugua..	3	0	seq
...............cgcauccccuagggcauuggugua C..	2	1	seq
...............cgcauccccuagggcauugguguaU..	2	1	seq
...............cgcauccccuagggcauugguguUU..	5	2	seq
...............cgcauccccuagggcauugguguUaU...	1	2	seq
.................cauccccuagggcauugguAu..	1	1	seq
.................cauccccuagggcauuggugu..	4	0	seq
....cccacugccccaggugcugcugg..........	2	0	seq
....ccACcugccccaggugcugcugg..........	1	2	seq
....Uccacugccccaggugcugcugg..........	1	1	seq
....ccacugccccaggugcugcug...........	8	0	seq
....ccacugccccaggugcugcugU..........	4	1	seq
....ccacugccccaggugcugcugg..........	78	0	seq
....ccacugccccaggugcugcugC..........	1	1	seq
....ccacugccccaUgugcugcugg..........	1	1	seq
....ccacugccccaggugcugcuggU.........	23	1	seq
....ccacugccccaggugcugcuggg.........	2	0	seq
....ccacugccccaggugcugcugUA.........	1	2	seq
....cGacugccccaggugcugcuggU.........	1	2	seq
....ccacugccccaggugcugcCUgg.........	1	2	seq
....ccacugccccaggugcugAuggU.........	1	2	seq
....ccacugccccaggugcugcuggA.........	16	1	seq
....ccacugccccaggugcugcuggC.........	1	1	seq
....ccacugccccaggugcugcugUU.........	1	2	seq
....ccacugccccaggugcugcuggAA........	4	2	seq
....ccacugccccaggugcugcuggUA........	4	2	seq
....ccacugccccaggugcugcuggAU........	8	2	seq
....ccacugccccaggugcugcuggUU........	6	2	seq
....ccacugccccaggugcugcuggUgU.......	2	2	seq
....ccacugccccaggugcugcuggAUgu......	1	2	seq
....cacugccccaggugcugcugg..........	1	0	seq

```
                                                    hsa-miR-324-3p
               hsa-miR-324-5p
cugacuaugccuccccgcauccccuagggcauugguguaaagcuggagacccacugccccaggugcugcuggggguuguaguc

...................................................cacugccccaggugcugcuggU..........    1           1           seq
...................................................cacugccccaggugcugcuggUU.........    1           2           seq
....................................................acugccccaggugcugcugg...........    1           0           seq
....................................................acugccccaggugcugcugU...........    1           1           seq
....................................................acugccccagguAcugcuggC..........    1           2           seq
....................................................acugccccaggugcugcuggC..........    4           1           seq
....................................................acugccccaggugcugcuggU..........    34          1           seq
....................................................acugccccaggugcugcuggUU.........    12          2           seq
....................................................acugccccaggugcugcuggAU.........    2           2           seq
....................................................acugccccaggugcugcuggUA.........    13          2           seq
....................................................acugccccaggugcugcuggUC.........    2           2           seq
....................................................acugccccaggugcugcuggUAg........    2           2           seq
....................................................acugccccaggugcugcuggUgA........    1           2           seq
```
